# Total synthesis of a *Streptococcus pneumoniae* serotype 12F CPS repeating unit hexasaccharide

**DOI:** 10.3762/bjoc.13.19

**Published:** 2017-01-25

**Authors:** Peter H Seeberger, Claney L Pereira, Subramanian Govindan

**Affiliations:** 1 Department of Biomolecular Systems, Max Planck Institute of Colloids and Interfaces, Am Mühlenberg 1, 14476 Potsdam, Germany; 2 Department of Chemistry and Biochemistry, Freie Universität Berlin, Arnimallee 22, 14195 Berlin, Germany; 3 Vaxxilon Deutschland GmbH, Magnusstrasse 11, 12489 Berlin, Germany

**Keywords:** carbohydrate antigen, glycosylation, oligosaccharides, *Streptococcus pneumoniae*, total synthesis

## Abstract

The Gram-positive bacterium *Streptococcus pneumoniae* causes severe disease globally. Vaccines that prevent *S. pneumoniae* infections induce antibodies against epitopes within the bacterial capsular polysaccharide (CPS). A better immunological understanding of the epitopes that protect from bacterial infection requires defined oligosaccharides obtained by total synthesis. The key to the synthesis of the *S. pneumoniae* serotype 12F CPS hexasaccharide repeating unit that is not contained in currently used glycoconjugate vaccines is the assembly of the trisaccharide β-D-Gal*p*NAc-(1→4)-[α-D-Glc*p*-(1→3)]-β-D-Man*p*NAcA, in which the branching points are equipped with orthogonal protecting groups. A linear approach relying on the sequential assembly of monosaccharide building blocks proved superior to a convergent [3 + 3] strategy that was not successful due to steric constraints. The synthetic hexasaccharide is the starting point for further immunological investigations.

## Introduction

**Figure 1:**
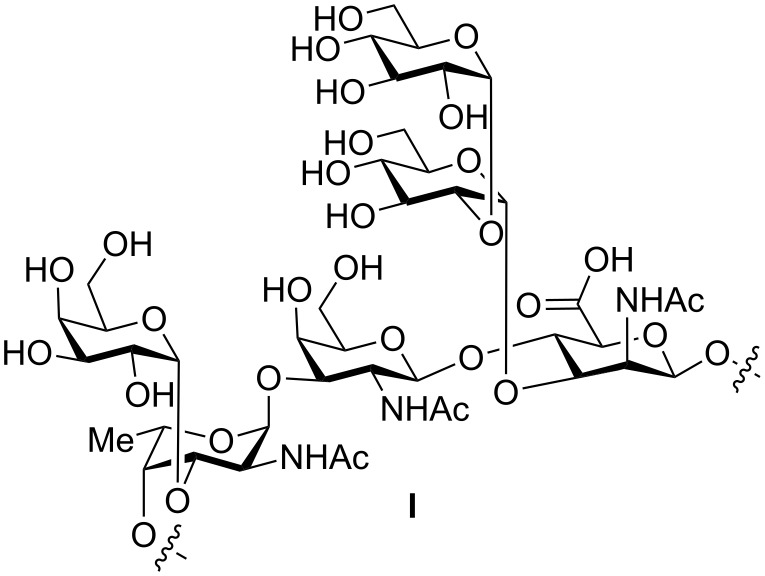
Structure of the *S. pneumoniae* serotype 12F capsular polysaccharide repeating unit [15].

*Streptococcus pneumoniae* is a Gram-positive bacterium that colonizes the upper respiratory tract and causes life-threatening pulmonary diseases as well as infections of the brain, the middle ear and the sinuses [1–6]. Twenty-three of the more than ninety *S. pneumoniae* serotypes, which differ in the capsular polysaccharides (CPS) that surround them, are responsible for about 90% of infections worldwide [7]. The licensed polysaccharide vaccine Pneumovax 23 contains serotype 12F but is not efficacious in young children or elderly people, those at highest risk. The carbohydrate conjugate vaccines Prevanar13™ and Synflorix™ [8–11] are based on CPS-carrier protein constructs and contain thirteen or ten *S. pneumoniae* serotypes, respectively, but not 12F [12–13]. The *S. pneumoniae* serotypes 12A [14] and 12F [15] combined account for more than 4% of pneumococcal disease [16], whereby 12F (Figure 1) dominates with 85% [17]. In order to improve current glycoconjugate vaccines additional serotypes such as 12F should be included in next-generation preparations [18].

Synthetic oligosaccharides are important tools for the identification of vaccine epitopes and have been the key to the creation of monoclonal antibodies that serve as tools for vaccine design [19] and for the detection of pathogenic bacteria such as *Bacillus anthracis* [20–21]. *S. pneumoniae* 12F CPS consists of hexasaccharide repeating units containing the [→4)-α-L-Fuc*p*NAc-(1→3)-β-D-Gal*p*NAc-(1→4)-β-D-Man*p*NAcA-(1→] polysaccharide backbone with a disaccharide branch at C3 of β-D-Man*p*NAcA and C3 of α-L-Fuc*p*NAc [15]. We established a total synthesis of the hexasaccharide repeat unit as a first step toward a detailed immunological analysis of *S. pneumoniae* 12F.

## Results and Discussion

**Scheme 1:**
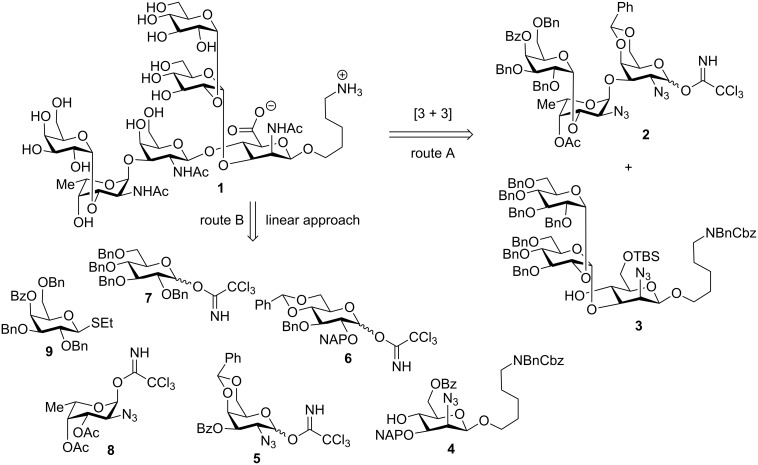
Retrosynthetic analyses of the *S. pneumoniae* hexasaccharide **1**.

**Retrosynthetic analysis.** Initially, a convergent [3 + 3] synthesis of the repeating unit hexasaccharide **1** was envisioned. The union of trisaccharides **2** and **3** (Scheme 1, route A) was identified as the key step. The outcome of this late-stage block coupling was deemed risky considering the poor nucleophilicity of the C4 hydroxy group of the β-mannosazide in **3** combined with steric bulk around the acceptor. Trisaccharides **2** and **3** can be derived from differentially protected common building blocks that carry *tert*-butyldimethylsilyl (TBS), benzoate (Bz) or acetate (Ac) ester and 2-naphthylmethyl (NAP) protecting groups that can be removed sequentially to allow for glycosylation of the liberated hydroxy groups. Formation of the β-mannosazide glycoside containing a protected C_5_ amino linker that serves in the final product as an attachment point for glycan array surfaces or carrier proteins was central to the assembly of trisaccharide **3**. To avoid a challenging and often unselective β-mannoside formation step we resorted to glucose–mannose conversion by inversion of the C2 stereocenter following selective installation of a *trans*-glucosidic linkage. Differentially protected thioglucoside **11** [22] is equipped with a participating C2 levulinyl ester that is replaced by an axial azido group following β-glucoside formation [23].

Alternatively, a linear synthetic strategy in which the sterically hindered C4 hydroxy group would be glycosylated first, followed by the C3 hydroxy group of β-mannosazide building block **4**, was designed in case the convergent approach proved unsuccessful (Scheme 1, route B).

**Scheme 2:**
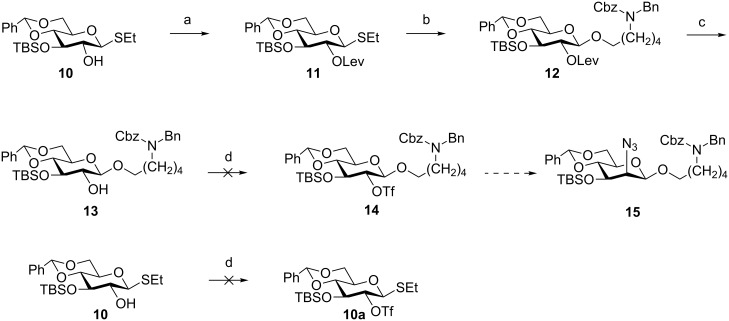
Attempted synthesis of mannosazide building block **15**. Reagents and conditions: (a) levulinic acid, DCC, DMAP, CH_2_Cl_2_, 82%; (b) NIS, TfOH, HO(CH_2_)_5_NBnCbz, CH_2_Cl_2_, −20 °C, 70%; (c) N_2_H_4_, AcOH, pyridine, CH_2_Cl_2_, 70%; (d) Tf_2_O, pyridine, CH_2_Cl_2_, 0 °C to rt.

**Building block synthesis.** The accessibility of differentially protected monosaccharide building blocks is a prerequisite for the successful total synthesis of any complex glycan. The synthesis of the mannosazide building block was the first challenge to be addressed. Installation of a C2-participating levulinyl ester protecting group ensured selective formation of the *trans*-glycoside upon activation of **11** by NIS/TfOH in the presence of the C_5_ linker to produce glucoside **12** in 70% yield [24]. Cleavage of the C2 levulinyl ester of **12** by treatment with hydrazine acetate furnished **13**, which was to be carried forward into the C2 inversion step. Conversion of **13** to the corresponding C2 triflate upon treatment with triflic anhydride in pyridine was not successful. Even model thioglycoside **10** failed to react to the corresponding glycosyl triflate under similar conditions (Scheme 2).

**Scheme 3:**
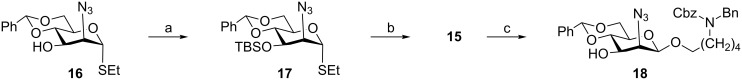
Synthesis of mannosazide building block **18**. Reagents and conditions: (a) TBSCl, imidazole, DCM, 0 °C to rt, 85%; (b) NIS, TfOH, HO(CH_2_)_5_NBnCbz, CH_2_Cl_2_, −20 °C, 61%; (c) TBAF, THF, 0 °C, 80%.

The problems associated with the lengthy and low yielding synthetic sequence prompted us to explore a different approach to obtain the key mannosazide building block (Scheme 3). Partially protected mannosazide thioglycoside **16** was prepared in seven steps from α-*O*-methylglucose following a published procedure [25]. Silylation of the C3 hydroxy group furnished thioglycoside **17**. Glycosylation of the C_5_ linker by activation of **17** using NIS/TfOH as the promoter at −20 °C produced mainly β-mannoside **15** (4:1 β:α) [26]. The identity of the β-isomer was confirmed by NMR analysis (^1^*J*_CH_ β = 159.0 Hz, see Supporting Information File 1). Cleavage of the silyl ether by TBAF treatment of **15** afforded the β-mannosazide building block **18**.

**Scheme 4:**
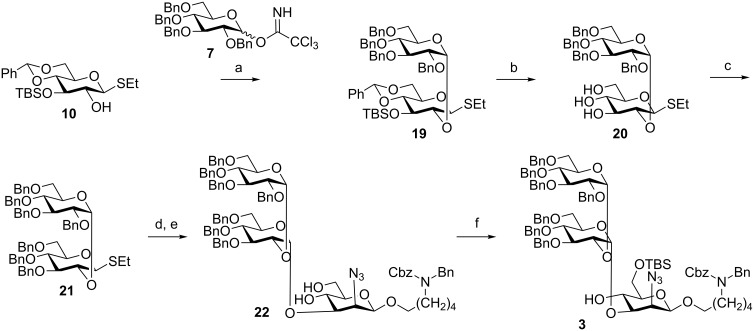
Synthesis of the reducing-end trisaccharide **3**. Reagents and conditions: (a) TMSOTf, (CH_3_CH_2_)_2_O/CH_2_Cl_2_ (4:1), −20 °C, α/β = 4:1, 70%; (b) *p*-TsOH, CH_3_OH/CH_2_Cl_2_ (1:1), rt, 70%; (c) NaH, benzyl bromide, THF/DMF (1:1), 0 °C to rt, 90%; (d) **18**, NIS, TfOH, (CH_3_CH_2_)_2_O/CH_2_Cl_2_, (4:1), −20 °C, 61%; (e) *p*-TsOH, CH_3_OH, rt, 90%; (f) TBSCl, imidazole, CH_2_Cl_2_, rt, 93%.

**Convergent [3 + 3] synthesis.** Synthesis of the reducing-end trisaccharide **3** (Scheme 1) commenced with the assembly of the α-1→2 linked diglucoside **19** by union of the monosaccharide building blocks **10** and **7** [27] in a dichloromethane–ether (enables alpha selectivity) mixture in 56% yield (Scheme 4). Removal of the silyl ether and benzylidene groups of **19** yielded triol **20** before benzylation afforded disaccharide thioglycoside building block **21**. Activation of disaccharide **21** resulted in the glycosylation of mannosazide acceptor **18** (Scheme 3) to form the corresponding α-linked trisaccharide, which, subsequent to removal of the 4,6-benzylidene group under acidic conditions, provided diol **22** that was in turn converted into reducing-end trisaccharide **3** by selective placement of a TBS ether [28] on the primary alcohol (Scheme 4).

**Scheme 5:**
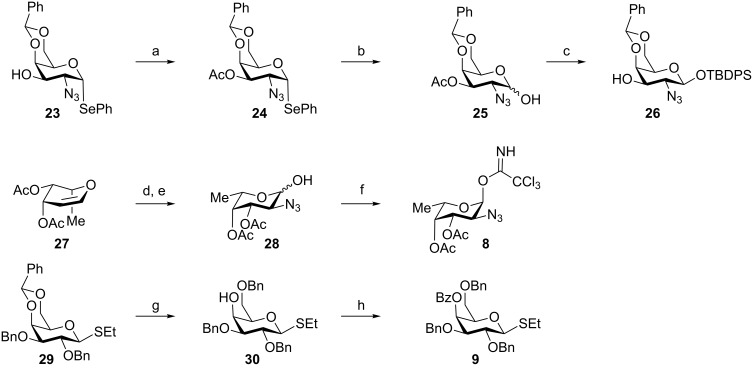
Synthesis of monosaccharide building blocks **8**, **9** and **26**. Reagents and conditions: (a) acetic anhydride, pyridine, CH_2_Cl_2_, rt, 18 h, 82%; (b) NIS, THF/H_2_O (1:1), rt; (c) 1) TBDPSCl, imidazole, DMF, rt; 2) NaOMe, MOH, rt, 68% over two steps; (d) (PhSe)_2_, BAIB, NaN_3_, CH_2_Cl_2_, rt, 24 h; (e) NIS, THF/H_2_O (1:1), rt, 80% over two steps; (f) CCl_3_CN, DBU, CH_2_Cl_2_, 0 °C to rt, 2 h, 72%; (g) TES, TFA, CH_2_Cl_2_, 0 °C, 6 h; (h) benzoyl chloride, pyridine, rt, 18 h, 80% over two steps.

With reducing-end trisaccharide **3** in hand, we turned our attention to the synthesis of trisaccharide **2**, which required the availability of three differentially protected monosaccharide building blocks: **8**, **9** and **26** (Scheme 5). Protected building block **26** was obtained in four steps from known galactosylazide selenide **23** [29]. Acetylation of the C3 hydroxy group of **23** furnished fully differentially protected selenoglycoside **24** in 82% yield. Hydrolysis of the selenoglycoside using NIS in aqueous THF produced hemiacetal **25** that was silylated prior to selective saponification of the C3 *O*-acetate to yield building block **26** (Scheme 5).

Fucosazide building block **8** was derived from diacetyl fucal **27** that in turn was prepared in two steps from L-fucose [30]. Azido-selenation of **27** and hydrolysis of the seleno fucosazide with NIS in aqueos THF [31] provided hemiacetal **28**, which was subsequently converted to the fucosyl trichloroacetimidate building block **8** (Scheme 5). Galactosyl thioglycoside **9** was prepared from D-galactose following published procedures [32]. Reductive opening of the benzylidene acetal of known galactosyl thioglycoside **29** [33] with triethylsilane [34] in TFA/CH_2_Cl_2_ liberated the C4 hydroxy group of **30**, which was subsequently benzoylated to ensure remote participation in **9** for the preferential formation of *cis*-glycosides (Scheme 5) [35].

**Scheme 6:**
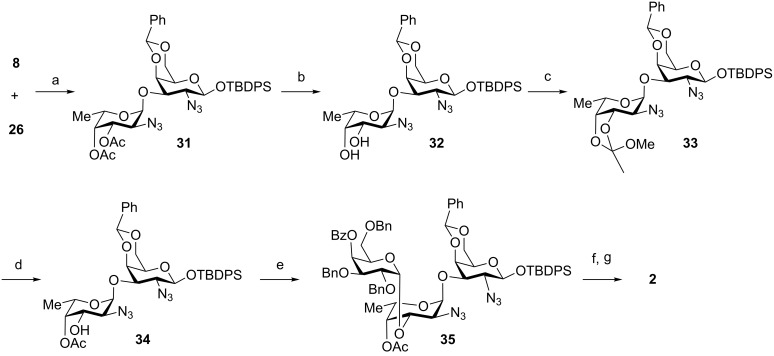
Synthesis of the non-reducing end trisaccharide **2**. Reagents and conditions: (a) TMSOTf, CH_2_Cl_2_, −30 °C, 74%; (b) NaOMe (0.5 M in MeOH), MeOH, rt; (c) trimethyl orthoacetate, *p*-TsOH, toluene; (d) 80% AcOH, rt, 71% over three steps; (e) **9**, NIS, TMSOTf, dioxane/toluene (3:1), −10 °C, 54%; (f) HF-pyridine, THF, 0 °C; (g) CCl_3_CN, DBU, CH_2_Cl_2_, 0 °C, 57% over two steps.

With the three building blocks **8**, **9** and **26** in hand, the assembly of the non-reducing end trisaccharide **2** commenced. The union of **8** and **26** produced the α-linked disaccharide **31** in 74% yield and excellent α-selectivity due to remote participation of the C2 and C3 acetate esters present in the fucosazide donor (Scheme 6). Preparation of the site for the downstream glycosylation, the C3 hydroxy group in the fucosazide moiety, to obtain **35**, required several protecting group manipulation steps: cleavage of the two acetate esters of **31** to produce diol **32** was followed by the reaction with trimethyl orthoacetate to provide the ortho-ester **33**, which was regioselectively opened under acidic conditions to afford disaccharide acceptor **34** containing a C3 hydroxy group [28]. Glycosylation of disaccharide **34** using galactose building block **9**, activated by NIS/triflic acid, produced trisaccharide **35** with high α-selectivity by virtue of the C4-participating benzoyl ester protecting group of **9** [36]. Trisaccharide **35** was transformed into a glycosylating agent by removal of the anomeric TBDPS silyl ether using HF-pyridine and subsequent treatment with trichloroacetonitrile in the presence of catalytic amounts of DBU to afford glycosyl trichloroacetimidate trisaccharide **2** (Scheme 1).

**Scheme 7:**
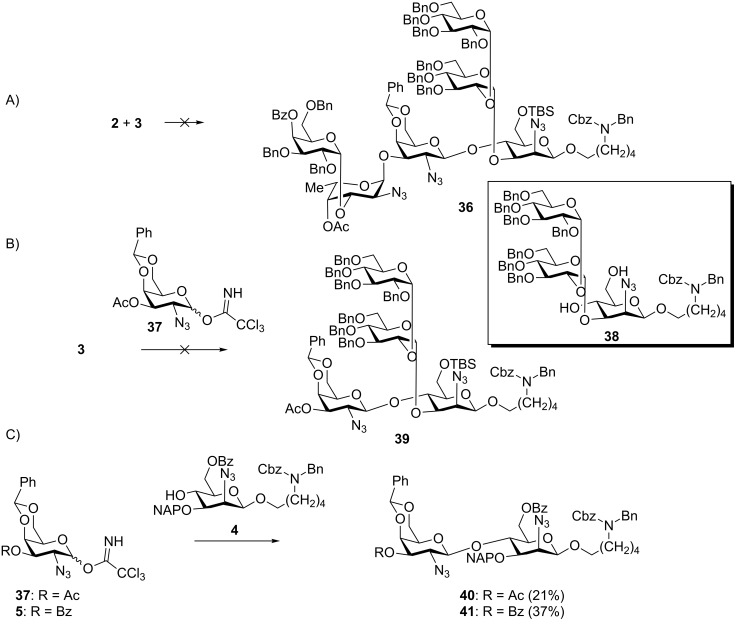
Attempted synthesis of hexasaccharide repeating unit **36** via a convergent [3 + 3] glycosylation strategy and exploratory control experiments. A) Failed [3 + 3] glycosylation; B) failed model [1 + 3] glycosylation; C) low yielding model coupling of two monosaccharides.

With trisaccharide fragments **2** and **3** in hand, the convergent [3 + 3] approach (Scheme 1, route A) to the synthesis of the repeating unit hexasaccharide **36** (Scheme 7) was attempted. The union of trisaccharides **2** and **3** using TMSOTf in acetonitrile as the activator did not yield the desired hexasaccharide **36**. Instead, trisaccharide acceptor **3** missing its C6 silyl ether protecting group **38** was recovered. The undesired outcome of this coupling step likely resulted from the poor nucleophilicity of **3** rather than a lack of reactivity of trisaccharide glycosylating agent **2**, as demonstrated by an experiment in which model monosaccharide **37** [29] also failed to react with trisaccharide acceptor **3** and recovered **38** (Scheme 7).

In order to better understand the formation of the key disaccharide GalNAc→ManNAcA, a model glycosylation involving mannosazide **4** (Scheme 7, prepared in four steps from **16**, see Supporting Information File 1) was explored. Differentially protected mannosazide **4** was successfully glycosylated using building blocks **5** or **37** to yield the corresponding disaccharide in 21% and 37% yield, respectively. The failure of the [3 + 3] coupling to produce hexasaccharide **36** was apparently a result of the poor nucleophilicity of the C4 hydroxy group in **3** rather than of problems associated with the glycosylating agents. The presence of a disaccharide appendage at the C3 position as well as a bulky TBS silyl ether at C6 may block the C4 hydroxy group.

The β-selectivity of glycosylations using glycosylating agents **5** and **37** even in acetonitrile was rather poor. Apparently, the “nitrile effect” [37–38] is partially overruled by the participating nature of the C3 ester protecting groups Ac/Bz that leads to a preference for the *cis*-glycosidic α-linked product.

**Scheme 8:**
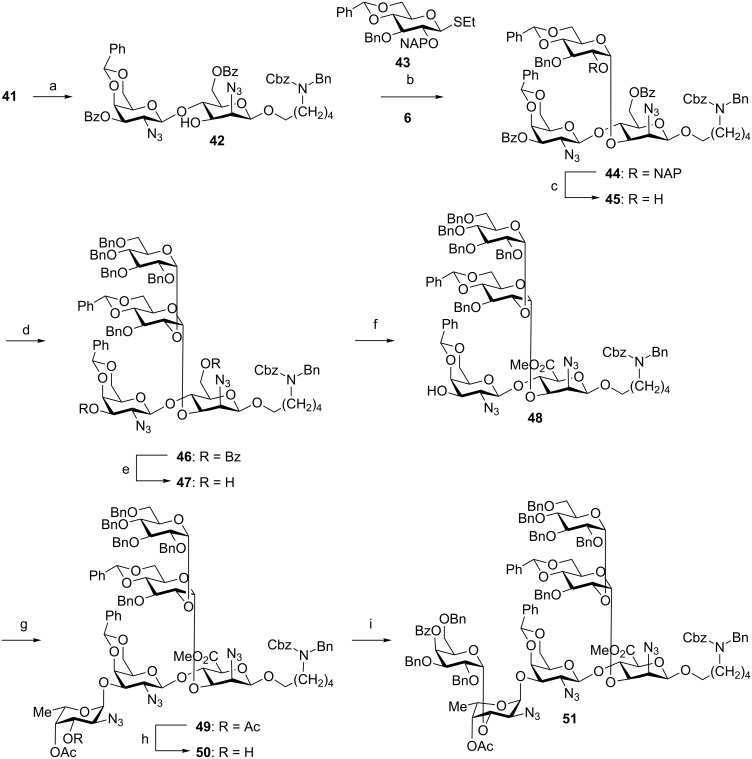
Linear assembly of fully protected hexasaccharide **51**. Reagents and conditions: (a) DDQ, CH_2_Cl_2_/MeOH (9:1), rt, 70%; (b) **43**, NIS, TfOH, CH_2_Cl_2_, −20 °C (no reaction) or **6**, TMSOTf, Et_2_O/CH_2_Cl_2_ (4:1), −20 °C (65%); (c) DDQ, CH_2_Cl_2_/MeOH (9:1), rt, 55%; (d) **7**, TMSOTf, Et_2_O/CH_2_Cl_2_ (4:1), −20 °C, 62%; (e) NaOMe (0.5 M in MeOH), THF/MeOH (1:1), rt, 90%; (f) i. TEMPO, BAIB, CH_2_Cl_2_/H_2_O (4:1), rt; ii. MeI, K_2_CO_3_, DMF, rt, 35% over two steps; (g) **8**, TMSOTf, CH_2_Cl_2_, −20 °C, 77%; (h) i. NaOMe (0.5 M in MeOH), THF/MeOH (1:1); ii. trimethyl orthoacetate, *p*-TsOH, toluene; iii. 80% AcOH, rt, 27% over three steps; (i) **9**, NIS, TMSOTf, dioxane/toluene (3:1), −10 °C to 0 °C, 54%.

**Linear total synthesis of 12F repeating unit hexasaccharide 1.** The failure of the convergent [3 + 3] total synthesis approach prompted us to retreat to the linear avenue (Scheme 1, route B) towards the target oligosaccharide in order to avoid the sterically demanding late-stage glycosylation. Differentially protected mannosazide **4** served as the starting point for stepwise assembly from the reducing to the non-reducing end (Scheme 8). Union of **4** and **5** (Scheme 7) produced disaccharide **41** as the key intermediate, the naphthyl protecting group of which was cleaved in 70% yield using DDQ [37] to afford **42**. Thioglycoside **43** failed to react with disaccharide **42** to furnish the desired trisaccharide **44**. Considering a potential “mismatch” [38] between the thioglycoside glycosylating agent and the acceptor [39–40] we explored whether the glucosyl trichloroacetimidate donor **6** (Scheme 1) would be more suitable. Indeed, glycosylation of disaccharide **42** with building block **6** using TMSOTf as the activator proceeded to produce trisaccharide **44** in 65% yield.

Removal of the C2 naphthyl ether using DDQ provided acceptor **45**, which in turn was reacted with glucosyl thioglycoside **7** in the presence of NIS and TfOH to produce α-linked tetrasaccharide **46** in 62% yield (Scheme 8). At this stage, the 2-azidomannose moiety of **46** was converted to the corresponding mannosaminuronic acid by cleaving the C6 benzoate ester using sodium methoxide in methanol and selective oxidation of the primary alcohol of **47** using BAIB/TEMPO. Tetrasaccharide acceptor **48** was obtained by esterification of the carboxylic acid under basic conditions using methyl iodide in 32% yield over three steps [41]. Next, TMSOTf activation of fucosyl trichloroacetimidate **8** (Scheme 1) catalyzed the glycosylation of methyl uronate **48** to afford pentasaccharide **49** exclusively as the α-isomer by virtue of remote participation of the 3-*O*-acetate group. In anticipation of the final glycosylation, the fucosazide moiety of **49** was converted into acceptor **50**. The desired hexasaccharide **51** was obtained as the α-anomer in 54% yield by coupling galactose building block **9** (Scheme 1) to pentasaccharide **50** using NIS/TfOH in a mixture of toluene/dioxane. Again, the C4 benzoate ester of **9** ensured high selectivity for the desired *cis*-glycosidic linkage.

**Scheme 9:**
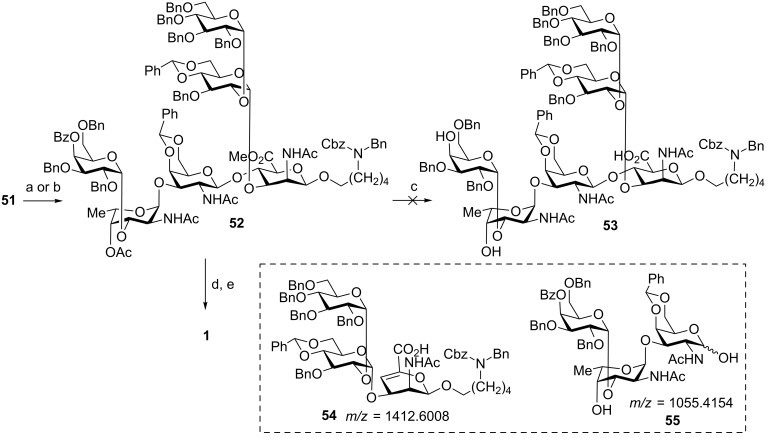
Global deprotection to furnish *S. pneumonia* serotype 12F repeating unit hexasaccharide **1**. Reagents and conditions: (a) thioacetic acid, pyridine, rt, 65%; (b) Zn, AcOH/Ac_2_O/THF, Cu_2_SO_4_ (aq, decomposed); saponification conditions that lead to β-elimination (c) i. NaOMe (0.5 M in MeOH) in MeOH, ii. NaOH (4 M, 2 M, 1 M, 0.5 M, 0.1 M) solution in THF, iii. H_2_O_2,_ LiOH, THF, H_2_O; successful sequence (d) Pd/C, AcOH, H_2_O, *t*-BuOH (e) H_2_O_2,_ LiOH, THF, H_2_O, 37% over two steps.

Global deprotection commenced with the conversion of the three azide groups present in compound **51** into NHAc groups in a single step using thioacetic acid in pyridine [42] afforded triacetamide **52** in 65% yield; it is important to note that Zn-mediated reduction of **51** led to decomposition of the substrate. Ester saponification of **52** employing sodium methoxide in methanol yielded none of the desired product **53** but rather the tentatively assigned β-elimination products **54** and **55**. Furthermore, an attempt at employing a more nucleophilic and less basic reagent such as a lithium hydroxide/hydrogen peroxide mixture did not provide relief from the problem, but instead also produced a mixture of undesired products. Adjustments in the sequence of deprotection steps by first carrying out hydrogenolysis using Pd/C in a mixture of AcOH/H_2_O/*t*-BuOH prior to ester hydrolysis using LiOH/H_2_O_2_ enabled the hexasaccharide **1** to be obtained in 37% yield (Scheme 9).

## Conclusion

The first total synthesis of the *S. pneumoniae* serotype 12F capsular polysaccharide repeating unit hexasaccharide **1** was achieved by means of a linear approach. A convergent [3 + 3] total synthesis strategy failed, most likely due to steric crowding around the trisaccharide acceptor. The synthesis of **1** is an illustrative example of the challenges associated with state-of-the-art oligosaccharide assembly including steric, conformational, remote participation groups and solvent effects. It lends further credence to the linear assembly concept in which one monosaccharide unit at a time is incorporated, and which serves as the basis for automated glycan assembly [43]. With the synthetically sourced hexasaccharide repeating unit in hand detailed immunological analysis of *S. pneumoniae* serotype 12F can be undertaken, and future work will address the expanded inclusion of this antigen in next-generation glycoconjugate vaccines.

## Abbreviations

Ac: acetate ester; BAIB: bis(acetoxy)iodobenzene; Bz: benzoyl; CPS: capsular polysaccharide; DMF: *N,N*-dimethylformamide; EtOAc: ethyl acetate; GlcA: glucouronic acid; HPLC: high-performance liquid chromatography; Lev: levulinoyl; MALDI–TOF MS: matrix-assisted laser desorption/ionization–time of flight mass spectrometry; NAP: 2-naphthylmethyl; TBS: *tert*-butyldimethylsilyl; THF: tetrahydrofuran; TMSOTf: trimethylsilyl trifluoromethanesulfonate.

## Supporting Information

File 1 Experimental details and full characterization data of all new compounds.
