# Supplementary material for: Total synthesis of a Streptococcus pneumoniae serotype 12F CPS repeating unit hexasaccharide
Source: Beilstein J Org Chem. 2017 Jan 25;13:164–73. doi: 10.3762/bjoc.13.19 (PMC5301915; doi:10.3762/bjoc.13.19)
Supplement: File 1 — Experimental details and full characterization data of all new compounds. [file Beilstein_J_Org_Chem-13-164-s001.pdf]

Supporting Information

for

Total synthesis of a *Streptococcus pneumoniae* serotype 12F CPS repeating unit hexasaccharide

Peter H. Seeberger[1,2,]*, Claney L. Pereira[3] and Subramanian Govindan[1]

Address: [1]Department of Biomolecular Systems, Max Planck Institute of Colloids and Interfaces, Am Mühlenberg 1, 14476 Potsdam, Germany, [2]Department of Chemistry and Biochemistry, Freie Universität Berlin, Arnimallee 22, 14195 Berlin, Germany and [3]Vaxxilon Deutschland GmbH, Magnusstrasse 11, 12489 Berlin, Germany

*Corresponding author

Email: Peter H. Seeberger - peter.seeberger@mpikg.mpg.de

Experimental details and full characterization data of all new compounds

Contents

	page
General information	S2
Experimental details and characterization data of new compounds	S3
NMR spectra of new compounds	S37
References	S76

General information

Commercial grade solvents and reagents were used without further purification, unless otherwise indicated. All reactions were performed using oven dried glassware under an Argon atmosphere. Analytical thin layer chromatography (TLC) was performed on Kieselgel 60 F254 glass plates precoated with a 0.25 mm thickness of silica gel. The TLC plates were visualized with UV light or by staining with Hanessian solution (ceric sulfate and ammonium molybdate in aqueous sulfuric acid, CAM stain) or a 1:1 mixture of H_2SO_4 (2N) and resorcine monomethylether (0.2%) in ethanol. Column chromatography was performed using silica gel 60 (230–400 mesh). Size exclusion chromatography (SEC) was performed using Sephadex ® LH-20 (GE Healthcare).

^{1}H NMR and ^{13}C NMR spectra were measured with a Varian 400-MR or Varian 600 spectrometer. For internal reference of ^{1}H spectra, the proton signal of residual, non-deuterated solvent (δ 7.26 ppm for $CHCl_3$; δ 4.79 ppm for H_2O) was used. For ^{13}C spectra, the chemical shifts are reported relative to the respective solvent (δ 77.0 ppm for $CDCl_3$). Coupling constants are reported in Hertz (Hz). Multiplicities are listed as: s, singlet; d, doublet; t, triplet; m, multiplet; br s, broad singlet; br d, broad doublet; br m, broad multiplet. Infrared (IR) spectra were measured as thin films on a Perkin Elmer Spectrum 100 FTIR spectrophotometer. Optical rotations (OR) were obtained with a Schmidt and Haensch UniPol L 1000 at 589 nm and a concentration (c) expressed in g/100 mL. LC-MS were recorded with an Agilent 1100 series instrument, and high-resolution mass spectra (HRMS) were recorded using an Agilent 6210 ESI-TOF mass spectrometer at the Freie Universität Berlin, Mass Spectrometry Core Facility. MALDI-TOF spectra were measured on a Bruker Daltonics Autoflex Speed, using a 2,4,6-trihydroxyacetophenone (THAP) matrix.

Experimental details and characterization data of new compounds

Ethyl 4,6-*O*-benzylidene-3-*O*-*tert*-butyldimethylsilyl-2-*O*-levulinoyl-1-thio-β-D-glucopyranoside (11)

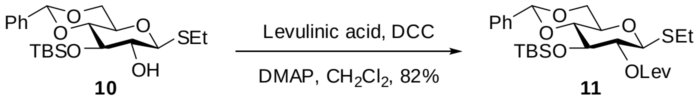

To a solution of **10**[1] (1.50 g, 3.515 mmol, 1.0 equiv) in CH_2Cl_2 (18 mL) at room temperature, was added levulinic acid (0.61 g, 5.273 mmol, 1.5 equiv), DCC (1.09 g, 5.273 mmol, 1.5 equiv) and DMAP (0.64 g, 5.273 mmol, 1.5 equiv). After stirring for 20 h, the reaction mixture was quenched by the addition of aq. $NaHCO_3$, extracted with CH_2Cl_2 (30 mL) and concentrated in vacuo. The resulting yellow oil was purified by flash column chromatography over silica gel (EtOAc/hexanes, 2/8, v/v) to give **11** as (1.40 g, 82%) as a pale yellow oil: R_f = 0.4 (EtOAc/hexanes, 1/9, v/v); IR (thin film, cm^{-1}): ν_{max} = 669, 698, 759, 779, 814, 837, 861, 880, 915, 935, 1004, 1087, 1151, 1181, 1249, 1313, 1361, 1378, 1408, 1457, 1472, 1720, 1747, 2857, 2929; ^{1}H NMR (600 MHz, $CDCl_3$) δ 7.48 – 7.42 (m, 2H), 7.36 – 7.30 (m, 3H), 5.49 (s, 1H), 4.96 (dd, *J* = 10.1, 8.6 Hz, 1H), 4.43 (d, *J* = 10.1 Hz, 1H), 4.32 (dd, *J* = 10.5, 4.9 Hz, 1H), 3.87 (t, *J* = 8.7 Hz, 1H), 3.72 (t, *J* = 10.2 Hz, 1H), 3.51 (t, *J* = 9.2 Hz, 1H), 3.45 (td, *J* = 9.7, 4.9 Hz, 1H), 2.85 – 2.58 (m, 6H), 2.17 (s, 3H), 1.23 (t, *J* = 7.5 Hz, 3H), 0.79 (s, 9H), 0.01 (s, 3H), –0.03 (s, 3H); ^{13}C NMR (151 MHz, $CDCl_3$) δ 205.8, 171.3, 136.9, 128.9, 127.9, 126.1, 101.7, 84.0, 81.2, 73.8, 72.9, 70.5, 68.4, 37.8, 29.7, 28.1, 25.4, 25.4, 25.4, 23.8, 17.8, 14.6, -4.3, -5.0; HRMS: m/z: calcd. for $C_{26}H_{40}O_7SSi$ $[M+Na]^+$: 547.2162; found: 547.2143.

N-Benzyl-N-benzyloxycarbonyl-5-aminopentyl 4,6-O-benzylidene-3-O-*tert*-butyldimethylsilyl-2-O-levulinoyl-β-D-glucopyranoside (12)

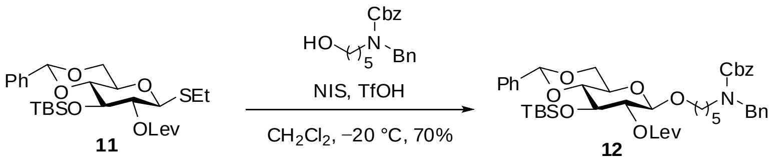

A mixture of glucosylating agent **11** (1.20 g, 2.290 mmol, 1.5 equiv) and N-benzyl-N-benzyloxycarbonyl-5-aminopentanol[2] (0.50 g, 1.527 mmol, 1.0 equiv) in CH_2Cl_2 (30 mL) was stirred under an atmosphere of Ar for 30 min. The reaction mixture was cooled (−20 °C) and NIS (0.515 g, 2.290 mmol, 1.5 equiv) was added followed by the addition of TfOH (68 μL, 0.764 mmol. 0.5 equiv). After stirring for 45 min, the reaction mixture was quenched by the addition of Et_3N (1.0 mL). The organic solution was diluted with CH_2Cl_2 (30 mL) and the mixture was washed with 10% $Na_2S_2O_3$ (50 mL) and brine (100 mL). The organic phase was dried over anhydrous Na_2SO_4, filtered and the filtrate was concentrated in vacuo. The resulting dark yellow oil was purified by flash column chromatography over silica gel (EtOAc/hexanes, 1/3, v/v) to give **12** (0.520 g, 70%) as a pale yellow oil: R_f = 0.5 (EtOAc/hexanes, 4/6, v/v); $[\alpha]_D^{20}$ = –44.4° (c = 1.0, $CHCl_3$); IR (thin film, cm^{-1}): ν_{max} = 670, 698, 765, 779, 838, 914, 1005, 1029, 1097, 1132, 1149, 1173, 1212, 1249, 1304 ,1361, 1420, 1455, 1472, 1496, 1698, 1749, 2857, 2929, 3033; ^{1}H NMR (600 MHz, $CDCl_3$) δ 7.50 – 7.45 (m, 2H), 7.40 – 7.14 (m, 13H), 5.50 (s, 1H), 5.18 (m, 2H), 4.92 (t, J = 8.5 Hz, 1H), 4.51 (d, J = 11.4 Hz, 2H), 4.45 – 4.27 (m, 2H), 3.86 (t, J = 9.0 Hz, 1H), 3.76 (t, J = 10.2 Hz, 2H), 3.51 (t, J = 9.2 Hz, 1H), 3.46 – 3.37 (m, 2H), 3.29 – 3.19 (m, 2H), 2.77 2.56 (m, 4H), 2.14 (d, J = 10.2 Hz, 3H), 1.59 – 1.49 (m, 4H), 1.30 – 1.24 (m, 2H), 0.81 (s, 9H), 0.02 (s, 3H), –0.01 (s, 3H); ^{13}C NMR (151 MHz, $CDCl_3$) δ 205.9, 171.1, 156.6, 156.0, 137.0, 128.9, 128.4, 128.0, 127.8, 127.7, 127.1, 126.1, 101.7, 101.5, 81.4, 74.6, 72.6, 69.8, 68.6, 67.0, 66.2, 50.4, 50.1, 47.0, 46.1, 37.7, 29.8, 29.1, 27.9, 27.8, 27.3, 25.5, 23.0, 17.9, -4.2, -4.9; HRMS: m/z: calcd. for $C_{44}H_{59}NO_{10}Si$ $[M+Na]^+$: 812.3806; found: 812.3786.

N-Benzyl-N-benzyloxycarbonyl-5-aminopentyl 4,6-O-benzylidene-3-O-$tert$-butyldimethylsilyl-β-D-glucopyranoside (13)

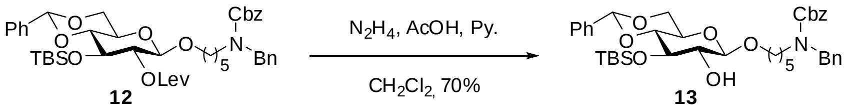

To a solution of **12**[3] (100 mg, 0.139 mmol, 1.0 equiv) in ethanol (2.5 mL) containing AcOH (0.3 mL) and pyridine (0.45 mL) at room temperature, was added hydrazine acetate (64.3 mg, 0.698 mmol, 5.0 equiv). After stirring for 3 h, the reaction mixture was concentrated in vacuo. The residue was purified by flash column chromatography over silica gel (EtOAc/hexanes, 1/4, v/v) to give **13** as (60 mg, 70%) as a pale yellow oil: R_f = 0.6 (EtOAc/hexanes, 4/6, v/v); ^{1}H NMR (400 MHz, $CDCl_3$) δ 7.38 – 7.17 (m, 15H), 5.45 (s, 1H, benzylidene CH), 5.13 (d, J = 11.5 Hz, 2H), 4.49 – 4.41 (m, 2H), 4.32 – 4.24 (m, 2H, C-1^I), 3.91 – 3.65 (m, 3H), 3.53 – 3.32 (m, 4H), 3.25 – 3.14 (m, 2H), 1.64 – 1.42 (m, 4H), 1.35 – 1.23 (m, 2H), 0.83 (s, 9H), 0.06 (s, 3H), 0.00 (s, 3H); ^{13}C NMR (101 MHz, $CDCl_3$) δ 156.8, 156.2 (Cbz NHCO), 137.8, 128.8, 128.4, 128.3, 128.0, 127.8 (2C), 126.1, 103.5 (C-1^I), 101.5 (benzylidene CH), 81.2, 75.4, 74.4, 68.6, 67.1, 66.4, 50.4, 50.2, 46.9, 46.0, 29.2, 29.0, 27.7, 27.1, 25.7, 23.3, 23.1, 18.2, -4.3, -4.7; LCMS: m/z: calcd. for $C_{39}H_{53}NO_8Si$ $[M+Na]^+$: 714.3; found: 714.3

Ethyl 2-azido-4,6-O-benzylidene-3-O-$tert$-butyldimethylsilyl-2-deoxy-β-D-glucopyranoside (17)

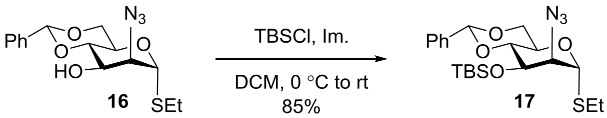

To a solution of **16**[4] (2.20 g, 6.520 mmol, 1.0 equiv) in CH_2Cl_2 (22 mL) was added imidazole (0.66 g, 9.780 mmol, 1.5 equiv) at room temperature. The solution was cooled to 0 °C, TBSCl (1.47 g, 1.081 mmol, 1.1 equiv) was added and the contents were warmed to room temperature over 18 h. The reaction mixture was quenched with aq. NH_4Cl (50 mL), extracted with CH_2Cl_2 (50 mL), washed with brine (50 mL), dried over anhydrous Na_2SO_4 and

concentrated in vacuo. The residue was purified by column chromatography (EtOAc/hexanes, 1/9, v/v) to give **17** (2.50 g, 85%) as a pale yellow oil. R_f = 0.7 (EtOAc/hexanes, 2/8, v/v); $[\alpha]_D^{20}$ = +197.6° (c = 1.0, $CHCl_3$); IR (thin film, cm^{-1}): ν_{max} = 697, 746, 780, 838, 854, 885, 907, 968, 1015, 1074, 1096, 1132, 1152, 1211, 1257, 1379, 1471, 2104, 2858, 2929; ^{1}H NMR (400 MHz, $CDCl_3$) δ 7.50 – 7.48 (m, 2H), 7.39 – 7.34 (m, 3H), 5.57 (s, 1H), 5.23 (d, *J* = 1.0 Hz, 1H), 4.28 (dd, *J* = 9.5, 3.8 Hz, 1H), 4.24 – 4.17 (m, 2H), 3.94 (dt, *J* = 8.5, 6.8 Hz, 2H), 3.87 – 3.78 (m, 1H), 2.75 – 2.53 (m, 2H), 1.30 (t, *J* = 7.4 Hz, 3H), 0.89 (s, 9H), 0.11 (s, 3H), 0.06 (s, 3H); ^{13}C NMR (101 MHz, $CDCl_3$) δ 137.3, 128.9, 128.0, 126.1, 102.0 (benzylidene CH), 83.6 (C-1^I), 79.4, 70.7, 68.4, 66.6, 64.4, 25.7, 25.4, 18.2, 14.8, –4.5, –4.9; HRMS: m/z: calcd. for $C_{21}H_{33}N_3O_4SSi$ $[M+Na]^+$: 474.1859; found: 474.1859.

N-Benzyl-N-benzyloxycarbonyl-5-aminopentyl 2-azido-4,6-O-benzylidene-2-deoxy-3-O-*tert*-butyldimethylsilyl-β-D-glucopyranoside (15)

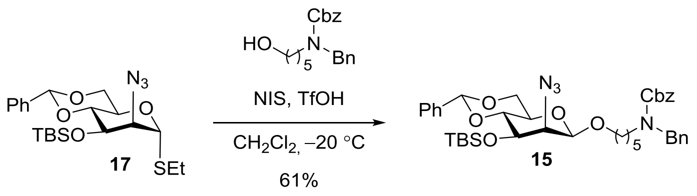

A mixture of mannosyl donor **17** (758 mg, 1.680 mmol, 1.1 equiv) and *N*-benzyl-*N*-benzyloxycarbonyl-5-aminopentanol[2] (500 mg, 1.527 mmol, 1.0 equiv) in CH_2Cl_2 (30 mL) was stirred under an atmosphere of Ar for 30 min. The reaction mixture was cooled (–20 °C) and NIS (378 mg, 1.680 mmol, 1.1 equiv) was added followed by the addition of TfOH (68 μL, 0.764 mmol, 0.5 equiv). After stirring for 45 min, the reaction mixture was quenched by the addition of Et_3N (1.0 mL). The organic solution was diluted with CH_2Cl_2 (30 mL) and the mixture was washed with 10% $Na_2S_2O_3$ (50 mL) and brine (100 mL). The organic phase was dried over anhydrous Na_2SO_4, filtered and the filtrate was concentrated in vacuo. The resulting dark yellow oil was purified by flash column chromatography over silica gel (EtOAc/hexanes, 1/3, v/v) to give **15** as the major product (β/α =4:1, *β*–isomer, 665 mg, 61%

and α–isomer, 167 mg, 15%) as a pale yellow oil: β–isomer: R_f = 0.7 (EtOAc/hexanes, 4/6, v/v). $[\alpha]_D^{20}$ = +16.4° (c = 2.6, $CHCl_3$); IR (thin film, cm^{-1}): ν_{max} = 670, 697, 750, 767, 781, 838, 858, 885, 914, 968, 1005, 1026, 1085, 1103, 1130, 1172, 1215, 1251, 1273, 1305, 1379, 1420, 1455, 1471,1496, 1698, 2105, 2858, 2929, 3034; ^{1}H NMR (400 MHz, $CDCl_3$) δ 7.64 – 7.41 (m, 2H), 7.44 – 7.13 (m, 13H), 5.54 (s, 1H), 5.19 (d, J = 8.1 Hz, 2H), 4.54 (br s, 3H, C-1^I), 4.29 (dd, J = 10.4, 4.9 Hz, 1H), 3.99 (dd, J = 9.3, 3.5 Hz, 1H), 3.93 – 3.75 (m, 4H), 3.46 (br m, 1H), 3.29 (br m, 3H), 1.57 (br m, 4H), 1.31 (br m, 2H), 0.92 (s, 9H), 0.12 (s, 3H), 0.08 (s, 3H); ^{13}C NMR (101 MHz, $CDCl_3$) δ 156.5, 156.1, 137.7, 137.4, 137.1, 128.8, 128.7, 128.4, 128.3, 127.9 (2C), 127.8, 127.7, 127.1, 126.1, 126.0, 101.7 (benzylidene CH), 99.8 ($J_{CH}\,\beta$ = 158 Hz, C-1^I), 78.5, 72.0, 69.8, 68.3, 67.3, 67.0, 66.0, 50.4, 49.8, 46.9, 46.0, 29.0, 27.7, 27.3, 25.6, 25.6, 23.0, 18.1, –4.4, –5.0; HRMS: m/z: calcd. for $C_{39}H_{52}N_4O_7Si$ $[M+Na]^+$: 739.3503; found: 739.3501.

***N*-Benzyl-*N*-benzyloxycarbonyl-5-aminopentyl 2-azido-4,6-*O*-benzylidene-2-deoxy-β-D-glucopyranoside (18)**

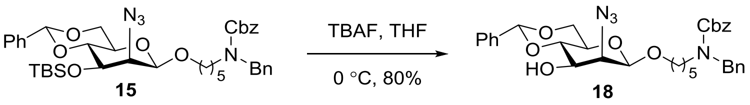

Monosaccharide **15** (500 mg, 0.697 mmol, 1.0 equiv) was dissolved in THF (20 mL) and the solution was cooled to 0 °C. TBAF (1.04 mL, 1.046 mmol, 1.5 equiv) was added and the contents were stirred for 4 h. The reaction mixture was quenched with water (25 mL), extracted with EtOAc (25 mL), washed with brine (50 mL), dried over anhydrous Na_2SO_4 and concentrated in vacuo. The residue was purified by column chromatography (EtOAc/hexanes, 1/1, v/v) to give **18** (350 mg, 80%) as a colorless oil. R_f = 0.4 (EtOAc/hexanes, 1/1, v/v); $[\alpha]_D^{20}$ = –1.6° (c = 2.0, $CHCl_3$); IR (thin film, cm^{-1}): ν_{max} = 698, 749, 821, 994, 1051, 1099,

1170, 1276, 1365, 1422, 1454, 1693, 2111, 2942; ^{1}H NMR (400 MHz, $CDCl_3$) δ 7.65 – 7.44 (m, 2H), 7.41–7.15 (m, 13H), 5.51 (s, 1H, benzylidene CH), 5.19 (d, *J* = 12.3 Hz, 2H), 4.51 (d, *J* = 7.1 Hz, 3H, C-1^I), 4.30 (dd, *J* = 10.4, 4.9 Hz, 1H), 3.90 (br m, 2H), 3.87 – 3.78 (m, 2H), 3.73 (t, *J* = 9.4 Hz, 1H), 3.56–3.35 (m, 1H), 3.32–3.19 (m, 3H,), 2.99 (br s, 1H), 1.67–1.49 (m, 4H,), 1.34 (br m, 2H,); ^{13}C NMR (101 MHz, $CDCl_3$) δ 156.5, 156.0, 137.7, 136.9, 129.5, 129.0, 128.8, 128.3, 128.2, 128.1, 128.0, 127.7, 127.6, 127.1, 126.1, 125.9, 101.8 (benzylidene CH), 100.3 (C-1^I), 78.3, 69.7, 68.1, 67.0, 66.8, 64.5, 53.3, 50.3, 50.0, 46.8, 46.0, 29.0, 27.6, 27.2, 22.9; HRMS: m/z: calcd. for $C_{33}H_{38}N_4O_7Si$ $[M+Na]^+$: 625.2638; found: 625.2646.

Ethyl *O*-(2,3,4,6-tetra-*O*-benzyl-α-D-glucopyranosyl)-(1→2)-1-thio-β-D-glucopyranoside (20)

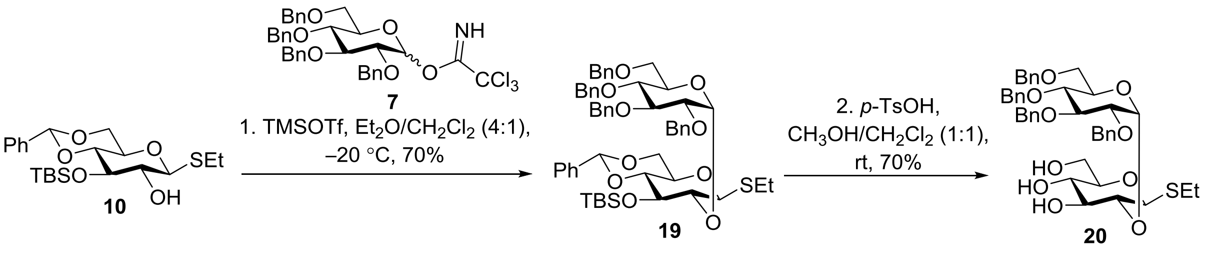

A mixture of glucosylating agent **7**[5] (1.00 g, 2.344 mmol, 1.0 equiv) and acceptor **10**[1] (1.394 g, 2.344 mmol, 1.0 equiv) in Et_2O/ CH_2Cl_2 (4:1, 40 mL) was stirred under an atmosphere of Ar for 30 min. The reaction mixture was cooled (−20 °C) and TMSOTf (212 µL, 1.172 mmol, 0.5 equiv) was added. After stirring for 45 min, the reaction mixture was quenched by the addition of Et_3N (1.0 mL). The mixture was concentrated in vacuo. The resulting dark yellow oil was purified by flash column chromatography over silica gel (EtOAc/hexanes, 1/5, v/v) to give a non-separable mixture of β and α isomers (β/α =1:4, 1.67 g, 70%) with **19** (major product) as a colorless oil: R_f = 0.7 (EtOAc/hexanes, 2/8, v/v); IR (thin film, cm^{-1}): ν_{max} = 697, 748, 781, 839, 913, 1027, 1071, 1091, 1210, 1251, 1313, 1361, 1454, 1496, 1727, 2857,

2928, 3031; ^{1}H NMR (400 MHz, $CDCl_3$) δ 7.95 – 6.83 (m, 25H), 6.14 (d, *J* = 3.7 Hz, 1H, C-1^{II}), 5.49 (s, 1H, benzylidene CH), 5.02 – 4.96 (m, 2H), 4.94 – 4.88 (m, 2H), 4.79 (d, *J* = 8.9 Hz, 1H, C-1^{I}), 4.74 – 4.61 (m, 2H), 4.63 – 4.52 (m, 1H), 4.46 – 4.39 (m, 2H), 4.27 – 4.19 (m, 2H), 4.01 (dd, *J* = 8.7, 8.0 Hz, 1H), 3.88 – 3.71 (m, 5H), 3.79 – 3.72 (m, 1H), 3.69 – 3.52 (m, 2H), 3.03–2.65 (m, 2H), 1.42–1.32 (m, 3H), 0.86 (s, 9H), 0.27 (s, 3H), 0.10 (s, 3H); ^{13}C NMR (101 MHz, $CDCl_3$) δ 138.6, 138.6, 137.9, 137.8, 136.8, 128.1 (3C), 128.0 (2C), 127.9, 127.8 (4C), 127.7, 127.5 (3C), 127.3, 126.8, 126.4, 102.4 (benzylidene CH), 94.7 (C-1^{II}), 84.3 (C-1^{I}), 81.4, 80.9, 79.2, 77.5, 75.3, 75.0, 74.7, 74.4, 73.2, 72.6, 69.8, 69.6, 68.6, 68.2, 25.9, 24.1, 18.1, 14.4, –2.2, –3.8; HRMS: m/z: calcd. for $C_{55}H_{68}O_{10}SSi$ $[M+Na]^+$: 971.4200; found: 971.4207. To a solution of **19** (715 mg, 0.753 mmol) in CH_2Cl_2/MeOH (1:1) (20 mL) at room temperature was added *p*-TsOH (14 mg, 0.075 mmol, 0.1 equiv). After stirring for 6 h, the reaction mixture was quenched by the addition of aq. $NaHCO_3$ (10 mL), extracted in EtOAc (50 mL), dried over anhydrous Na_2SO_4 and concentrated in vacuo. The resulting yellow oil was purified by flash column chromatography over silica gel (EtOAc/hexanes, 1/1, v/v) to give triol **20** (250 mg, 70%) as a colorless oil; R_f = 0.3 (EtOAc/hexanes, 1/1, v/v); $[\alpha]_D^{20}$ = +6.9° (c = 2.0, $CHCl_3$); IR (thin film, cm^{-1}): ν_{max} = 697, 748, 1026, 1069, 1262, 1270,1357, 1453, 1496, 2861, 2921, 3428; ^{1}H NMR (600 MHz, $CDCl_3$) δ 7.42 – 7.38 (m, 2H), 7.37 – 7.28 (m, 16H), 7.21 – 7.13 (m, 2H), 5.16 (d, *J* = 3.7 Hz, 1H, C-1^{II}), 4.97 (d, *J* = 10.8 Hz, 1H), 4.86 – 4.76 (m, 3H), 4.68 (d, *J* = 11.7 Hz, 1H), 4.53 (t, *J* = 8.8 Hz, 1H), 4.51 – 4.43 (m, 3H, C-1^{I}), 4.23 – 4.15 (m, 1H), 3.97 (t, *J* = 9.3 Hz, 1H), 3.88 (dd, *J* = 11.9, 3.5 Hz, 1H), 3.75 (dd, *J* = 11.9, 5.2 Hz, 1H), 3.70 (dd, *J* = 10.0, 1.9 Hz, 1H), 3.58 (ddd, *J* = 16.5, 9.8, 5.1 Hz, 2H), 3.48 (dd, *J* = 17.7, 8.9 Hz, 2H), 3.42 – 3.37 (m, 2H), 3.36 – 3.31 (m, 1H), 2.94 – 2.59 (m, 2H), 1.22 (t, *J* = 7.4 Hz, 3H); ^{13}C NMR (151 MHz, $CDCl_3$) δ 138.5, 137.9 (2C), 137.4, 128.4, 128.3 (4C), 128.1, 128.0, 127.9 (3C), 127.8, 127.7 (3C), 127.6, 97.2, 81.5, 79.7, 78.7, 77.9, 76.7, 75.6, 75.0, 73.6, 72.9, 70.8, 70.7, 69.1, 62.8, 25.0, 14.7; HRMS: m/z: calcd. for $C_{42}H_{50}O_{10}S$ $[M+Na]^+$: 769.3022; found: 769.3029.

Ethyl *O*-(2,3,4,6-tetra-*O*-benzyl-α-D-glucopyranosyl)-(1→2)-3,4,6-tri-*O*-benzyl-1-thio-β-D-glucopyranoside (21)

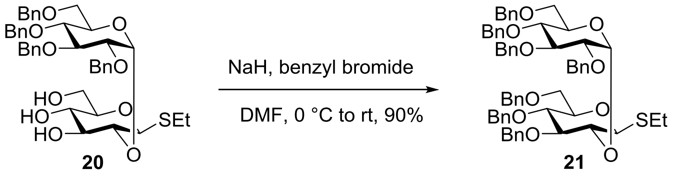

To a cooled (0 °C) solution of enantiomerically pure **20** (600 mg, 0.803 mmol, 1.0 equiv) and benzyl bromide (0.48 mL, 4.020 mmol, 5.0 equiv) in DMF (20 mL) under an atmosphere of Ar was added sodium hydride (129 mg, 3.210 mmol, 4.0 equiv, 60% in oil) at room temperature in portions. After stirring for 2 h at room temperature, the reaction was quenched by the addition of 1M HCl to pH 7.0. The solution was diluted with ethyl acetate (50 mL) and was washed with water (50 mL) and brine (50 mL). The organic phase was dried over anhydrous Na_2SO_4, filtered and the filtrate was concentrated in vacuo. The resulting pale yellow oil was purified by flash column chromatography over silica gel (EtOAc/hexanes, 1/5, v/v) to give **21** (735 mg, 90%) as a pale yellow oil. R_f = 0.5 (EtOAc/hexanes, 1/4, v/v); $[\alpha]_D^{20}$ = +46.0° (c = 3.0, $CHCl_3$); IR (thin film, cm^{-1}): ν_{max} = 696, 735, 847, 912, 1027, 1071, 1087, 1208, 1273, 1360, 1453, 1496, 1604, 1725, 2866,2908, 3030, 3063; ^1H NMR (400 MHz, $CDCl_3$) δ 7.49 (dd, *J* = 7.8, 1.6 Hz, 2H), 7.45 – 7.29 (m, 26H), 7.27 – 7.06 (m, 7H), 5.94 (d, *J* = 3.7 Hz, 1H, C-1^{II}), 5.09 – 5.00 (m, 3H), 4.92 – 4.86 (m, 3H), 4.85 – 4.78 (m, 2H), 4.76 – 4.57 (m, 6H, C-1^{I}), 4.44 (d, *J* = 11.1 Hz, 1H), 4.34 (d, *J* = 12.1 Hz, 1H), 4.22 – 4.17 (dd, *J* = 8.8, 5.2 Hz, 1H), 4.12 – 4.02 (m, 1H), 4.00 – 3.91 (m, 1H), 3.86 – 3.76 (m, 3H), 3.74 – 3.68 (m, 2H), 3.63 – 3.56 (m, 1H), 3.38 (dd, *J* = 10.9, 1.7 Hz, 1H), 3.24 (dd, *J* = 10.9, 2.6 Hz, 1H), 2.95 – 2.78 (m, 2H), 1.38 (t, *J* = 7.5 Hz, 3H); ^{13}C NMR (101 MHz, $CDCl_3$) δ 138.7, 138.6, 138.0, 137.9, 137.8 (2C), 137.7, 128.2 (2C), 128.1 (4C), 127.9 (2C), 127.8, 127.7 (2C), 127.6, 127.4 (2C), 127.3, 95.5 (C-1^{II}), 84.6 (C-1^{I}), 84.5, 81.7, 79.4, 78.8, 78.7, 77.4, 75.5, 75.4, 74.8, 74.6, 74.4, 73.3, 73.2, 72.9, 69.9, 68.8, 67.8, 24.3, 14.6; HRMS: m/z: calcd. for $C_{63}H_{68}O_{10}S$ $[M+Na]^+$: 1039.4431; found: 1039.4436.

N-Benzyl-N-benzyloxycarbonyl-5-aminopentyl O-[2,3,4,6-tetra-O-benzyl-α-D-glucopyranosyl]-(1→2)-O-(3,4,6-tri-O-benzyl)-(1→3)-2-azido-2-deoxy-β-D-mannopyranoside (22)

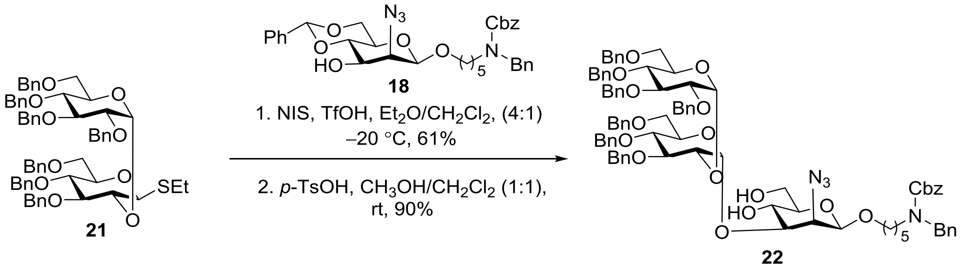

A mixture of disaccharide donor **21** (658 mg, 0.647 mmol, 1.3 equiv) and mannopyranosyl acceptor **18** (300 mg, 1.527 mmol, 1.0 equiv) in CH_2Cl_2/Et_2O (1:4, 20 mL) was stirred under an atmosphere of Ar for 30 min. The reaction mixture was cooled (−20 °C) and NIS (146 mg, 0.647 mmol, 1.3 equiv) was added followed by the addition of TfOH (22 μL, 0.249 mmol, 0.5 equiv). After stirring for 45 min, the reaction mixture was quenched by the addition of Et_3N (1.0 mL). The organic solution was diluted with CH_2Cl_2 (25 mL) and the mixture was washed with 10% $Na_2S_2O_3$ (50 mL) and brine (100 mL). The organic phase was dried over anhydrous Na_2SO_4, filtered and the filtrate was concentrated in vacuo. HRMS: m/z: calcd. for $C_{94}H_{100}N_4O_{17}$ $[M+Na]^+$: 1579.6981; found: 1579.6896. To a solution of crude trisaccharide (100 mg, 0.064 mmol, 1.0 equiv) in 10 mL of CH_2Cl_2/MeOH (1:1) at room temperature was added *p*-TsOH (1.2 mg, 6.42 μmol, 0.1 equiv). After stirring for 6 h, the reaction mixture was quenched by the addition of aq. $NaHCO_3$ (10 mL), extracted in EtOAc (50 mL), dried over anhydrous Na_2SO_4 and concentrated in vacuo. The resulting yellow oil was purified by flash column chromatography over silica gel (EtOAc/hexanes, 1/1, v/v) to give diol **22** (85 mg, 90%) as a colorless oil. R_f = 0.3 (EtOAc/hexanes, 1/1, v/v); $[\alpha]_D^{20}$ = +7.1° (c = 3.0, $CHCl_3$); IR (thin film, cm^{-1}): ν_{max} = 697, 749, 764, 1024, 1070, 1133, 1260, 1275, 1361, 1419, 1454, 1494, 1698, 2107, 2869, 2908; ^1H NMR (400 MHz, $CDCl_3$) δ 7.43 – 7.20 (m, 37H), 7.20 – 7.04 (m, 8H), 5.19 (d, *J* = 9.8 Hz, 2H), 5.13 (d, *J* = 3.5 Hz, 1H, C-1^{III}), 4.99 – 4.90 (m, 4H), 4.89 – 4.84 (m, 2H, C-1^{II}), 4.82 (d, *J* = 4.5 Hz, 1H), 4.77 (d, *J* = 11.0 Hz, 1H), 4.70 (d, *J* =

12.0 Hz, 1H), 4.65 – 4.47 (m, 7H), 4.42 (t, *J* = 12.2 Hz, 2H, C-1^{I}), 4.24 (d, *J* = 12.1 Hz, 1H), 4.19 – 4.09 (m, 2H), 4.09 – 4.03 (m, 2H), 4.00 (br d, *J* = 10.1 Hz, 1H), 3.79 – 3.66 (m, 6H), 3.63 (d, *J* = 9.4 Hz, 2H), 3.58 (dd, *J* = 9.9, 3.5 Hz, 2H), 3.40 (dd, *J* = 9.3, 3.6 Hz, 1H), 3.31 (dd, *J* = 10.9, 2.2 Hz, 2H), 3.20 (d, *J* = 10.0 Hz, 2H), 3.15 – 3.06 (m, 1H), 1.64 – 1.49 (br m, 4H), 1.40 – 1.29 (br m, 2H); ^{13}C NMR (101 MHz, $CDCl_3$) δ 156.6, 156.1 (Cbz, NHCOO), 138.3 (2C), 138.1, 137.8 (2C), 137.7, 137.6 (2C), 128.5, 128.4, 128.3 (3C), 128.2 (2C), 128.1, 128.0, 127.8, 127.7, 127.6, 127.5, 100.0 (C-1^{I}), 99.5 (C-1^{III}), 97.4 (C-1^{II}), 82.3, 79.8, 78.3, 78.0, 77.3, 76.0, 75.9, 75.7, 75.4, 74.9, 74.7, 74.0, 73.5, 73.2, 71.4, 70.4, 67.1, 63.4, 50.4, 50.2, 47.0, 46.0, 29.1, 29.0, 27.8, 27.1, 23.0, 22.8; HRMS: m/z: calcd. for $C_{87}H_{96}N_4O_{17}$ $[M+Na]^+$: 1491.6668; found: 1491.6608.

***N*-Benzyl-*N*-benzyloxycarbonyl-5-aminopentyl *O*-[2,3,4,6-tetra-*O*-benzyl-α-D-glucopyranosyl]-(1→2)-*O*-(3,4,6-tri-*O*-benzyl)-(1→3)-2-azido-6-*O*-*tert*-butyldimethylsilyl-2-deoxy-β-D-mannopyranoside (3)**

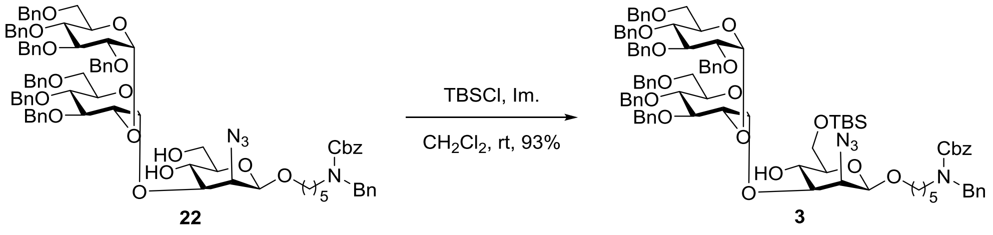

To a solution of diol **22** (200 mg, 0.136 mmol, 1.0 equiv) in DMF (5 mL) was added imidazole (19 mg, 0.272 mmol, 2.0 equiv) and TBSCl (31 mg, 0.204 mmol, 1.5 equiv) and the contents were stirred for 8 h at room temperature. The reaction mixture was quenched by addition of aq. NH_4Cl (10 mL), extracted with EtOAc (20 mL), washed with brine (25 mL), dried over anhydrous Na_2SO_4 and concentrated in vacuo. The residue was purified by column chromatography (EtOAc/hexanes, 1/4, v/v) to give **3** (195 mg, 93%) as a pale yellow oil. R_f = 0.5 (EtOAc/hexanes, 4/6, v/v); $[\alpha]_D^{20}$ = +43.6° (c = 3.0, $CHCl_3$); IR (thin film, cm^{-1}): ν_{max} = 697, 736, 836, 1028, 1069, 1132, 1212, 1252, 1362, 1421, 1454, 1496, 1605, 1730, 2106,

2859, 2927, 3031, 3475; ^{1}H NMR (400 MHz, $CDCl_3$) δ 7.47 – 7.27 (m, 36H), 7.23 – 7.09 (m, 9H), 5.22 (t like, *J* = 7.3 Hz, 3H, C-1^{III}), 4.99 (d, *J* = 3.5 Hz, 1H), 4.98 – 4.92 (m, 5H, C-1^{II}), 4.91 – 4.75 (m, 4H), 4.65 (d, *J* = 12.0 Hz, 1H), 4.59 – 4.51 (m, 5H), 4.50 – 4.43 (m, 2H, C-1^{I}), 4.30 (d, *J* = 12.1 Hz, 1H), 4.21 – 4.16 (m, 1H), 4.15 – 4.08 (m, 2H), 4.07 – 3.99 (m, 2H), 3.95 – 3.87 (m, 2H), 3.84 (dd, *J* = 11.2, 2.5 Hz, 2H), 3.81 – 3.73 (m, 3H), 3.72 – 3.68 (m, 2H), 3.68 – 3.64 (m, 1H), 3.62 (d, *J* = 3.4 Hz, 1H), 3.46 (dd, *J* = 9.2, 3.6 Hz, 1H), 3.39 (dd, *J* = 11.0, 2.6 Hz, 1H), 3.28 (dd, *J* = 10.9, 1.6 Hz, 2H), 3.13 (ddd, *J* = 9.3, 6.5, 2.5 Hz, 1H), 1.61 (br s, 4H), 1.37 (br s, 2H), 0.94 (s, 9H), 0.10 (s, 6H); ^{13}C NMR (101 MHz, $CDCl_3$) δ 138.4 (2C), 138.1, 137.9, 137.8, 137.7 (3C), 128.4, 128.2 (3C), 128.1 (2C), 128.0, 127.9, 127.8 (2C), 127.7, 127.6, 127.5, 99.7 (C-1^{I}), 99.2 (C-1^{III}), 97.0 (C-1^{II}), 82.8, 82.2, 79.9, 78.2, 77.9, 75.3, 74.8, 74.6, 73.4, 73.2, 71.3, 70.4, 67.0, 63.2, 50.4, 50.1, 47.0, 46.1, 29.1, 27.7, 27.3, 25.7, 23.0, 18.2, -5.2, -5.3; HRMS: m/z: calcd. for $C_{93}H_{110}N_4O_{17}Si$ $[M+Na]^+$: 1605.7533; found: 1605.7356.

***tert*-Butyldiphenylsilyl *O*-2-azido-4,6-*O*-benzylidene-2-deoxy-β-D-galactopyranoside (26)**

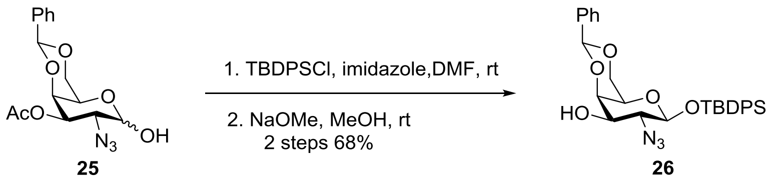

To a solution of hemiacetal **25**[6] (1.00 g, 2.108 mmol, 1.0 equiv) in DMF (10 mL) was added imidazole (0.359 g, 5.27 mmol, 2.5 equiv) and TBDPSCl (0.869 g, 3.16 mmol, 1.5 equiv) and stirred for 8 h at rt. The reaction mixture was quenched with aq. NH_4Cl (10 mL), extracted with EtOAc (50 mL), washed with brine (25 mL), dried over anhydrous Na_2SO_4 and concentrated in vacuo. The residue (0.9 g) was dissolved in methanol (10 mL) and NaOMe (0.627 mL, 0.314 mmol, 0.2 equiv, 0.5 M in MeOH) was added. The solution was stirred for 2 h, neutralized with Amberlite 120 (H^+) resin, filtered and concentrated. The residue was purified by column chromatography (EtOAc/hexanes, 1/9, v/v) to give **26** (0.80 g, 68%) as a

pale yellow solid. R_f = 0.6 (EtOAc/hexanes, 1/4, v/v); $[\alpha]_D^{20}$ = –0.77° (c = 3.0, $CHCl_3$); IR (thin film, cm^{-1}): ν_{max} = 666, 699, 739, 758, 799, 822, 874, 886, 905, 921, 939, 968, 995, 1056, 1089, 1167, 1215, 1259, 1286, 1315, 1364, 1393, 1427, 1458, 1472, 1589, 2110, 2858, 2931, 3072, 3355; ^1H NMR (400 MHz, $CDCl_3$) δ 7.86 – 7.75 (m, 4H), 7.55 – 7.51 (m, 2H), 7.46 – 7.36 (m, 9H), 5.48 (s, 1H, benzylidene CH), 4.44 (d, *J* = 7.7 Hz, 1H, C-1^I), 4.00 (dd, *J* = 3.8, 0.8 Hz, 1H), 3.94 (dd, *J* = 12.3, 1.4 Hz, 1H), 3.83 (dd, *J* = 12.4, 1.8 Hz, 1H), 3.68 (dd, *J* = 10.2, 7.7 Hz, 1H), 3.40 (dd, *J* = 10.2, 3.7 Hz, 1H), 2.94 (d, *J* = 1.1 Hz, 1H), 1.18 (s, 9H); ^{13}C NMR (101 MHz, $CDCl_3$) δ 137.5, 135.9, 135.7, 133.2, 132.8, 129.7, 129.5, 129.2, 128.2, 127.4, 127.2, 126.3, 101.1 (benzylidene CH), 96.6 (C-1^I), 74.3, 71.2, 68.5, 66.6, 66.1, 26.8, 19.1; HRMS: m/z: calcd. for $C_{29}H_{33}N_3O_5Si$ $[M+Na]^+$: 554.2087; found: 554.2058.

2-Azido-3,4-di-*O*-acetyl-2-deoxy-α-L-fucopyranosyl trichloroacetimidate (8)

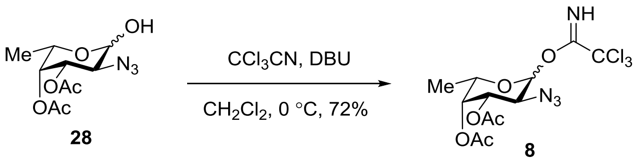

To a solution of **28**[7] (1.00 g, 3.66 mmol, 1.0 equiv) in dichloromethane (16 mL) was added trichloroacetonitrile (3.67 mL, 36.6 mmol, 10.0 equiv) and cooled to 0 °C. DBU (55 μL, 0.366 mmol, 0.1 equiv) was added and the contents were stirred for 4 h. The reaction mixture was concentrated in vacuo and the resulting crude yellow oil was purified by flash column chromatography over silica gel (EtOAc/hexanes, 1/9, v/v) to give **8** (1.10 g, 72%) as a colorless oil. ^1H NMR (400 MHz, $CDCl_3$) δ 8.76 (s, 1H), 5.67 (d, *J* = 8.5 Hz, 1H, C-1), 5.23 (dd, *J* = 3.4, 1.1 Hz, 1H), 4.90 (dd, *J* = 10.8, 3.4 Hz, 1H), 3.98 – 3.87 (m, 2H), 2.20 (s, 3H), 2.06 (s, 3H), 1.23 (d, *J* = 6.4 Hz, 3H); ^{13}C NMR (101 MHz, $CDCl_3$) δ 170.3, 169.6, 160.9, 96.7, 71.6, 70.3, 69.2, 60.3, 20.6, 20.5, 15.9.

tert-Butyldiphenylsilyl *O*-(3,4-di-*O*-acetyl-2-azido-2,6-dideoxy-α–L-fucopyranosyl)-(1→3)-2-azido-4,6-*O*-benzylidene-2-deoxy-β-D-galactopyranoside (31)

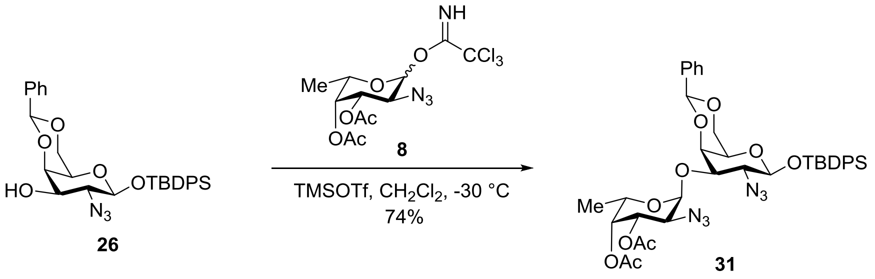

A mixture of fucosyl donor **8** (236 mg, 0.564 mmol, 1.5 equiv) and acceptor **26** (200 mg, 0.376 mmol, 1.0 equiv) in CH_2Cl_2 (10 mL) was stirred under an atmosphere of Ar for 30 min. The reaction mixture was cooled (−30 °C) and TMSOTf (20 μL, 0.113 mmol, 0.3 equiv) was added. After stirring for 45 min, the reaction mixture was quenched by the addition of Et_3N (1.0 mL). The mixture was concentrated in vacuo. The resulting dark yellow oil was purified by flash column chromatography over silica gel (EtOAc/hexanes, 1/5, v/v) to give **31** (α–isomer only, 220 mg, 74%) as a colorless oil. R_f = 0.6 (EtOAc/hexanes, 1/4, v/v); $[\alpha]_D^{20}$ = –26.3° (c = 2.0, $CHCl_3$); IR (thin film, cm^{-1}): ν_{max} = 701, 750, 764, 802, 822, 899, 973, 999, 1056, 1092, 1173, 1260, 1275, 1367, 1428, 1458, 1751, 2114, 2860, 2989, 3006; ^1H NMR (600 MHz, $CDCl_3$) δ 7.80 (dd, *J* = 7.3, 0.4 Hz, 2H), 7.73 (d, *J* = 6.9 Hz, 2H), 7.51 (d, *J* = 6.9 Hz, 2H), 7.47 – 7.31 (m, 9H), 5.44 (s, 1H, benzylidene CH), 5.37 (dd, *J* = 11.2, 3.2 Hz, 1H), 5.22 (d, *J* = 3.1 Hz, 1H, C-1^{II}), 5.08 (d, *J* = 3.6 Hz, 1H), 4.46 (d, *J* = 7.7 Hz, 1H, C-1^{I}), 4.35 (d, *J* = 6.6 Hz, 1H), 4.12 (d, *J* = 3.5 Hz, 1H), 3.96 – 3.88 (m, 2H), 3.86 – 3.80 (m, 1H), 3.64 (dd, *J* = 11.2, 3.6 Hz, 1H), 3.32 (dd, *J* = 10.5, 3.5 Hz, 1H), 2.96 (s, 1H), 2.14 (s, 3H), 2.04 (s, 3H), 1.15 (s, 9H), 1.00 (d, *J* = 6.6 Hz, 3H, fucose methyl); ^{13}C NMR (151 MHz, $CDCl_3$) δ 170.2, 169.6, 137.4, 136.0, 135.8, 133.2, 132.9, 129.7, 129.6, 129.3, 128.4, 127.4, 127.2, 126.1, 101.2 (benzylidene CH), 100.6 (C-1^{II}), 97.1 (C-1^{I}), 80.3, 74.5, 70.4, 68.7, 68.3, 65.9, 65.4, 64.2, 57.4, 26.8, 20.6, 20.5, 19.1, 16.0 (fucose methyl); HRMS: m/z: calcd. for $C_{39}H_{46}N_6O_{10}Si$ $[M+Na]^+$: 809.2942; found: 809.2893.

***tert*-Butyldiphenylsilyl *O*-(3,4-di-*O*-acetyl-2-azido-2,6-dideoxy-α–L-fucopyranosyl)-(1→3)-2-azido-4,6-*O*-benzylidene-2-deoxy-β-D-galactopyranoside (34)**

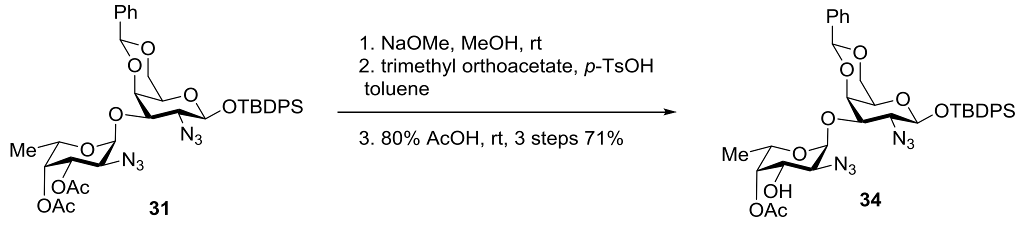

Disaccharide **31** (200 mg, 0.254 mmol, 1.0 equiv) was dissolved in methanol (10 mL) and NaOMe (0.25 mL, 0.127 mmol, 0.5 equiv, 0.5 M in MeOH) was added. The contents were stirred for 2 h, quenched with Amberlite 120 (H^+) resin, filtered and concentrated to obtain the diol **32**. R_f = 0.4; IR (thin film, cm^{-1}): ν_{max} = 701, 750, 764, 796, 820, 992, 1056, 1093, 1165, 1260, 1275, 1363, 1425, 1456, 2116, 2990; ^{1}H NMR (400 MHz, $CDCl_3$) δ 7.74 – 7.63 (m, 4H), 7.44 – 7.39 (m, 2H), 7.47 – 7.13 (m, 9H), 5.35 (s, 1H, benzylidene CH), 4.90 (d, *J* = 3.7 Hz, 1H, C-1^{II}), 4.38 (d, *J* = 7.7 Hz, 1H, C-1^{I}), 4.03 (q, *J* = 7.1 Hz, 2H), 3.90 – 3.68 (m, 4H), 3.53 (d, *J* = 2.5 Hz, 1H), 3.33 (dd, *J* = 10.7, 3.7 Hz, 1H), 3.20 (dd, *J* = 10.5, 3.5 Hz, 1H), 2.86 (s, 1H), 1.19 (d, *J* = 6.7 Hz, 3H), 1.06 (s, 9H). ^{13}C NMR (101 MHz, $CDCl_3$) δ 137.7, 135.9, 135.7, 134.5, 133.2, 132.7, 129.7, 129.5, 129.2, 129.0, 128.3, 128.2, 127.4, 127.2, 126.1, 125.9, 101.1 (benzylidene CH), 100.8 (C-1^{II}), 97.0 (C-1^{I}), 79.9, 74.6, 71.5, 68.6, 67.8, 66.5, 65.8, 64.5, 59.9, 26.8, 19.1, 16.2 (fucose methyl); HRMS: m/z: calcd. for $C_{35}H_{42}N_6O_8Si$ $[M+Na]^+$: 725.2731; found: 725.2703. The crude diol **32** was dissolved in anhydrous toluene and trimethyl orthoacetate (68 mg, 0.569 mmol, 2.0 equiv) was added followed by *p*-TsOH (11 mg, 0.057 mmol, 0.2 equiv) at room temperature. The contents were stirred for 30 min and then quenched with Et_3N (0.25 mL), and diluted with CH_2Cl_2 (25 mL). The organic layer was washed with brine (25 mL), dried over anhydrous Na_2SO_4, filtered and concentrated. The crude residue **33** was dissolved in 80% acetic acid and the solution was stirred for 1 h and concentrated under reduced pressure to give a yellow oil, which was purified by flash column chromatography over silica gel (EtOAc/hexanes, 1/5, v/v) to give **34** (150 mg, 71%) as a

colorless oil. R_f = 0.5 (EtOAc/hexanes, 1/4, v/v); $[\alpha]_D^{20}$ = –0.67° (c = 2.0, $CHCl_3$); IR (thin film, cm^{-1}): ν_{max} = 699, 750, 803, 822, 969, 996, 1048, 1093, 1260, 1275, 1369, 1427, 1454, 1740, 2116, 2857, 2928; ^1H NMR (400 MHz, $CDCl_3$) δ 7.99 – 7.68 (m, 4H), 7.64 – 7.24 (m, 11H), 5.42 (s, 1H, benzylidene CH), 5.11 (d, *J* = 3.4 Hz, 1H), 5.03 (d, *J* = 3.6 Hz, 1H, C-1^{II}), 4.45 (d, *J* = 7.7 Hz, 1H, C-1^{I}), 4.32 – 4.22 (m, 2H), 4.10 (d, *J* = 3.6 Hz, 1H), 3.97 – 3.80 (m, 3H), 3.46 (dd, *J* = 10.6, 3.5 Hz, 1H), 3.32 (dd, *J* = 10.3, 3.4 Hz, 1H), 2.96 (s, 1H), 2.16 (s, 3H), 1.14 (s, 9H), 1.04 (d, *J* = 6.6 Hz, 3H, fucose-Me); ^{13}C NMR (101 MHz, $CDCl_3$) δ 171.2, 137.6, 135.9, 135.7, 133.1, 132.7, 129.7, 129.5, 129.2, 128.3, 127.4, 127.1, 126.1, 101.1 (benzylidene CH), 100.8 (C-1^{I}), 97.0 (C-1^{II}), 80.1, 74.5, 72.9, 68.6, 66.5, 65.8, 65.5, 64.4, 60.3, 26.7, 20.6, 19.1, 16.2 (fucose methyl); HRMS: m/z: calcd. for $C_{37}H_{44}N_6O_9Si$ $[M+Na]^+$: 767.2837; found: 767.2804.

***tert*-Butyldiphenylsilyl *O*-[4-benzoyl-2,3,6-tri-*O*-benzyl-α–D-galactopyranosyl]-(1→3)-*O*-(4-*O*-acetyl-2-azido-2,6-dideoxy-α–L-fucopyranosyl)-(1→3)-2-azido-4,6-*O*-benzylidene-2-deoxy-β-D-galactopyranoside (35)**

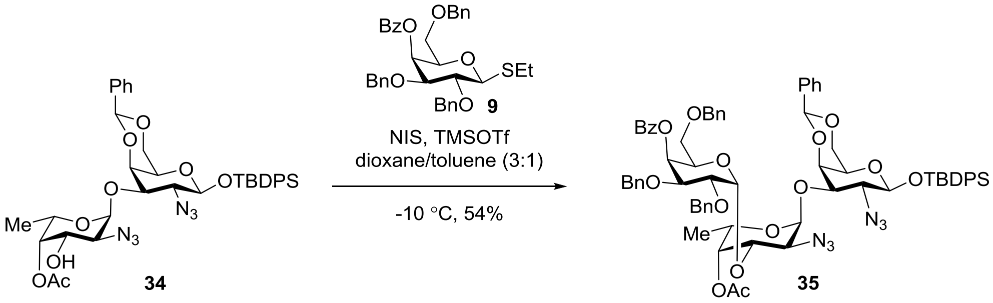

A mixture of galactosylating agent **9**[8] (157 mg, 0.262 mmol, 1.5 equiv) and disaccharide acceptor **34** (130 mg, 0.175 mmol, 1.0 equiv) in dioxane/toluene (3:1, 12 mL) was stirred under an atmosphere of Ar for 30 min. The reaction mixture was cooled (−10 °C) and NIS (59 mg, 0.262 mmol, 1.5 equiv) was added followed by the addition of TMSOTf (3.15 μL, 0.017 mmol, 0.1 equiv). After stirring for 2 h, the reaction mixture was quenched by the addition of Et_3N (0.25 mL). The organic solution was diluted with EtOAc (25 mL) and the mixture was

washed with 10% $Na_2S_2O_3$ (25 mL) and brine (50 mL). The organic phase was dried over anhydrous Na_2SO_4, filtered and the filtrate was concentrated in vacuo. The resulting dark yellow oil was purified by flash column chromatography over silica gel (EtOAc/hexanes, 1/5, v/v) to give **35** (α–isomer, 120 mg, 54%) as a pale yellow oil. R_f = 0.6 (EtOAc/hexanes, 1/4, v/v); $[\alpha]_D^{20}$ = +4.1° (c = 2.5, $CHCl_3$); IR (thin film, cm^{-1}): ν_{max} = 666, 698, 747, 803, 822, 874, 938, 971, 999, 1026, 1056, 1093, 1175, 1216, 1229, 1270, 1313, 1366, 1428, 1453, 1496, 1602, 1722, 2115, 2860, 2932, 3028; ^{1}H NMR (400 MHz, $CDCl_3$) δ 7.99 – 7.83 (m, 2H), 7.77 – 7.62 (m, 4H), 7.54 – 7.00 (m, 29H), 5.76 (d, *J* = 1.6 Hz, 1H), 5.29 (s, 1H, benzylidene CH), 5.14 (d, *J* = 2.3 Hz, 1H), 5.11 (d, *J* = 3.3 Hz, 1H, C-1^{III}), 5.06 (d, *J* = 3.7 Hz, 1H, C-1^{II}), 4.75 (d, *J* = 11.6 Hz, 1H), 4.69 (d, *J* = 4.1 Hz, 1H), 4.49 (d, *J* = 11.6 Hz, 1H), 4.39 (d, *J* = 7.7 Hz, 2H, C-1^{I}), 4.31 – 4.23 (m, 3H), 4.20 – 4.12 (m, 1H), 4.09 (dd, *J* = 10.8, 3.2 Hz, 1H), 4.03 (dd, *J* = 13.8, 5.3 Hz, 1H), 3.90 – 3.81 (m, 4H), 3.75 (dd, *J* = 12.2, 1.5 Hz, 1H), 3.68 (dd, *J* = 10.9, 3.6 Hz, 1H), 3.39 (d, *J* = 6.2 Hz, 2H), 3.28 (dd, *J* = 10.4, 3.5 Hz, 1H), 2.89 (s, 1H), 1.97 (s, 3H), 1.08 (s, 9H), 0.89 (d, *J* = 6.5 Hz, 3H, fucose methyl); ^{13}C NMR (101 MHz, $CDCl_3$) δ 170.6, 165.6, 138.3, 138.2, 137.9, 137.6, 136.0, 135.8, 133.2, 132.9, 132.9, 130.0, 129.8, 129.7, 129.6, 129.1, 128.3 (2C), 128.2, 128.1 (2C), 127.7, 127.5, 127.4, 127.3, 127.2 (2C), 126.2, 101.1 (CH), 100.6 (C-1^{II}), 99.3 (C-1^{III}), 97.0 (C-1^{I}), 80.2, 75.7, 74.6, 74.4, 73.2, 73.1, 72.7, 72.4, 71.4, 68.7 (2C), 68.6 (2C), 66.1, 65.9, 64.4, 60.2, 26.8, 20.7, 19.2, 16.2 (fucose methyl); HRMS: m/z: calcd. for $C_{71}H_{76}N_6O_{15}Si$ $[M+Na]^+$: 1303.5036; found: 1303.4989.

O-[4-Benzoyl-2,3,6-tri-O-benzyl-α–D-galactopyranosyl]-(1→3)-O-(4-O-acetyl-2-azido-2,6-dideoxy-α–L-fucopyranosyl)-(1→3)-2-azido-4,6-O-benzylidene-2-deoxy-β-D-galactopyranoside trichloroacetimidate (2)

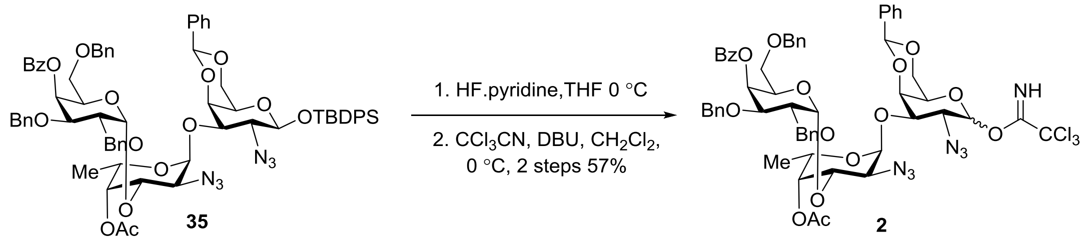

Trisaccharide **35** (250 mg, 0.195 mmol, 1.0 equiv) was dissolved in THF/pyridine (4:1, 5 mL) and the solution was cooled to 0 °C. HF-pyridine (0.17 mL, 5.857 mmol, 30.0 equiv, 70% in pyridine) was added and the contents were stirred for 24 h. The reaction mixture was quenched with aq. $NaHCO_3$, extracted with EtOAc (25 mL), washed with brine (50 mL), dried over anhydrous Na_2SO_4, filtered and concentrated in vacuo. The crude residue was dissolved in CH_2Cl_2 (10 mL) and cooled to 0 °C. Trichloroacetonitrile (0.10 mL, 1.150 mmol, 10 equiv) was added followed by DBU (3.5 μL, 23.00 μmol, 0.2 equiv). The contents were stirred for 6 h and concentrated in vacuo. The residue was purified by column chromatography (EtOAc/hexanes, 1/3, v/v) to give a non-separable mixture of β and α isomers of **2** (β/α =1:4, 130 mg, 57%) as a colorless oil: resonances attributable to major *α*-isomer: ^{1}H NMR (400 MHz, d_6-acetone) δ 9.43 (s, 1H), 7.99 (d, *J* = 7.6 Hz, 2H), 7.66 – 7.09 (m, 23H), 6.67 (s, 1H), 5.89 (s, 1H), 5.67 (s, 1H), 5.47 (s, 1H), 5.30 (br s, 2H), 5.00 – 4.68 (m, 4H), 4.60 (d, *J* = 11.9 Hz, 1H), 4.53 – 4.33 (m, 6H), 4.28 – 4.12 (m, 3H), 4.09 – 3.92 (m, 3H), 3.47 (d, *J* = 6.1 Hz, 1H), 2.89 (s, 2H), 2.08 (s, 3H), 1.02 (d, *J* = 6.4 Hz, 3H); ^{13}C NMR (101 MHz, d_6-acetone) δ 171.1 (imidate CNH), 170.8 (acetate CO), 166.1 (benzoate CO), 139.9, 139.8, 139.6, 139.3, 130.4, 129.4, 128.9 (4C), 128.6, 128.5, 128.2, 128.1, 128.0, 127.0, 101.3 (benzylidene CH), 99.9, 99.8, 97.5, 80.2, 80.0, 76.6, 76.1, 75.3, 73.6, 73.6, 73.4, 73.3, 73.2, 72.5, 72.2, 72.2, 69.6, 69.4, 69.2, 68.1, 67.0, 63.3, 61.4, 16.6 (fucose methyl).

***N*-Benzyl-*N*-benzyloxycarbonyl-5-aminopentyl 2-azido-4,6-*O*-benzylidene-2-deoxy-3-naphthyl-β-D-mannopyranoside (17b)**

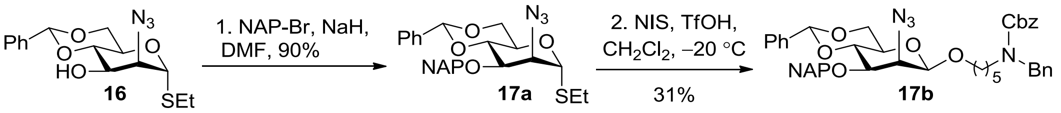

To a cooled (0 °C) solution of **16** (2.0 g, 5.92 mmol, 1.0 equiv) and 2-(bromomethyl)naphthalene (1.57 g, 7.11 mmol, 1.2 equiv) in DMF (20 mL) under an atmosphere of Ar was added sodium hydride (285 mg, 22.4 mmol, 60% in oil, 3.78 equiv) in portionwise. After stirring for 3 h at room temperature, the reaction was quenched by the addition of 1M HCl (10 mL). The organic phase was diluted with ethyl acetate (100 mL) and washed with water (2 × 150 mL) and brine (150 mL). The organic phase was dried over anhydrous Na_2SO_4, filtered and the filtrate was concentrated in vacuo. The resulting pale yellow oil was purified by flash column chromatography over silica gel (EtOAc/hexanes, 2/10, v/v) to give **17a** (2.54 g, 90%) as a pale yellow oil: R_f = 0.7 (EtOAc/hexanes, 2/10, v/v). R_f = 0.6 (EtOAc/hexanes, 1/4, v/v); $[\alpha]_D^{20}$ = +16.6° (c = 0.5, $CHCl_3$); ^1H NMR (400 MHz, $CDCl_3$) δ 8.00 – 7.75 (m, 4H), 7.69 – 7.39 (m, 8H), 5.70 (s, 1H, benzylidene CH), 5.31 (br s, 1H, C-1^I), 5.08 (d, *J* = 12.4 Hz, 1H), 4.94 (d, *J* = 12.4 Hz, 1H), 4.40 – 4.18 (m, 4H), 4.16 – 4.03 (m, 1H), 4.07 – 3.80 (m, 1H), 2.78 – 2.51 (m, 2H), 1.32 (td, *J* = 7.4, 0.9 Hz, 3H); ^{13}C NMR (101 MHz, $CDCl_3$) δ 137.2, 135.1, 133.1, 132.8, 128.8, 128.1, 128.0, 127.8, 127.5, 126.1, 126.0, 125.9, 125.7, 125.2, 101.5 (benzylidene CH), 83.3 (C-1^I), 79.1, 75.8, 73.0, 68.3, 64.2, 63.9, 25.2, 14.6; HRMS: m/z: calcd. for $C_{26}H_{27}N_3O_4S$ $[M+Na]^+$: 500.1620; found: 500.1603. A mixture of mannosyl donor **17a** (1.16 g, 2.443 mmol, 1.6 equiv) and *N*-benzyl-*N*-benzyloxycarbonyl-5-aminopentanol[2] (0.50 g, 1.5270 mmol, 1.0 equiv) in CH_2Cl_2 (22 mL) was stirred under an atmosphere of Ar for 30 min. The reaction mixture was cooled (−20 °C) and NIS (0.549 g, 2.443 mmol, 1.6 equiv) was added followed by the addition of TfOH (54 μL, 3.298 mmol, 2.16 equiv). After stirring for 45 min, the reaction mixture was quenched by the addition of Et_3N (0.5 mL). The organic solution was diluted with CH_2Cl_2 (30 mL) and the

mixture was washed with 10% $Na_2S_2O_3$ (50 mL) and brine (50 mL). The organic phase was dried over anhydrous Na_2SO_4, filtered and the filtrate was concentrated in vacuo. The resulting dark yellow oil was purified by flash column chromatography over silica gel (EtOAc/hexanes, 1/3, v/v) to give **17b** (β-isomer, 351 mg, 31%) and α-isomer (340 mg, 30%) as a pale yellow oil. β-isomer: R_f = 0.5 (EtOAc/hexanes, 4/6, v/v); $[\alpha]_D^{20}$ = –73.3° (c = 3.0, $CHCl_3$); IR (thin film, cm^{-1}): ν_{max} = 668, 697, 734, 779, 837, 884, 914, 967, 1004, 1030, 1085, 1122, 1169, 1215, 1251, 1305, 1361, 1380, 1420, 1454, 1471, 1496, 1606, 1697, 2106, 2857, 2929, 3033; ^{1}H NMR (400 MHz, $CDCl_3$) δ 7.92 – 7.80 (m, 3H), 7.62 – 7.16 (m, 19H), 5.64 (s, 1H, benzylidene CH), 5.20 (br d, *J* = 12.2 Hz, 2H), 5.04 (d, *J* = 12.6 Hz, 1H), 4.95 (d, *J* = 12.7 Hz, 1H), 4.55 – 4.46 (m, 3H, C1^I), 4.33 (dd, *J* = 10.3, 4.8 Hz, 1H), 4.08 (t, *J* = 9.5 Hz, 1H), 4.04 – 4.00 (m, 1H), 3.92 – 3.83 (m, 2H), 3.84 – 3.72 (m, 1H), 3.50 – 3.37 (m, 1H), 3.34 – 3.20 (m, 3H), 1.68 – 1.50 (m, 4H), 1.43 – 1.31 (m, 2H); ^{13}C NMR (101 MHz, $CDCl_3$) δ 156.6, 156.1, 137.8, 137.2, 135.1, 133.1, 133.0, 128.9, 128.4, 128.3, 128.2 (2C), 127.8 (2C), 127.7, 127.6, 127.2, 126.5, 126.1, 126.0, 125.9, 125.5, 101.5 (benzylidene CH), 100.2 ($J_{CH}\,\beta$ = 158 Hz, C-1^I), 78.5, 72.7, 68.3, 67.2, 67.0, 63.4, 50.4, 50.2, 47.0, 46.0, 29.0, 27.7, 27.3, 23.0; HRMS: m/z: calcd. for $C_{44}H_{46}N_4O_7$ $[M+Na]^+$: 765.3264; found: 765.3311.

N-Benzyl-*N*-benzyloxycarbonyl-5-aminopentyl 2-azido-6-*O*-benzoyl-2-deoxy-3-naphthyl-β-D-mannopyranoside (4)

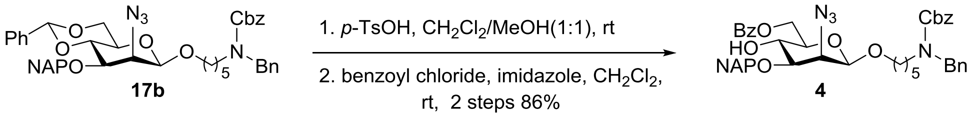

To a solution of **17b** (1.20 g, 1.615 mmol) in a mixture of CH_2Cl_2/MeOH (1:1) (24 mL) at room temperature was added *p*-TsOH (30 mg 2.5% by wt.). After stirring for 4 h, the reaction mixture was quenched by the addition of Et_3N (0.5 mL). The mixture was concentrated in vacuo. The resulting yellow oil was purified by flash column chromatography over silica gel (EtOAc/hexanes, 1/1, v/v) to give the corresponding diol as a pale yellow oil. To a solution of

the diol in CH_2Cl_2 (15 mL) at room temperature was added imidazole (329 mg, 4.843 mmol, 3.0 equiv) followed by addition of benzoyl chloride (0.37 mL, 3.228 mmol. 2.0 equiv). After stirring for 16 h, the reaction mixture was quenched by the addition of water and extracted with ethyl acetate (50 mL). The organic layer was washed with water (100 mL) and brine (100 mL), dried over anhydrous Na_2SO_4, filtered and the filtrate was concentrated in vacuo. The resulting pale yellow oil was purified by flash column chromatography over silica gel (EtOAc/hexanes, 3/7, v/v) to give **4** (1.05 g, 86%) as a pale yellow oil. R_f = 0.7 (EtOAc/hexanes, 3/7, v/v); $[\alpha]_D^{20}$ = –38.2° (c = 0.5, $CHCl_3$); IR (thin film): ν_{max} = 713, 750, 764, 820, 975, 1026, 1081, 1121, 1175, 1261, 1275, 1320, 1369, 1423, 1452, 1698, 1718, 2106, 2857, 2939; ^1H NMR (600 MHz, $CDCl_3$) δ 8.06 (d, J = 7.6 Hz, 2H), 7.93 – 7.79 (m, 4H), 7.60 – 7.14 (m, 16H), 5.18 (br d, J = 13.3 Hz, 2H), 4.94 (d, J = 12.0 Hz, 1H), 4.82 (d, J = 12.0 Hz, 1H), 4.70 – 4.57 (m, 2H), 4.51 – 4.45 (m, 3H, C-1^I), 4.02 – 3.94 (m, 1H), 3.94 – 3.80 (m, 2H), 3.59 – 3.48 (m, 2H), 3.46 – 3.34 (m, 1H), 3.32 – 3.13 (m, 2H), 1.63 – 1.46 (m, 4H), 1.36 – 1.25 (m, 2H); ^{13}C NMR (151 MHz, $CDCl_3$) δ 166.7, 156.7, 156.1, 137.8, 136.8, 136.6, 134.6, 133.2, 133.1, 133.1, 133.0 (2C), 129.7, 128.5, 128.4, 128.3 (2C), 128.2, 127.8 (2C), 127.7 (2C), 127.2, 127.1 (2C), 126.8, 126.2, 126.1, 125.5, 99.8 (C-1^I), 79.9, 74.2, 72.1, 69.6, 67.0, 66.7, 63.7, 61.0, 50.4, 50.1, 46.9, 46.0, 29.0, 27.7, 27.2, 23.0; HRMS: m/z: calcd. for $C_{44}H_{46}N_4O_8$ $[M+Na]^+$: 781.3213; found: 781.3229.

***N*-Benzyl-*N*-benzyloxycarbonyl-5-aminopentyl *O*-(3-*O*-acetyl-2-azido-4,6-*O*-benzylidene-2-deoxy-β-D-galactopyranosyl)-(1→4)-2-azido-6-*O*-benzoyl-2-deoxy-3-naphthyl-β-D-mannopyranoside (40)**

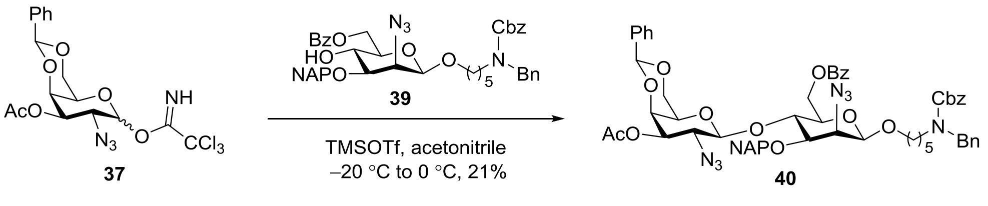

A mixture of azido-galactosyl donor **37**[6] (284 mg, 0.5929 mmol, 1.0 equiv) and azido-mannosyl acceptor **39** (500 mg, 0.6588 mmol, 1.11 equiv) in acetonitrile (9 mL) was stirred under an atmosphere of Ar for 30 min. The reaction mixture was cooled (−15 °C) and TMSOTf (17.8 μL, 65.888 μmol, 0.15 equiv) was added. After stirring for 45 min, the reaction mixture was quenched by the addition of Et_3N (0.5 mL). The mixture was concentrated in vacuo. The resulting dark yellow oil was purified by flash column chromatography over silica gel (EtOAc/hexanes, 2/3, v/v) to give **40** (β-isomer, 148 mg, 21%) and *α*-isomer (134 mg, 19%) as a pale yellow oil. β-isomer: R_f = 0.6 (EtOAc/hexanes, 4/6, v/v); $[\alpha]_D^{20}$ = +14.8° (c = 0.5, $CHCl_3$); IR (thin film): ν_{max} = 701, 717, 750, 766, 1026, 1070, 1101, 1177, 1226, 1260, 1275,1316, 1365, 1417, 1454, 1603, 1702, 1720, 1744, 2113, 2873, 2928; ^1H NMR (400 MHz, $CDCl_3$) δ 7.92 – 7.71 (m, 4H), 7.51 – 7.45 (m, 5H), 7.43 – 7.23 (m, 18H), 5.46 (s, 1H, benzylidene CH), 5.20 (d, *J* = 8.0 Hz, 3H), 5.11 (d, *J* = 12.4 Hz, 1H), 4.97 (d, *J* = 12.3 Hz, 1H), 4.76 (d, *J* = 12.0 Hz, 1H), 4.62 (dd, *J* = 10.5, 3.3 Hz, 1H), 4.56 – 4.49 (m, 3H, C-1^I), 4.42 (d, *J* = 8.1 Hz, 1H, C-1^{II}), 4.30 – 4.19 (m, 3H), 3.97 – 3.83 (m, 4H), 3.67 – 3.62 (m, 1H), 3.49 – 3.42 (m, 2H), 3.31 – 3.20 (m, 2H), 3.11 (s, 1H), 2.16 (s, 3H), 1.65 – 1.50 (m, 4H), 1.39 – 1.30 (m, 2H); ^{13}C NMR (101 MHz, $CDCl_3$) δ 170.3, 170.2, 138.1, 137.7, 137.6, 135.7, 133.0, 132.8, 128.9, 128.3, 128.2, 128.1, 128.0, 127.9, 127.7, 127.6 (2C), 127.5, 127.1, 126.1, 126.0, 125.9, 125.8, 125.7, 101.3 (C-1^{II}), 100.6 (benzylidene CH), 99.5, 78.3, 75.2, 75.1, 73.4 (2C), 73.3, 73.0, 72.4, 72.3, 69.4, 68.6, 68.4, 67.0, 65.9, 62.5, 61.0, 50.3, 50.0, 46.9, 46.0, 29.0, 27.7, 27.3, 23.0, 20.7; HRMS: m/z: calcd. for $C_{59}H_{61}N_7O_{13}$ $[M+Na]^+$: 1098.4225; found: 1098.4185.

***N*-Benzyl-*N*-benzyloxycarbonyl-5-aminopentyl *O*-(2-azido-3-*O*-benzoyll-4,6-*O*-benzylidene-2-deoxy-β-D-galactopyranosyl)-(1→4)-2-azido-6-*O*-benzoyl-2-deoxy-β-D-mannopyranoside (42)**

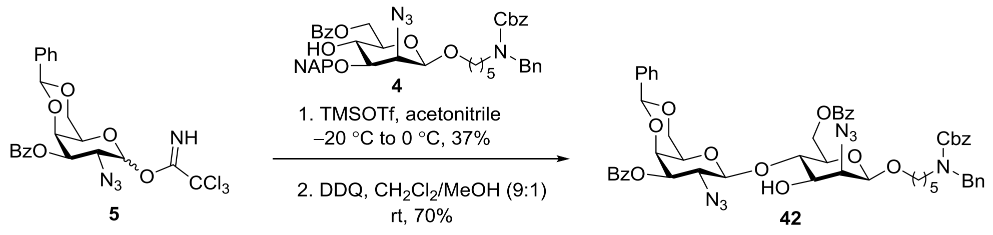

A mixture of azido-galactosyl donor **5** (571 mg, 1.0542 mmol, 2.0 equiv) and azido-mannosyl acceptor **4** (400 mg, 0.5271 mmol, 1.0 equiv) in acetonitrile (12 mL) was stirred under an atmosphere of Ar for 30 min. The reaction mixture was cooled (−20 °C) and TMSOTf (14.3 μL, 79.066 μmol, 0.15 equiv) was added. After stirring for 45 min, the reaction mixture was quenched by the addition of Et_3N (0.5 mL). The mixture was concentrated in vacuo. The resulting dark yellow oil was purified by flash column chromatography over silica gel (EtOAc/hexanes, 2/3, v/v) to give **41** (β-isomer, 220 mg, 37%) and the α-isomer (189 mg, 31%) each as a pale yellow oil. R_f = 0.55 (EtOAc/hexanes, 4/6, v/v); $[\alpha]_D^{20}$ = +18.1° (c = 0.5, $CHCl_3$); IR (thin film): ν_{max} = 695, 715, 750, 996, 1028, 1046, 1070, 1099, 1268, 1275, 1312, 1365, 1425, 1448, 1605, 1700, 1722, 2113, 2857, 2924; ^{1}H NMR (400 MHz, $CDCl_3$) δ 8.10 – 8.02 (m, 5H), 7.86 – 7.79 (m, 4H), 7.38 – 7.27 (m, 23H), 5.44 (s, 1H, benzylidene CH), 5.18 (d, *J* = 11.6 Hz, 2H), 5.01 (s, 1H), 4.91 (dd, *J* = 10.6, 3.0 Hz, 1H), 4.87 – 4.82 (m, 1H), 4.61 (dd, *J* = 12.9, 6.0 Hz, 2H, C-1^{II}), 4.58 – 4.42 (m, 4H, C-1^{I}), 4.37 (d, *J* = 3.1 Hz, 1H), 4.25 – 4.17 (m, 2H), 4.14 – 3.92 (m, 4H), 3.64 – 3.51 (m, 3H), 3.24 – 3.18 (m, 3H,), 1.57 – 1.53 (m, 4H), 1.29 – 1.26 (m, 2H); ^{13}C NMR (101 MHz, $CDCl_3$) δ 166.1, 165.7, 137.8, 137.5, 135.3, 133.5, 133.0 (2C), 132.9, 129.8, 129.6, 129.0, 128.9, 128.4 (3C), 128.3, 128.0, 127.8, 127.7 (2C), 127.2, 127.1, 101.9 (C-1^{II}), 100.5 (benzylidene CH), 99.6 (C-1^{I}), 75.6, 73.5, 73.3, 72.3, 69.5, 68.6, 67.1, 67.0, 66.3, 63.2, 62.6, 61.7, 61.5, 50.4, 50.1, 46.9, 46.0, 32.2, 29.0, 27.8, 27.3, 23.0, 22.8; HRMS: m/z: calcd. for $C_{64}H_{63}N_7O_{13}$ $[M+Na]^+$: 1160.4382; found:

1160.4410. To a solution of **41** (2.0 g, 1.7571 mmol, 1.0 equiv) in a mixture of CH_2Cl_2/MeOH (9:1, 60 mL) at room temperature was added DDQ (797 mg, 3.5142 mmol, 2.9 equiv). After stirring for 5 h, the reaction mixture was quenched by the addition of sat. $NaHCO_3$. The organic solution was diluted with CH_2Cl_2 (50 mL) and washed with aq. $NaHCO_3$ (100 mL) and brine (100 mL). The organic phase was dried over anhydrous Na_2SO_4, filtered and the filtrate was concentrated in vacuo. The resulting dark red oil was purified by flash column chromatography over silica gel (EtOAc/hexanes, 4/6, v/v) to give **42** (1.22 g, 70%) as a pale yellow oil. R_f = 0.4 (EtOAc/hexanes, 1/1, v/v); $[\alpha]_D^{20}$ = +13.7° (c = 1, $CHCl_3$); IR (thin film): ν_{max} = 701, 717, 750, 766, 994, 1028, 1068, 1167, 1264, 1275, 1312, 1367, 1419, 1452, 1494, 1452, 1583, 1601, 1692, 1722, 2114, 2857, 2928; ^1H NMR (400 MHz, $CDCl_3$) δ 8.27 – 7.95 (m, 4H), 7.62 – 7.50 (m, 2H), 7.48 – 7.37 (m, 6H), 7.37 – 7.12 (m, 13H), 5.49 (s, 1H, benzylidene CH^{II}), 5.16 (d, *J* = 9.4 Hz, 2H), 4.95 (dd, *J* = 10.7, 3.4 Hz, 1H), 4.76 (dd, *J* = 11.9, 1.6 Hz, 1H), 4.64 – 4.54 (m, 2H, $C\text{-}1^{I}$), 4.50 – 4.44 (m, 3H), 4.40 (d, *J* = 8.1 Hz, 2H, $C\text{-}1^{II}$), 4.14 – 3.98 (m, 3H), 3.91 – 3.78 (m, 3H), 3.68 – 3.59 (m, 2H), 3.51 – 3.34 (m, 1H), 3.29 – 3.10 (m, 2H), 1.67 – 1.39 (m, 4H), 1.37 – 1.26 (m, 2H); ^{13}C NMR (101 MHz, $CDCl_3$) δ 166.0, 165.5, 156.3, 155.8, 137.7, 137.1, 133.5, 133.0, 129.7 (2C), 129.5, 128.9, 128.8, 128.4, 128.3 (2C), 128.0, 127.6, 127.0, 125.9, 102.1 ($C\text{-}1^{II}$), 100.4 (benzylidene CH), 99.8 ($C\text{-}1^{I}$), 79.2, 72.8, 72.7, 72.1, 71.4, 69.5, 68.4, 66.9, 66.6, 62.9, 60.8, 50.3, 50.0, 46.8, 46.0, 28.9, 27.6, 27.1, 22.9; HRMS: m/z: calcd. for $C_{53}H_{55}N_7O_{13}$ $[M+Na]^+$: 1020.3756; found: 1020.3788.

_N_-Benzyl-_N_-benzyloxycarbonyl-5-aminopentyl _O_-(2-azido-3-_O_-benzoyl-4,6-_O_-benzylidene-2-deoxy-β-D-galactopyranosyl)-(1→4)-[_O_-3-benzyl-4,6-_O_-benzylidene]-(1→3)-2-azido-6-_O_-benzoyl-2-deoxy-β-D-mannopyranoside (45)

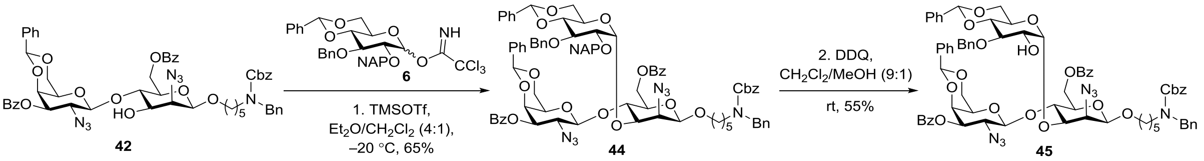

A mixture of glucosyl donor **6** (1.26 g, 1.955 mmol. 1.5 equiv) and disaccharide acceptor **42** (1.3 g, 1.303 mmol, 1.0 equiv) in a mixture of CH_2Cl_2/Et_2O (1:4, 52 mL) was stirred under an atmosphere of Ar for 30 min. The reaction mixture was cooled (–20 °C) and TMSOTf (94 μL, 0.521 mmol, 0.4 equiv) was added. After stirring for 45 min, the reaction mixture was quenched by the addition of Et_3N (0.5 mL). The mixture was concentrated in vacuo. The resulting dark yellow oil was purified by flash column chromatography over silica gel (EtOAc/hexanes, 2/3, v/v) to give trisaccharide **44** (alpha, 1.25 g, 65%) as a pale yellow oil: R_f = 0.5 (EtOAc/hexanes, 4/6, v/v); HRMS: m/z: calcd. for $C_{84}H_{83}N_7O_{18}$ $[M+Na]^+$: 1500.5692; found: 1500.5742. To a solution of **44** (1.25g, 0.8454 mmol, 2.0 equiv) in a mixture of CH_2Cl_2/MeOH (9:1, 25 mL) at room temperature was added DDQ (383 mg, 1.691 mmol, 2.0 equiv). After stirring for 5 h, the reaction mixture was quenched by the addition of sat. $NaHCO_3$. The organic phase was diluted with CH_2Cl_2 (50 mL) and washed with aq. $NaHCO_3$ (100 mL) and brine (100 mL). The organic phase was dried over anhydrous Na_2SO_4, filtered and the filtrate was concentrated in vacuo. The resulting dark red oil was purified by flash column chromatography over silica gel (EtOAc/hexanes, 4/6, v/v) to give **45** (622 mg, 55%) as a pale yellow oil. R_f = 0.5 (EtOAc/hexanes, 1/1, v/v); $[\alpha]_D^{20}$ = +7.6° (c = 0.5, $CHCl_3$); IR (thin film): ν_{max} = 764, 998, 1046, 1096, 1261, 1275, 1310, 1369, 1453, 1698, 1723, 2114, 2984, 3006; ^{1}H NMR (400 MHz, $CDCl_3$) δ 8.25 – 7.92 (m, 4H), 7.71 – 7.15 (m, 31H), 5.58 (s, 1H, benzylidene CH^{III}), 5.44 (s, 1H, benzylidene CH^{II}), 5.16 (d, _J_ = 4.2 Hz, 3H, C-1^{III}), 4.91 – 4.81 (m, 2H), 4.78 (s, 2H), 4.67 (dt, _J_ = 11.9, 6.0 Hz, 2H, C-1^{II}), 4.51 – 4.42 (m, 4H, C-1^{I}),

4.40 – 4.32 (m, 2H), 4.28 – 4.16 (m, 3H), 4.10 (dd, J = 10.7, 8.2 Hz, 1H), 3.98 (d, J = 11.4 Hz, 1H), 3.91 – 3.81 (m, 2H), 3.79 – 3.71 (m, 3H), 3.64 (t, J = 9.3 Hz, 1H), 3.54 – 3.45 (m, 1H), 3.47 – 3.31 (m, 2H), 3.27 – 3.15 (m, 2H), 1.68 – 1.45 (m, 4H), 1.36 –1.27 (m, 2H); ^{13}C NMR (101 MHz, $CDCl_3$) δ 165.9, 165.7, 138.7, 137.8, 137.3, 137.1, 133.4, 133.2, 129.8, 129.6, 129.0, 128.9, 128.8, 128.5, 128.4 (2C), 128.3, 128.1, 128.0 (2C), 127.9, 127.7, 127.1, 126.9, 126.0, 102.8 ($J_{CH}\,\alpha$ = 169 Hz, C-1^{III}), 102.2 ($J_{CH}\,\beta$ = 156 Hz, C-1^{I}), 101.9 (benzylidene CH^{III}), 101.2 (benzylidene CH^{II}), 99.9 ($J_{CH}\,\beta$ = 156 Hz, C-1^{II}), 81.5, 79.5, 74.3, 73.7, 73.6, 73.0, 72.9, 72.5, 68.9, 68.4, 67.2, 67.0, 63.7, 63.6, 60.9, 50.4, 50.1, 46.9, 46.0, 29.0, 27.7, 27.2, 22.9; HRMS: m/z: calcd. for $C_{73}H_{75}N_7O_{18}$ $[M+Na]^+$: 1360.5066; found: 1360.5006.

_N_-Benzyl-_N_-benzyloxycarbonyl-5-aminopentyl _O_-(2-azido-4,6-_O_-benzylidene-2-deoxy-β-D-galactopyranosyl)-(1→4)-[_O_-2,3,4,6-tri-_O_-benzyl-α-D-glucopyranosyl]-(1→2)-_O_-(3-benzyl-4,6-_O_-benzylidene-_α_-D-glucopyranosyl)-(1→3)-2-azido-2-deoxy-β-D-mannopyranoside (47)

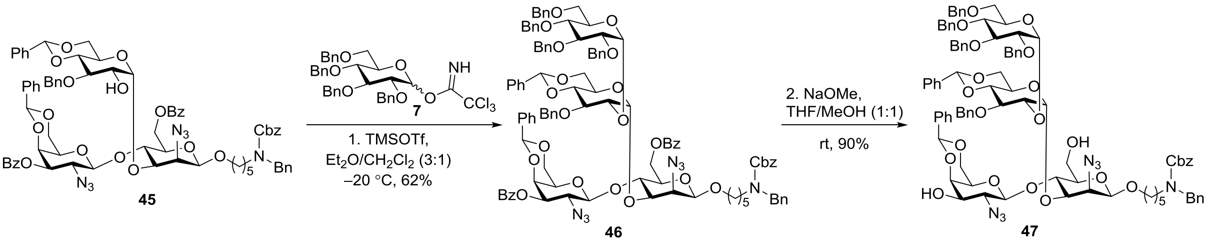

A mixture of glucosyl donor **7**[5] (524 mg, 0.896 mmol, 2.0 equiv) and trisaccharide acceptor **45** (600 mg, 0.448 mmol, 1.0 equiv) in a mixture of Et_2O/ CH_2Cl_2 (3:1, 18 mL) was stirred under an atmosphere of Ar for 30 min. The reaction mixture was cooled (−20 °C) and added TfOH (12 µL, 0.134 mmol, 0.3 equiv). After stirring for 45 min, the reaction mixture was quenched by the addition of Et_3N (1.0 mL). The mixture was filtered, and the filtrate was washed with 10% $Na_2S_2O_3$ (50 mL) and brine (50 mL). The organic phase was dried over anhydrous Na_2SO_4, filtered and the filtrate was concentrated in vacuo. The resulting dark yellow oil was purified by flash column chromatography over silica gel (EtOAc/hexanes, 1/3, v/v) to give **46** (alpha, 517 mg, 62%) as a pale yellow oil: R_f = 0.5 (EtOAc/hexanes, 3/7, v/v);

IR (thin film): ν_{max} = 669, 699, 743, 798, 822, 998, 1028, 1048, 1070, 1096, 1270, 1363, 1417, 1454, 1601, 1698, 1723, 2113, 2869, 2940; ^{1}H NMR (400 MHz, $CDCl_3$) δ 8.38 – 7.87 (m, 6H), 7.69 – 6.88 (m, 49H), 5.60 (s, 1H, benzylidene CH^{III}), 5.37 (s, 1H, benzylidene CH^{II}), 5.14 (br s, 3H, C-1^{III}), 5.03 (br s, 1H, C-1^{IV}), 4.97 – 4.71 (m, 10H), 4.67 (d, *J* = 8.3 Hz, 1H, C-1^{II}), 4.51 – 4.42 (m, 3H), 4.42 – 4.29 (m, 8H, C-1^{I}), 4.22 (d, *J* = 9.6 Hz, 1H), 4.18 – 4.11 (m, 4H), 3.89 – 3.72 (m, 4H), 3.69 (dd, *J* = 5.9, 3.7 Hz, 2H), 3.64 – 3.56 (m, 3H), 3.48 (t, *J* = 6.8 Hz, 1H), 3.23 – 2.97 (m, 5H), 1.76 – 1.46 (m, 4H), 1.45 – 1.28 (m, 2H); ^{13}C NMR (101 MHz, $CDCl_3$) δ 166.0, 165.6, 138.4, 138.2, 137.9, 137.8, 137.5, 137.4, 137.2, 133.5, 133.4, 132.9, 130.0, 129.8 (2C), 129.5, 129.0, 128.7, 128.4, 128.3, 128.2, 128.1 (2C), 128.0 (2C), 127.9, 127.8, 127.7, 127.5, 127.4, 127.2, 126.0, 125.9, 125.8, 125.7, 101.4 (C-1^{I}), 101.0 (C-1^{II}), 100.7 (benzylidene CH^{III}), 100.5 (benzylidene CH^{II}), 98.5 (C-1^{III}), 98.3 (C-1^{IV}), 82.6, 82.1, 79.5, 78.0, 77.0, 75.3, 75.1, 74.6, 73.4 (2C), 73.3, 73.0, 72.4 (2C), 72.3, 71.5, 70.5, 70.3, 70.0, 69.8, 69.1, 68.8, 68.6, 67.5, 66.9, 61.7, 60.6, 50.1, 49.6, 46.7, 45.8, 29.1, 27.4, 27.0, 22.9; HRMS: m/z: calcd. for $C_{107}H_{109}N_7O_{23}$ $[M+Na]^+$: 1882.7473; found: 1882.7443. To a solution of **46** (1.0 g, 0.537 mmol, 1.0 equiv) in a mixture of THF/MeOH (1:1) (10 mL) at room temperature was added 0.5M NaOMe in MeOH (0.214 mL, 0.2 equiv). After stirring for 12 h, the reaction mixture was neutralized by the addition of Amberlite 120 (H^+) resin, filtered and the filtrate was concentrated in vacuo. The resulting yellow oil was purified by flash column chromatography over silica gel (EtOAc/hexanes, 1/1, v/v) to give **47** (800 mg, 90%) as a colorless solid. R_f = 0.5 (EtOAc/hexanes, 1/1, v/v); $[\alpha]_D^{20}$ = +27.8° (c = 0.75, $CHCl_3$); IR (thin film): ν_{max} = 698, 749, 764, 822, 998, 1028, 1049, 1086, 1158, 1211, 1260, 1275, 1365, 1454, 1496, 1696, 2111, 2865, 2927, 3457; ^{1}H NMR (400 MHz, $CDCl_3$) δ 7.86 – 7.13 (m, 45H), 5.61 (s, 1H, benzylidene CH^{III}), 5.43 (s, 1H, benzylidene CH^{II}), 5.29 – 5.15 (m, 3H, C-1^{III}), 5.10 br (s, 1H, C-1^{IV}), 5.03 (s, 2H), 4.97 – 4.73 (m, 5H), 4.60 – 4.36 (m, 7H), 4.32 – 4.22 (m, 3H, C-1^{II}, C-1^{I}), 4.19 – 4.05 (m, 5H), 3.98 (br s, 3H), 3.87 – 3.57 (m, 10H), 3.57 – 3.37 (m, 3H), 3.34 – 3.17 (m, 4H), 3.05 (d, *J* = 10.1 Hz, 1H), 2.34 (d, *J* = 10.0 Hz, 1H), 1.75 – 1.54 (m,

4H), 1.41 – 1.32 (m, 2H); ^{13}C NMR (101 MHz, $CDCl_3$) δ 156.5, 156.0, 139.0, 138.4, 138.0, 137.8, 137.7, 137.5, 137.0, 136.5, 128.5, 128.3, 128.2 (2C), 128.0, 127.9 (2C), 127.7, 127.6, 127.5, 127.3, 101.5 (C-1^{II}), 100.9 (benzylidene CH^{III}), 100.8 (benzylidene CH^{II}), 99.5 (C-1^{IV}), 98.6 (C-1^{I}), 97.5 (C-1^{III}), 81.5, 77.9, 77.2, 75.0,74.8, 74.5, 73.6, 72.9, 69.1, 68.9, 67.6, 67.0, 66.8, 63.2, 50.2, 50.0, 46.8, 45.9, 29.1, 27.6, 26.9, 22.8; HRMS: m/z: calcd. for $C_{93}H_{101}N_7O_{21}$ $[M+Na]^+$: 1674.6948; found: 1674.6935.

***N*-Benzyl-*N*-benzyloxycarbonyl-5-aminopentyl *O*-(2-azido-4,6-*O*-benzylidene-2-deoxy-β-D-galactopyranosyl)-(1→4)-[*O*-2,3,4,6-tetra-*O*-benzyl-α-D-glucopyranosyl]-(1→2)-*O*-(3-benzyl-4,6-*O*-benzylidene-α-D-glucopyranosyl)-(1→3)-(methyl 2-azido-2-deoxy-β-D-mannopyranosyl uronate) (48)**

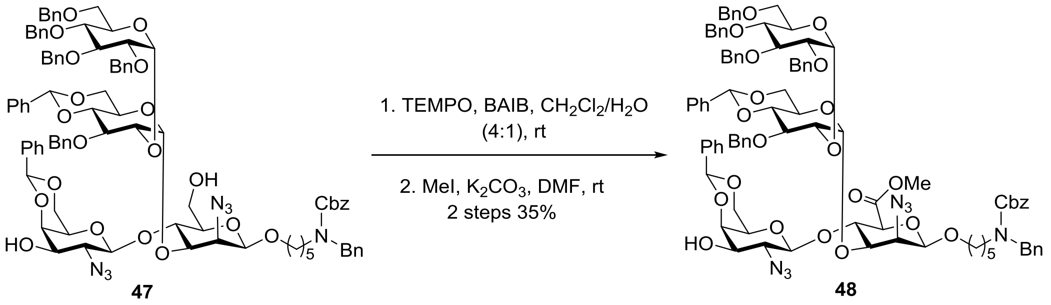

To a solution of **47** (400 mg, 0.242 mmol, 1.0 equiv) in a mixture of CH_2Cl_2/H_2O (4:1) (8 mL) at room temperature was added BAIB (195 mg, 0.605 mmol, 2.5 equiv) followed by TEMPO (19 mg, 72.60 μmol, 0.3 equiv). After stirring for 4 h, the organic phase was diluted with CH_2Cl_2 (25 mL) and the reaction mixture was quenched by the addition of 10% $Na_2S_2O_3$ (25 mL) and brine (25 mL). The organic phase was dried over anhydrous Na_2SO_4, filtered and the filtrate was concentrated in vacuo to give the corresponding crude carboxylic acid. HRMS: m/z: calcd. for $C_{93}H_{99}N_7O_{22}$ $[M+Na]^+$: 1688.6741; found: 1688.6716. To a solution of the carboxylic acid in DMF (5 mL) at room temperature was added K_2CO_3 (33 mg, 0.239 mmol) followed by methyl iodide (11 μL, 0.179 mmol, 1.5 equiv). After stirring for 4 h, the reaction was diluted with EtOAc (25 mL) and washed with brine (25 mL). The organic phase was dried over anhydrous Na_2SO_4, filtered and the filtrate was concentrated in vacuo. The

resulting dark yellow oil was purified by flash column chromatography over silica gel (EtOAc/hexanes, 1/1, v/v) to give **48** (141 mg, 35%) as a colorless solid: R_f = 0.5 (EtOAc/hexanes, 1/1, v/v); $[\alpha]_D^{20}$ = +10.3° (c = 0.25, $CHCl_3$); IR (thin film): ν_{max} = 667, 699, 746, 968, 1008, 1028, 1046, 1081, 1155, 1185, 1214, 1280, 1363, 1399, 1454, 1495, 1699, 1750, 2112, 2856, 2925; ^{1}H NMR (400 MHz, $CDCl_3$) δ 7.63 – 7.48 (m, 4H), 7.45 – 7.15 (m, 41H), 5.57 (s, 1H), 5.44 (s, 1H), 5.17 (d, *J* = 12.4 Hz, 2H), 5.15 (d, *J* = 3.2 Hz, 2H), 4.99 (q, *J* = 11.2 Hz, 2H), 4.86 (s, 2H), 4.80 (d, *J* = 11.0 Hz, 1H), 4.68 (d, *J* = 10.6 Hz, 2H), 4.51 (d, *J* = 7.3 Hz, 2H), 4.42 (d, *J* = 10.7 Hz, 2H), 4.40 – 4.35 (m, 1H), 4.29 (d, *J* = 12.2 Hz, 2H), 4.26 – 4.18 (m, 3H), 4.11 (t, *J* = 9.0 Hz, 2H), 3.96 (d, *J* = 10.0 Hz, 1H), 3.89 (s, 1H), 3.89 – 3.83 (m, 2H), 3.79 (s, 3H), 3.75 (d, *J* = 9.8 Hz, 2H), 3.71 – 3.66 (m, 2H), 3.65 – 3.57 (m, 4H), 3.55 (br s, 1H), 3.51 – 3.40 (m, 2H), 3.38 (s, 1H), 3.35 – 3.15 (m, 4H), 3.06 (d, *J* = 10.2 Hz, 1H), 2.88 (d, *J* = 10.7 Hz, 1H), 1.60 (br m, 4H), 1.29 (br m, 2H); HRMS: m/z: calcd. for $C_{94}H_{101}N_7O_{22}$ $[M+Na]^+$: 1702.6897; found: 1702.6933.

***N*-Benzyl-*N*-benzyloxycarbonyl-5-aminopentyl *O*-(3,4-di-*O*-acetyl-2-azido-2,6-dideoxy-α-L-fucopyranosyl)-(1→3)-*O*-(2-azido-4,6-*O*-benzylidene-2-deoxy-β-D-galactopyranosyl)-(1→4)-[*O*-2,3,4,6-tri-*O*-benzyl-α-D-glucopyranosyl]-(1→2)-(3-*O*-benzyl-4,6-*O*-benzylidene-*α*-D-glucopyranosyl)-(1→3)-(methyl 2-azido-2-deoxy-β-D-mannopyranosyl uronate) (49)**

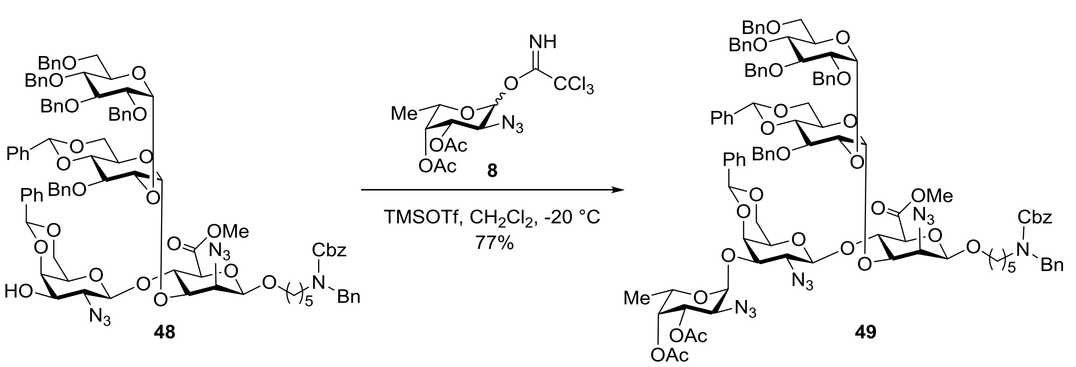

A mixture of fucosyl donor **8** (104 mg, 0.249 mmol, 3.0 equiv) and tetrasaccharide acceptor **48** (140 mg, 0.0832 mmol, 1.0 equiv) in CH_2Cl_2 (5 mL) was stirred under an atmosphere of

Ar for 30 min. The reaction mixture was cooled (−20 °C) and TMSOTf (4.5 µL, 24.987 µmol, 0.3 equiv) was added. After stirring for 45 min, the reaction mixture was quenched by the addition of Et_3N (0.5 mL) and concentrated in vacuo. The resulting dark yellow oil was purified by flash column chromatography over silica gel (EtOAc/hexanes, 2/3, v/v) to give **49** (125 mg, 77%) as a colorless solid. R_f = 0.4 (EtOAc/hexanes, 4/6, v/v); $[\alpha]_D^{20}$ = +2.2° (c = 0.5, $CHCl_3$); IR (thin film): ν_{max} = 699, 750, 764, 992, 1048, 1073, 1234, 1256, 1276, 1369, 1454, 1496, 1694, 1726, 1751, 2115, 2869, 2929; ^1H NMR (400 MHz, $CDCl_3$) δ 7.69 – 6.83 (m, 45H), 5.54 (s, 1H), 5.38 – 5.28 (m, 3H), 5.23 – 5.03 (m, 5H), 4.92 (s, 2H), 4.84 – 4.72 (m, 4H), 4.56 (d, J = 10.3 Hz, 1H), 4.52 – 4.42 (m, 3H), 4.41 – 4.23 (m, 6H), 4.20 – 4.01 (m, 7H), 3.98 (br s, 2H), 3.87 – 3.77 (m, 7H), 3.74 – 3.63 (m, 4H), 3.62 – 3.55 (m, 2H), 3.39 – 3.10 (m, 6H), 2.96 (d, J = 9.5 Hz, 1H), 2.13 (s, 3H), 2.03 (s, 3H), 1.53 (br s, 4H), 1.26 (br s, 2H), 0.94 (br s, 3H); ^{13}C NMR (101 MHz, $CDCl_3$) δ 170.2, 169.5, 168.4, 156.6, 156.1, 138.9, 138.6, 138.4, 138.3, 137.8, 137.5, 137.1, 128.4, 128.2 (2C), 128.1 (2C), 128.0, 127.9, 127.7, 127.6, 127.5, 125.9, 102.7 (C-1^{II}), 101.0 (benzylidene $CH^{II,\ III}$), 100.3 (C-1^{IV}), 99.5 (C-1^{I}), 98.0 (C-1^{III}), 96.1 (C-1^{V}), 77.6, 76.9, 75.0, 74.9, 74.7 (2C), 72.9, 70.3, 68.5, 67.0, 65.4, 61.9, 57.4, 52.7, 50.4, 50.1, 46.9, 46.1, 29.0, 27.8, 27.4, 23.0, 20.6 (fucose acetate), 20.5 (fucose acetate), 15.9 (fucose methyl); HRMS: m/z: calcd. for $C_{104}H_{114}N_{10}O_{27}$ $[M+Na]^+$: 1957.7753; found: 1957.7796.

***N*-Benzyl-*N*-benzyloxycarbonyl-5-aminopentyl *O*-(4-*O*-benzoyl-2,3,6-tri-*O*-benzyl-α-D-galactopyranosyl)-(1→3)-*O*-(4-*O*-acetyl-2-azido-2,6-dideoxy-α-L-fucopyranosyl)-(1→3)-*O*-(2-azido-4,6-*O*-benzylidene-2-deoxy-β-D-galactopyranosyl)-(1→4)-[*O*-2,3,4,6-tetra-*O*-benzyl-α-D-glucopyranosyl]-(1→2)-*O*-(3-*O*-benzyl-4,6-*O*-benzylidene-α-D-glucopyranosyl)-(1→3)-(methyl 2-azido-2-deoxy-β-D-mannopyranosyl uronate) (51)**

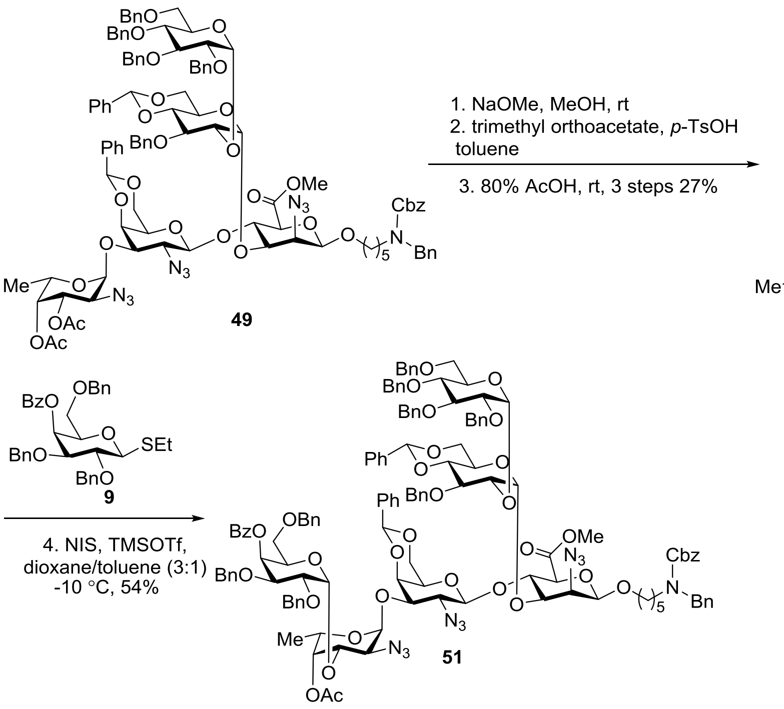

To a solution of **49** (60 mg, 30.99 μmol, 1.0 equiv) in a mixture of THF/MeOH (1:1) (2 mL) at room temperature was added 0.5 M NaOMe in MeOH (31 μL, 15.495 μmol, 0.5 equiv). After stirring for 12 h, the reaction mixture was neutralized by the addition of Amberlite 120 (H^+) resin, filtered and the filtrate was concentrated in vacuo. To the residue was added toluene (2 mL) followed by 1,1,1-trimethoxy ethane (78 μL, 0.647 mmol, 20.0 equiv) and *p*-TsOH (1.20 mg, 6.47 μmol) at room temperature. After stirring for 2 h, the reaction mixture was quenched by the addition of Et_3N (0.5 mL). The mixture was concentrated in vacuo. To the crude residue was added 80% AcOH (2 mL) and the reaction mixture was stirred for 60 min at room temperature. Acetic acid was co-evaporated with toluene and the residue was purified by flash column chromatography over silica gel (EtOAc/hexanes, 1/1, v/v) to give **50** (16 mg, 27%) as a colorless solid. R_f = 0.4 (EtOAc/hexanes, 1/1, v/v); HRMS: m/z: calcd. for

$C_{102}H_{112}N_{10}O_{26}$ $[M+Na]^+$: 1915.7647; found: 1915.7611. A mixture of the galactosyl donor **9** (19 mg, 31.678 μmol, 2.0 equiv) and pentasaccharide acceptor **50** (30 mg, 15.839 μmol, 1.0 equiv) in a mixture of toluene/dioxane (3:1, 1.5 mL) was stirred under an atmosphere of Ar for 30 min. The reaction mixture was cooled (−10 °C) and NIS (7.1 mg, 31.678 μmol, 2.0 equiv) was added followed by the addition of TMSOTf (0.8 μL, 4.751 μmol, 0.3 equiv). After stirring for 45 min, warmed to 0 °C, the reaction mixture was quenched by the addition of Et_3N (0.25 mL). The mixture was diluted with EtOAc (25 mL) and the organic layer washed with 10% $Na_2S_2O_3$ (25 mL) and brine (25 mL). The organic phase was dried over anhydrous Na_2SO_4, filtered and the filtrate was concentrated in vacuo. The resulting dark yellow oil was purified by flash column chromatography over silica gel (EtOAc/hexanes, 2/3, v/v) to give **51** (21 mg, 54%) as a colorless solid: R_f = 0.5 (EtOAc/hexanes, 3/7, v/v). ^1H NMR (400 MHz, $CDCl_3$) δ 8.05 – 7.88 (m, 2H), 7.49 – 7.07 (m, 63H), 5.82 (s, 1H), 5.53 (s, 1H, benzylidene CH^{III}), 5.37 – 5.29 (m, 2H, C-1^{V}), 5.24 (s, 1H, benzylidene CH^{II}), 5.21 – 5.08 (m, 4H, C-1^{III}, C-1^{IV}, C-1^{VI}), 4.91 (d, *J* = 5.9 Hz, 2H), 4.84 – 4.72 (m, 7H), 4.68 (s, 2H), 4.59 – 4.54 (m, 2H), 4.54 – 4.43 (m, 4H, C-1^{II}), 4.41 – 4.33 (m, 4H), 4.30 (d, *J* = 6.5 Hz, 2H), 4.27 – 4.22 (m, 2H), 4.17 – 4.05 (m, 6H, C-1^{I}), 3.98 – 3.94 (m, 3H), 3.89 – 3.82 (m, 3H), 3.80 (s, 3H), 3.76 – 3.64 (m, 4H), 3.62 – 3.53 (m, 3H), 3.44 (d, *J* = 6.1 Hz, 2H), 3.38 – 3.31 (s, 2H), 3.27 3.15 (m, 6H), 3.02 – 2.90 (m, 1H), 2.03 (s, 3H), 1.53 – 1.46 (m, 4H), 1.36 – 1.28 (m, 2H), 0.87 (d, *J* = 6.8 Hz, 3H) ; ^{13}C NMR (101 MHz, $CDCl_3$) δ 177.8, 171.0, 165.6, 156.6, 156.1, 138.9, 138.7, 138.3, 138.2, 138.1, 137.9, 137.8, 137.6, 137.5, 137.3, 132.9, 129.9, 129.7, 128.4, 128.3, 128.2 (2C), 128.1, 128.0, 127.9, 127.8, 127.7 (2C), 127.4, 102.6 (C-1^{II}), 101.0 (benzylidene CH^{III}), 101.0 (benzylidene CH^{II}), 100.4 (C-1^{IV}), 99.5 (C-1^{I}), 99.3 (C-1^{III}), 97.9 (C-1^{VI}), 96.1 (C-1^{V}), 82.7, 81.9, 80.4, 80.1, 77.6, 77.2, 76.9, 76.6, 75.6, 75.0, 74.9, 74.8 (2C), 74.6, 74.0, 73.9, 73.7, 73.4, 73.0, 72.9, 72.7, 72.3, 71.7, 71.4, 70.6, 69.7, 69.2, 69.0, 67.8, 67.0, 66.8, 66.2, 63.4, 61.9, 60.2, 52.7, 50.4, 50.1, 47.0, 46.0, 29.0, 28.1, 27.7, 27.3,

23.0, 20.7 (fucose acetate), 16.1 (fucose methyl); LCMS: m/z: calcd. for $C_{136}H_{144}N_{10}O_{32}$ $[M+Na]^+$: 2451.9; found: 2451.9.

5-Aminopentyl *O*-(*α*-D-galactopyranosyl)-(1→3)-*O*-(α-L-fucopyranosyl)-(1→3)-*O*-(β-D-galactopyranosyl)-(1→4)-[*O*-α-D-glucopyranosyl]-(1→2)-*O*-(α-D-glucopyranosyl)-(1→3)-β-D-mannopyranosyl uronic acid (1)

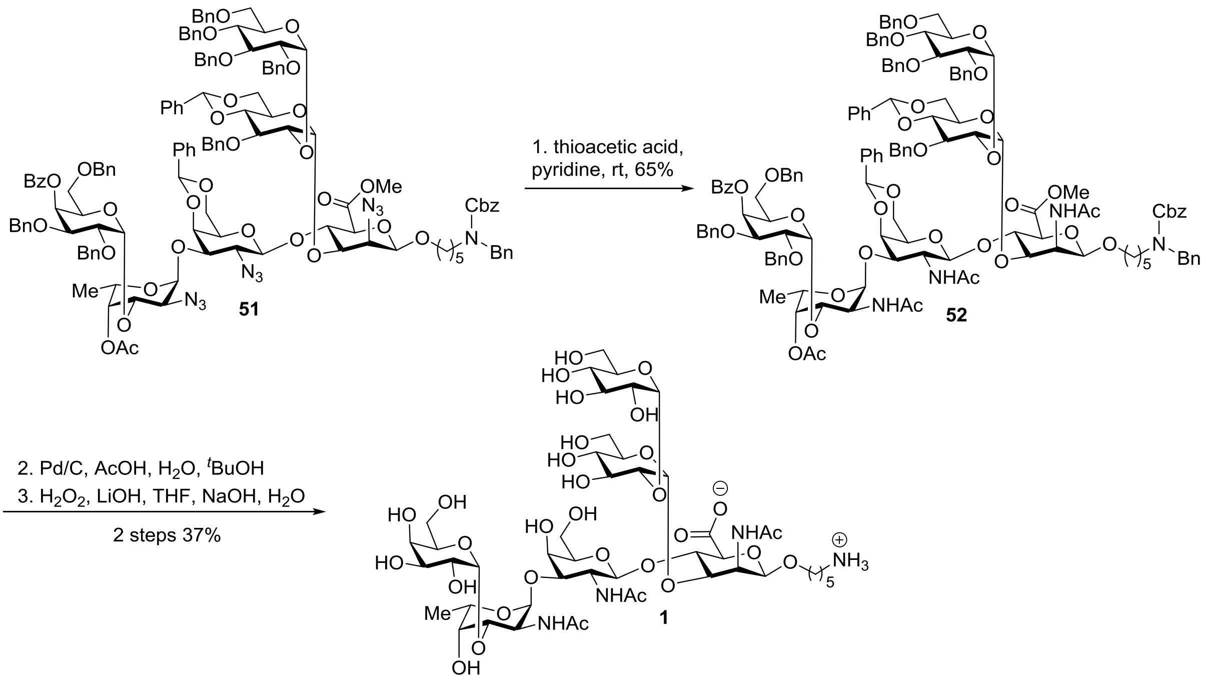

To a solution of **51** (6.5 mg, 3.431 μmol, 1.0 equiv) in pyridine (2 mL) was added thioacetic acid (0.25 mL) at room temperature. After stirring for 120 h, the reaction mixture was concentrated and passed through a short silica gel column using ethyl acetate as eluent and concentrated in vacuo to give **52**. ^{1}H NMR (400 MHz, $CDCl_3$) δ 8.00 (m, 2H, *ortho*-H of benzoate), 7.48–7.07 (m, 63H), 5.87 (d, J = 2.6 Hz, 1H), 5.50 (s, 1H), 5.27 (m, 1H), 5.16 (m, 6H), 4.99 – 4.83 (m, 7H), 4.72 – 4.56 (m, 5H), 4.47 – 4.32 (m, 12H), 4.22 – 4.11 (m, 9H), 3.96 – 3.82 (m, 13H), 3.71 (s, 3H, Me-ester), 3.64 – 3.55 (m, 8H), 3.41 (m, 3H), 3.31 (m, 4H), 3.16 (m, 2H), 2.03 (s, 3H, NHAc), 1.81 (s, 3H, NHAc), 1.63 (s, 3H, NHAc), 1.34 – 1.25 (m, 4H), 1.16 – 1.07 (m, 2H), 1.03 (d, J = 6.5 Hz, 3H, fucose-methyl); ^{13}C NMR (101 MHz, $CDCl_3$) δ 171.0, 170.4, 169.2, 165.4, 138.8, 138.5, 138.4, 138.3, 138.0, 137.9, 137.8, 137.7,

137.6, 129.7, 128.6 (2C), 128.5, 128.4, 128.3, 128.2 (2C), 128.1, 128.0 (2C), 127.8, 127.7 (2C), 127.5 (2C), 127.2, 126.1, 125.9, 101.2, 101.1, 100.1, 99.6, 99.1, 98.0, 96.3, 95.7, 82.6, 81.6, 79.2, 77.6, 77.2, 77.1, 76.6, 76.2, 75.6, 75.3, 75.1, 74.8, 74.5, 74.0, 73.8, 73.4, 73.3, 71.6, 70.6, 70.5, 69.8, 69.2, 68.7, 68.5, 67.8, 67.8, 67.1, 67.0, 66.2, 65.4, 63.1, 54.2, 51.8, 50.1, 49.8, 46.1, 44.9, 29.6, 27.6, 27.2, 23.3, 23.2, 23.0, 22.9, 16.6; MALDI (TOF): m/z: calcd. for $C_{142}H_{158}N_0O_{35}$ $[M+Na+H]^+$: 2501.0526; found: 2501.0153; To the residue was added ^{t}BuOH/AcOH/H_2O (25:1:1, 2 mL) followed by $Pd(OH)_2/C$ (20 mg, 20% wt.%) at room temperature. After stirring for 24 h under an atmosphere of H_2, the reaction mixture was diluted with ^{t}BuOH/AcOH/H_2O (25:1:1, 10 mL) and filtered through celite. The mixture was concentrated in vacuo. The residue was dissolved in water (1 mL) followed by addition of premixed solution of hydrogen peroxide (0.5 mL, 30% in H_2O) and 1M solution of LiOH (1 mL). The reaction mixture was stirred for 120 min at room temperature and 1M NaOH (1 mL) solution was added. The mixture was stirred for 4 h and neutralized using Amberlite 120 (H^+) resin. The solution was diluted with water (5 mL), filtered and concentrated in vacuo. The residue was purified by size-exclusion chromatography using water as eluent to give **1** (1.2 mg, 37%) as a colorless solid. ^{1}H NMR (600 MHz, D_2O) δ 5.30 (d, *J* = 3.6 Hz, 1H, (C-1^{V}), 5.20 (d, *J* = 3.4 Hz, 1H, (C-1^{VI})), 4.93 (d, *J* = 4.2 Hz, 1H, (C-1^{IV}), 4.85 (d, *J* = 3.9 Hz, 1H, (C-1^{III}), 4.51 (d, *J* = 8.6 Hz, 1H, (C-1^{II}), 4.40 (d, *J* = 3.6 Hz, 1H), 4.23 – 4.21 (m, 1H), 4.19 – 4.17 (m, 1H), 4.00 – 3.97 (m, 2H), 3.91 – 3.88 (m, 2H), 3.85 – 3.83 (m, 2H), 3.76 – 3.73 (m, 4H), 3.71 – 3.70 (m, 2H), 3.68 – 3.66 (m, 2H), 3.62 – 3.60 (m, 5H), 3.55 – 3.51 (m, 3H), 3.49 – 3.46 (m, 1H), 3.37 – 3.31 (m, 2H), 2.85 (t, *J* = 7.2 Hz, 2H), 1.94 (s, 3H, NHAc), 1.89 (s, 3H, NHAc),1.78 (s, 3H, NHAc), 1.53 (t, *J* = 7.8 Hz, 2H), 1.47 (t, *J* = 7.2 Hz, 2H), 1.27 (m, 2H), 1.09 (d, *J* = 6.5 Hz, 3H, fucose-methyl); ^{13}C NMR (HSQCAD, 150 MHz, D_2O): δ 103.4 (C-1^{IV}), 101.7 (C-1^{III}), 101.5 (C-1^{I}), 100.6 (C-1^{II}), 98.4 (C-1^{V}), 97.4 (C-1^{VI}), 80.6, 79.8, 79.1, 78.6, 78.3, 77.8, 76.9, 76.7, 76.5, 75.3, 74.8, 74.7 (2C), 73.7, 72.4, 72.2, 72.1 (2C), 72.0, 71.9, 71.8, 71.6, 71.0, 70.2, 69.7, 64.0, 63.4, 63.3, 63.2, 63.0, 54.6, 50.9, 50.7, 41.9, 30.9, 28.8,

25.9, 25.0, 24.9, 24.7, 18.1 (fucose methyl); HRMS: m/z: calcd. for $C_{47}H_{80}N_4O_{31}$ $[M+Na]^+$: 1219.4704; found: 1219.4672.

NMR spectra of new compounds

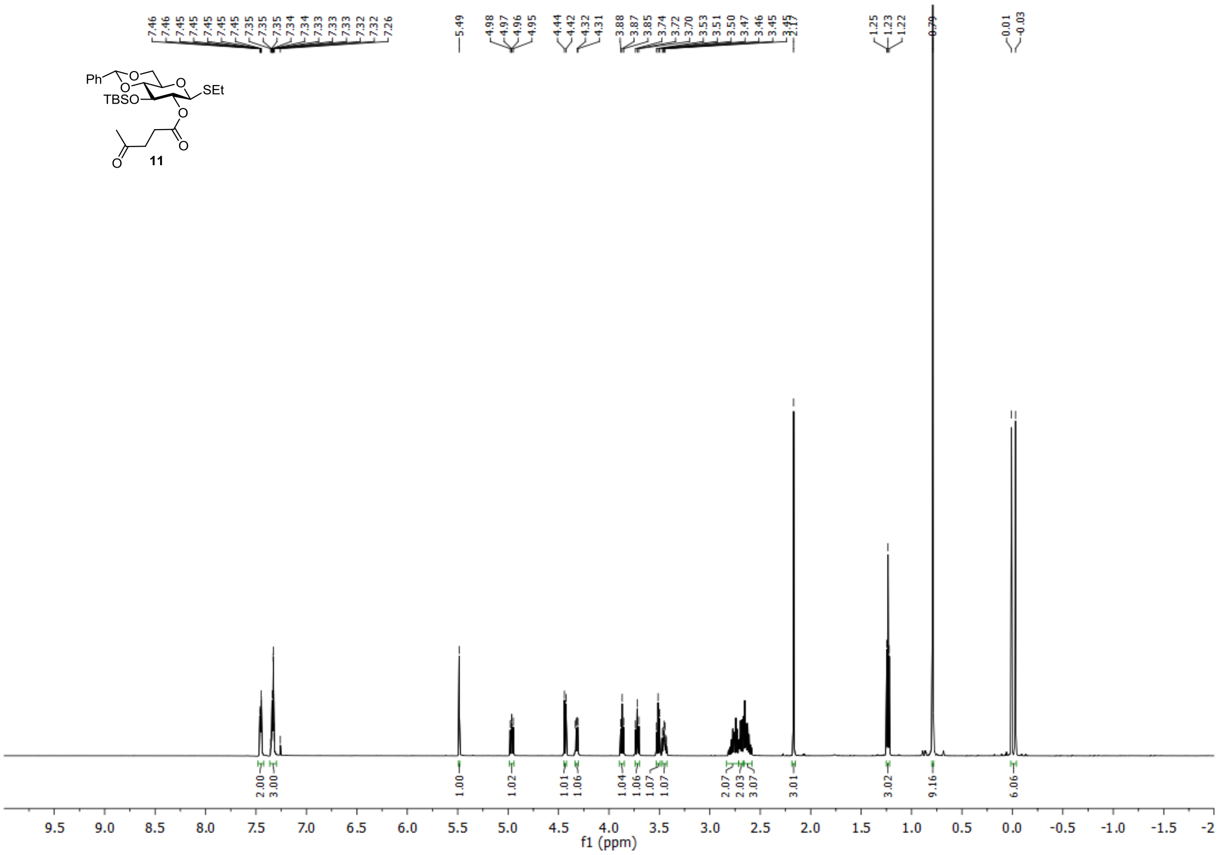

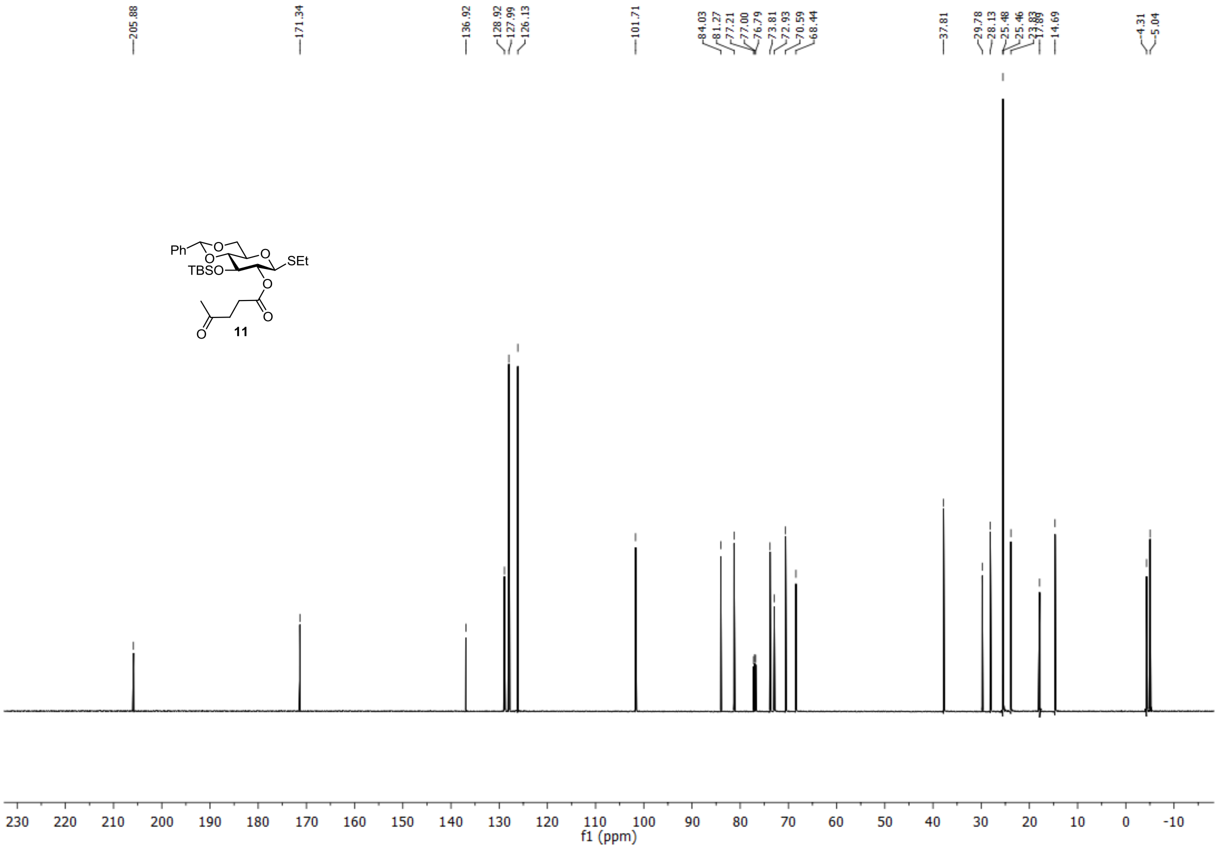

205.88
171.34
136.92
128.92
127.99
126.13
101.71
84.03
81.27
77.21
77.00
76.79
73.81
72.93
70.59
68.44
37.81
29.78
28.13
25.48
25.46
14.69
-4.31
-5.04
Ph
O
O
O
TBSO
SEt
O
O
O
11
230 220 210 200 190 180 170 160 150 140 130 120 110 100 90 80 70 60 50 40 30 20 10 0 -10
f1 (ppm)

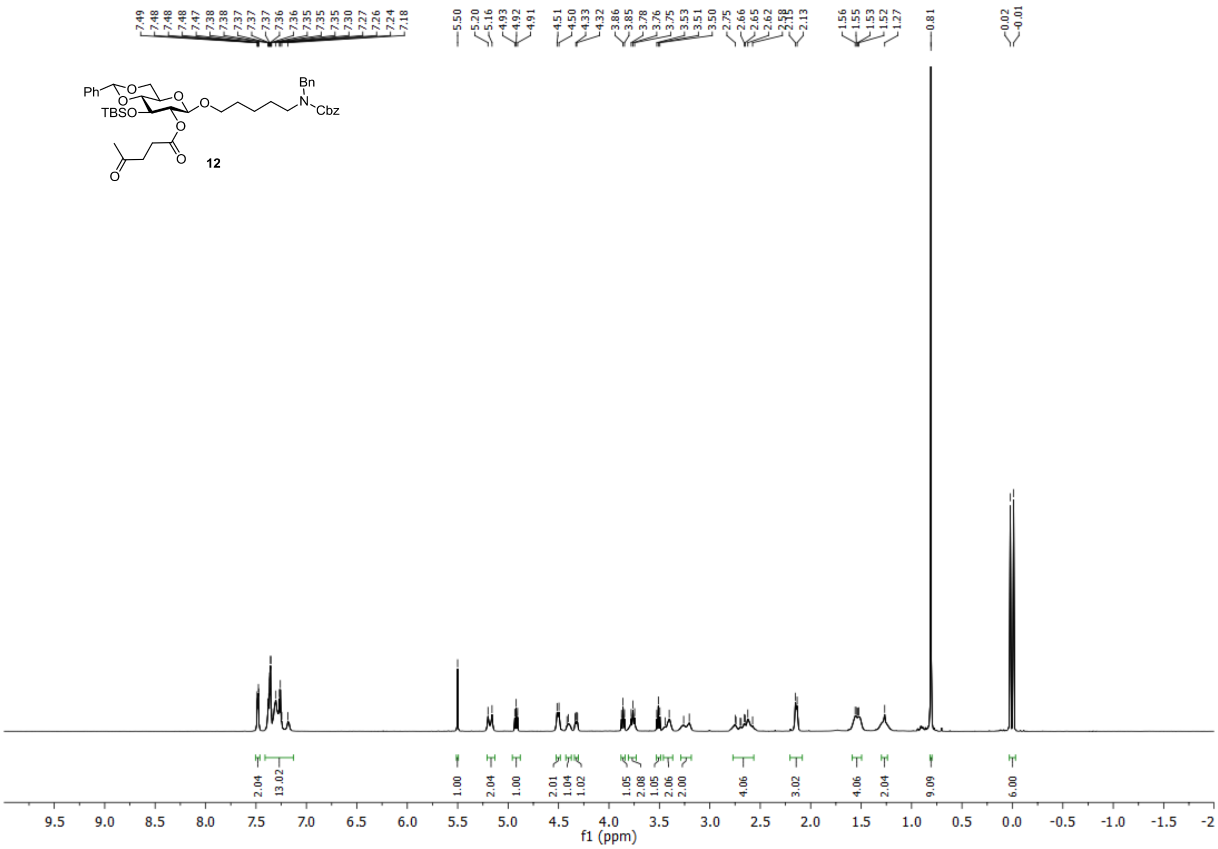

Ph
TBSO
Bn
N
Cbz
12
7.49 7.48 7.48 7.47 7.38 7.38 7.37 7.37 7.37 7.36 7.36 7.35 7.35 7.35 7.30 7.27 7.26 7.24 7.18
5.50 5.20 5.16 4.93 4.92 4.91 4.51 4.50 4.33 4.32
3.86 3.85 3.78 3.76 3.75 3.53 3.51 3.50
2.75 2.66 2.65 2.62 2.58 2.15 2.13
1.56 1.55 1.53 1.52 1.27 0.81 0.02 -0.01
2.04 13.02 1.00 2.04 1.00 2.01 1.04 1.02 1.05 2.08 1.05 2.06 2.00 4.06 3.02 4.06 2.04 9.09 6.00
f1 (ppm)

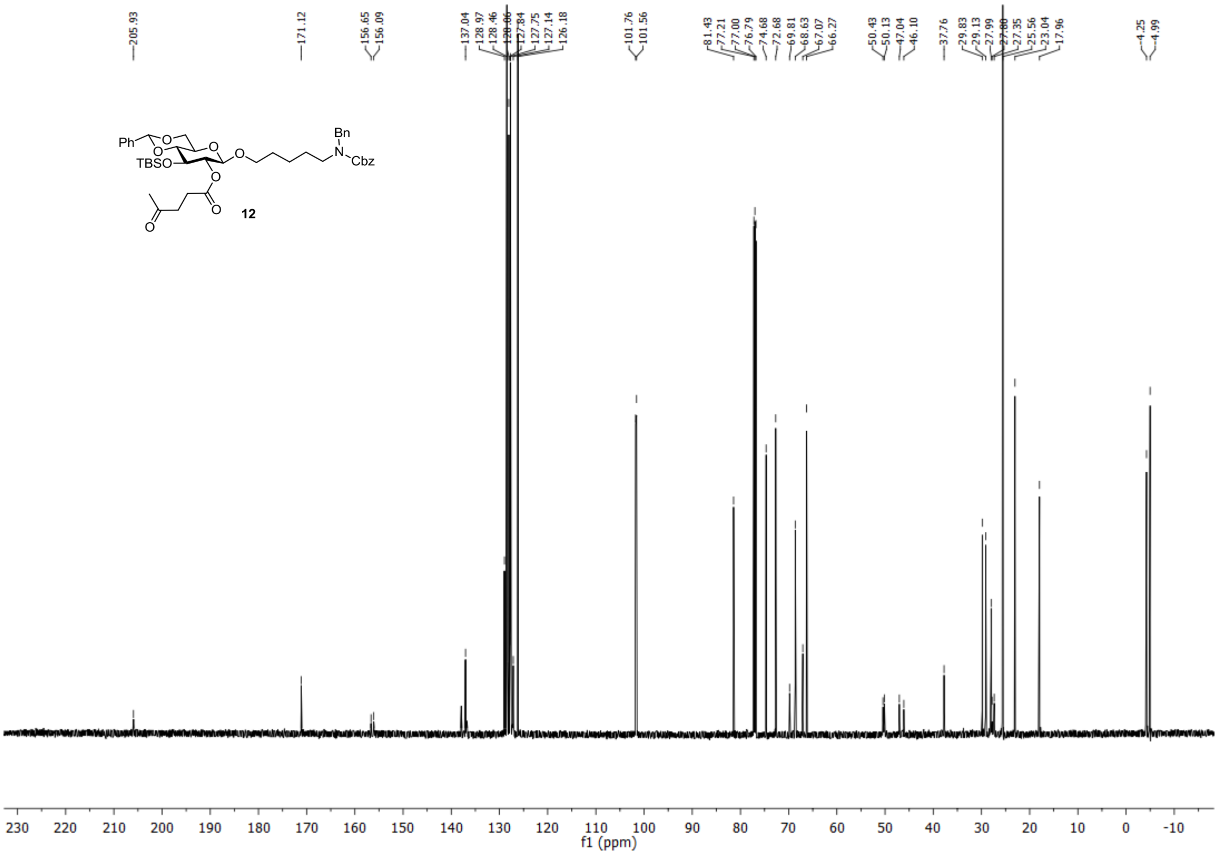

Ph
TBSO
Bn
N
Cbz
12
205.93 171.12 156.65 156.09
137.04 128.97 128.46 127.84 127.75 127.14 126.18
101.76 101.56
81.43 77.21 77.00 76.79 74.68 72.68 69.81 68.63 67.07 66.27
50.43 50.13 47.04 46.10 37.76 29.83 29.13 27.99 27.35 25.56 23.04 17.96
-4.25 -4.99
f1 (ppm)

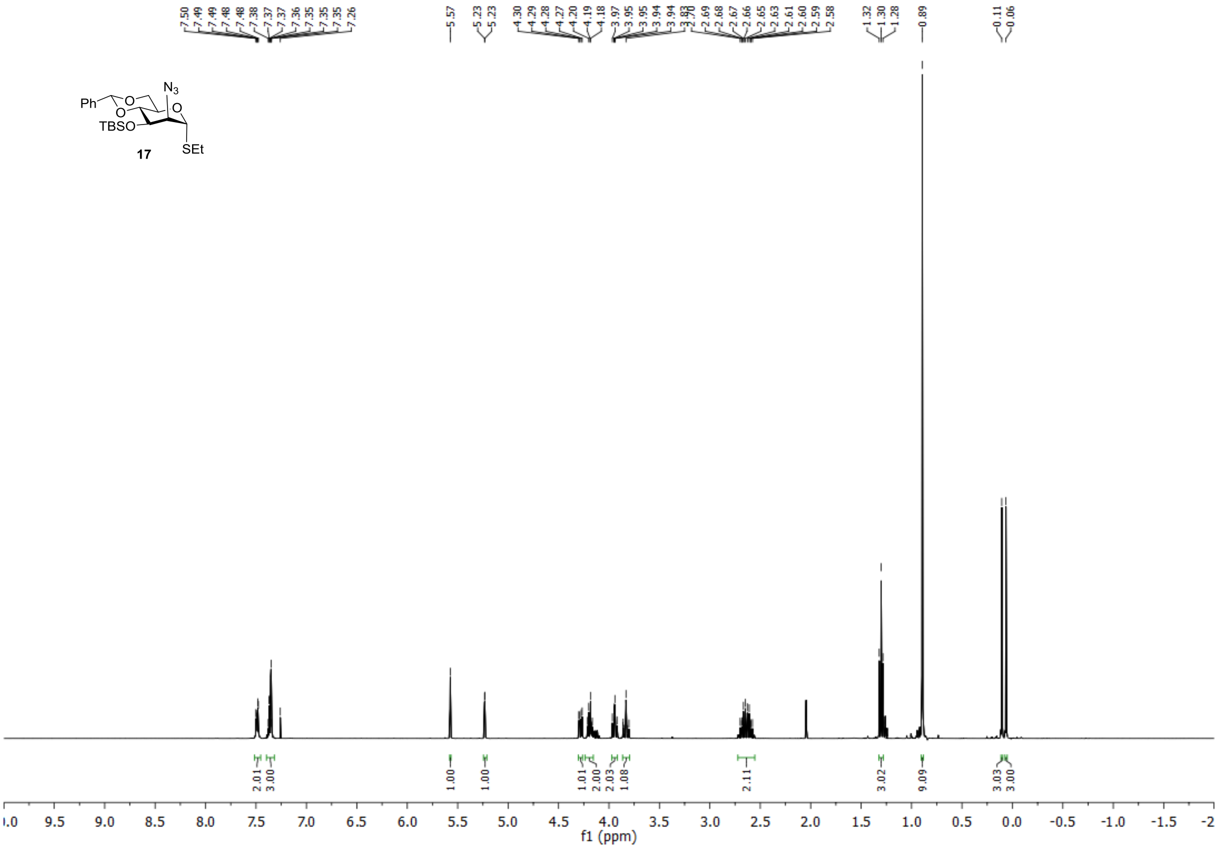
Ph
TBSO
N3
O
SEt
17
f1 (ppm)

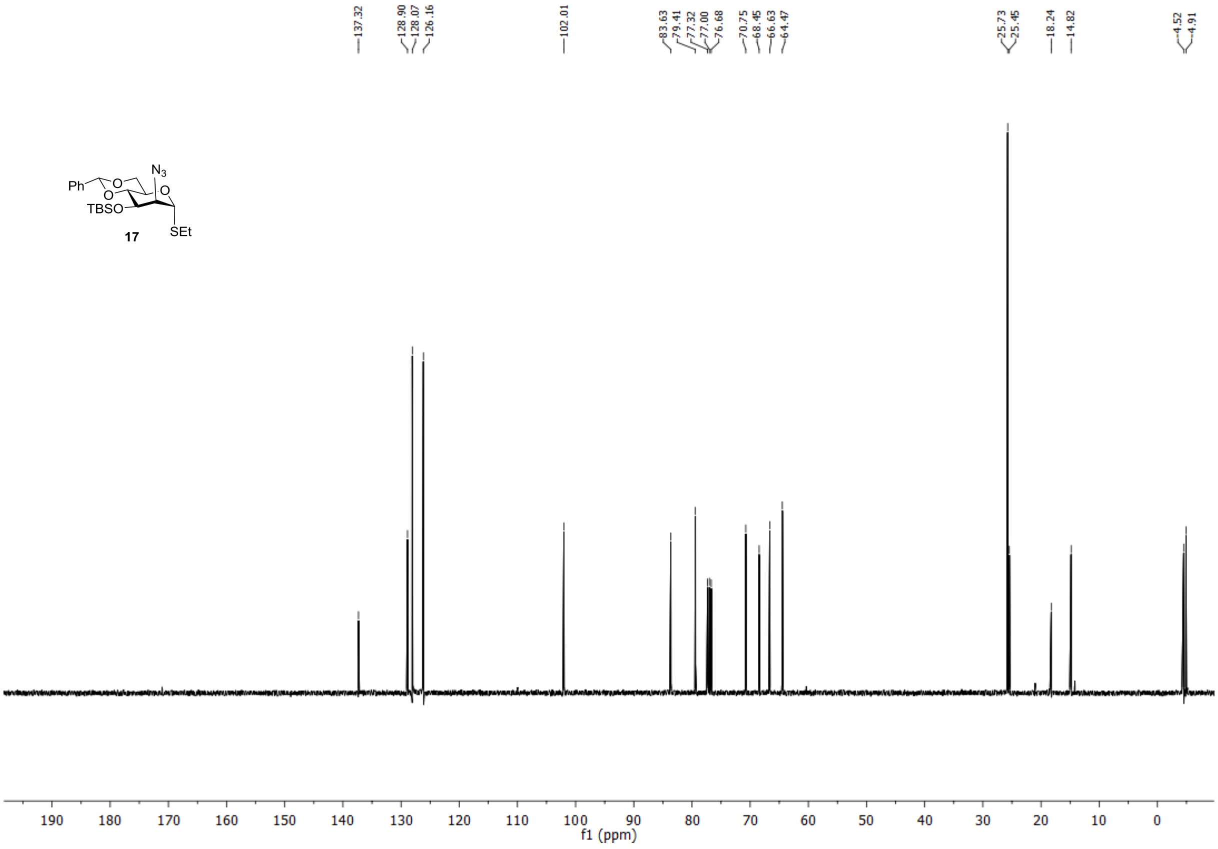
Ph
TBSO
N3
O
SEt
17
f1 (ppm)

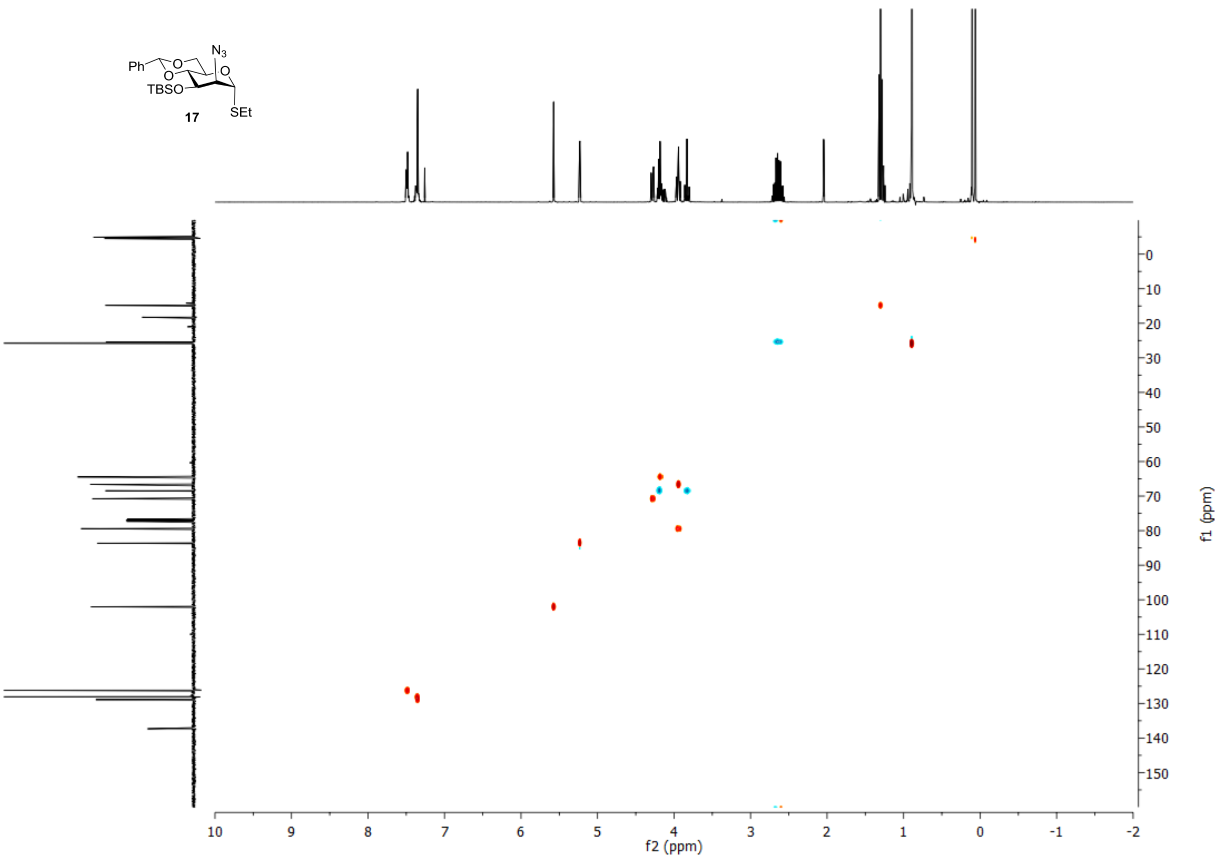

Ph
O
O
N3
O
TBSO
17
SEt
f2 (ppm)
f1 (ppm)

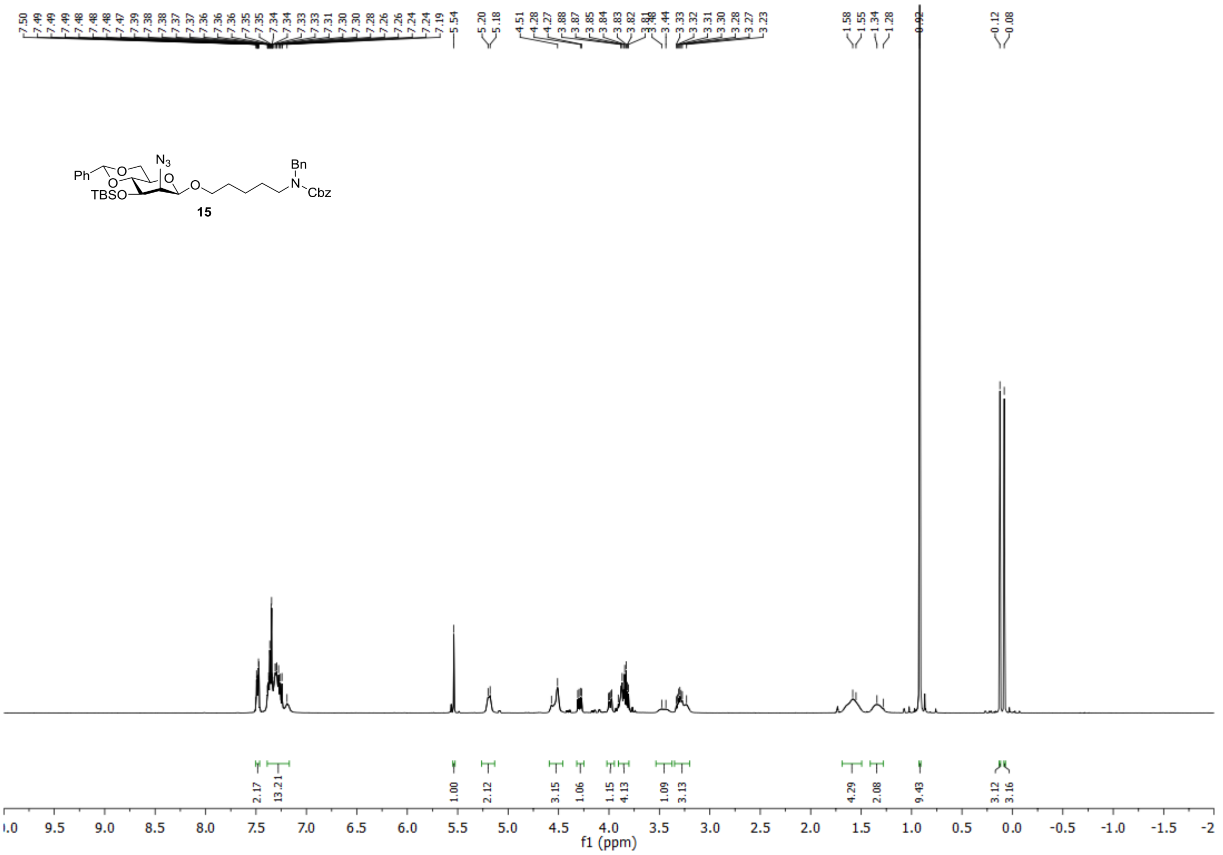

Ph
O
O
N3
O
O
Bn
N
Cbz
TBSO
15
f1 (ppm)

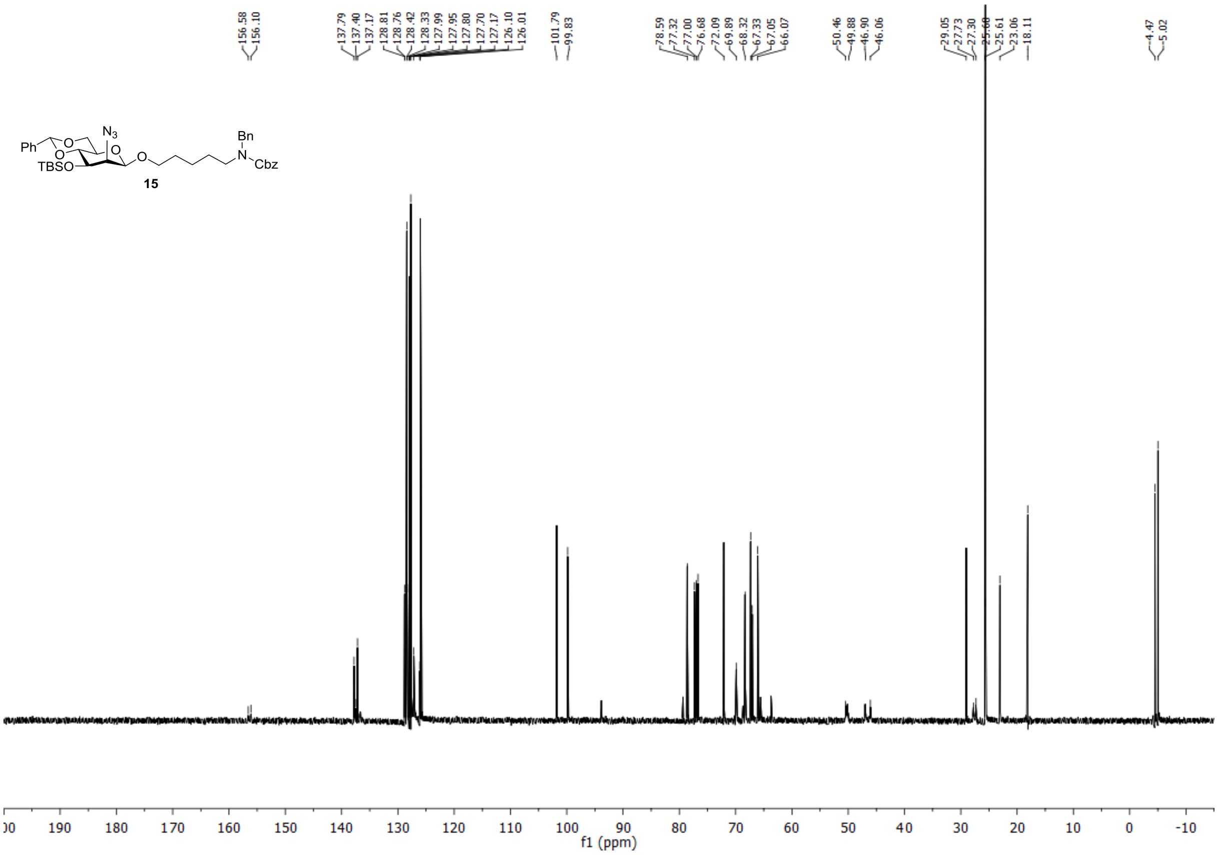

156.58
156.10
137.79
137.40
137.17
128.81
128.76
128.42
128.33
127.99
127.95
127.80
127.70
127.17
126.10
126.01
101.79
99.83
78.59
77.32
77.00
76.68
72.09
69.89
68.32
67.33
67.05
66.07
50.46
49.88
46.90
46.06
29.05
27.73
27.30
25.68
25.61
23.06
18.11
-4.47
-5.02
Ph
O
O
N3
O
O
Bn
N
Cbz
TBSO
15
f1 (ppm)

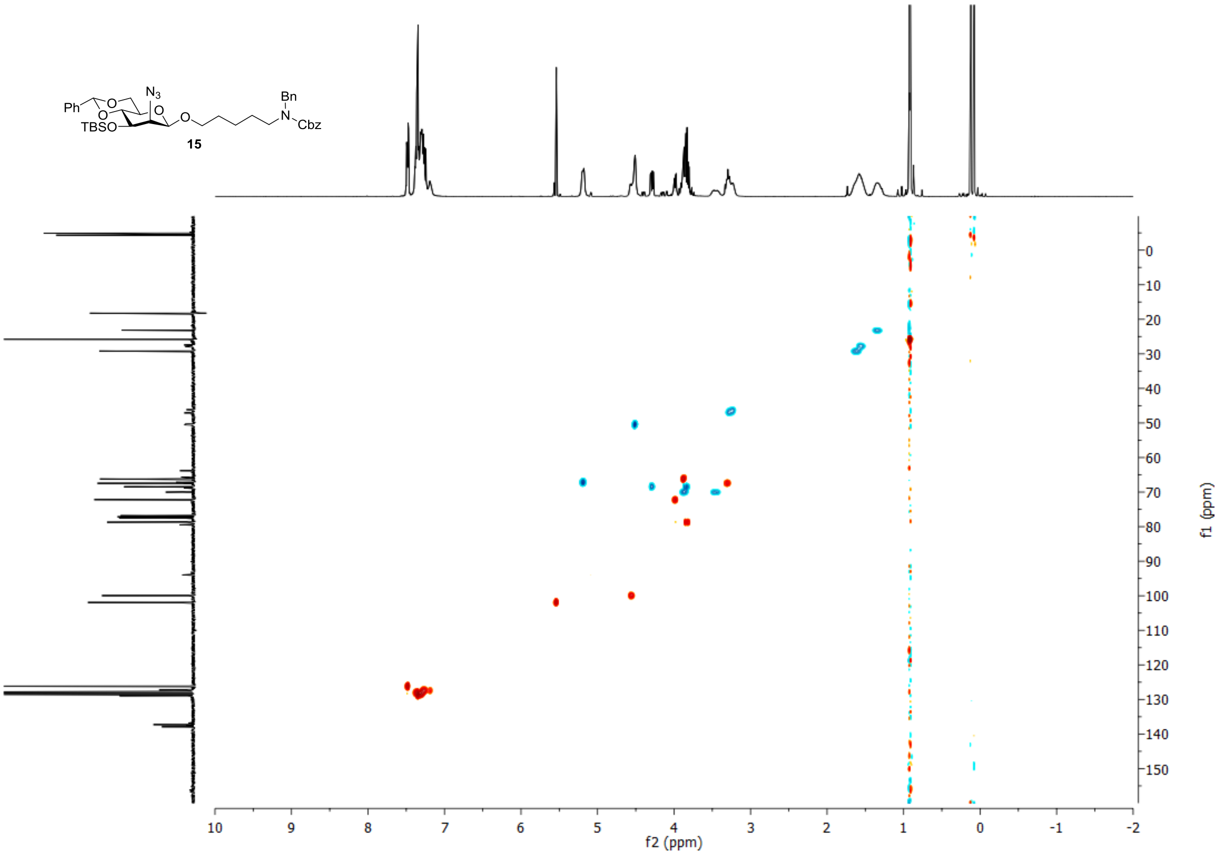

Ph
O
O
N3
O
O
Bn
N
Cbz
TBSO
15
f2 (ppm)
f1 (ppm)

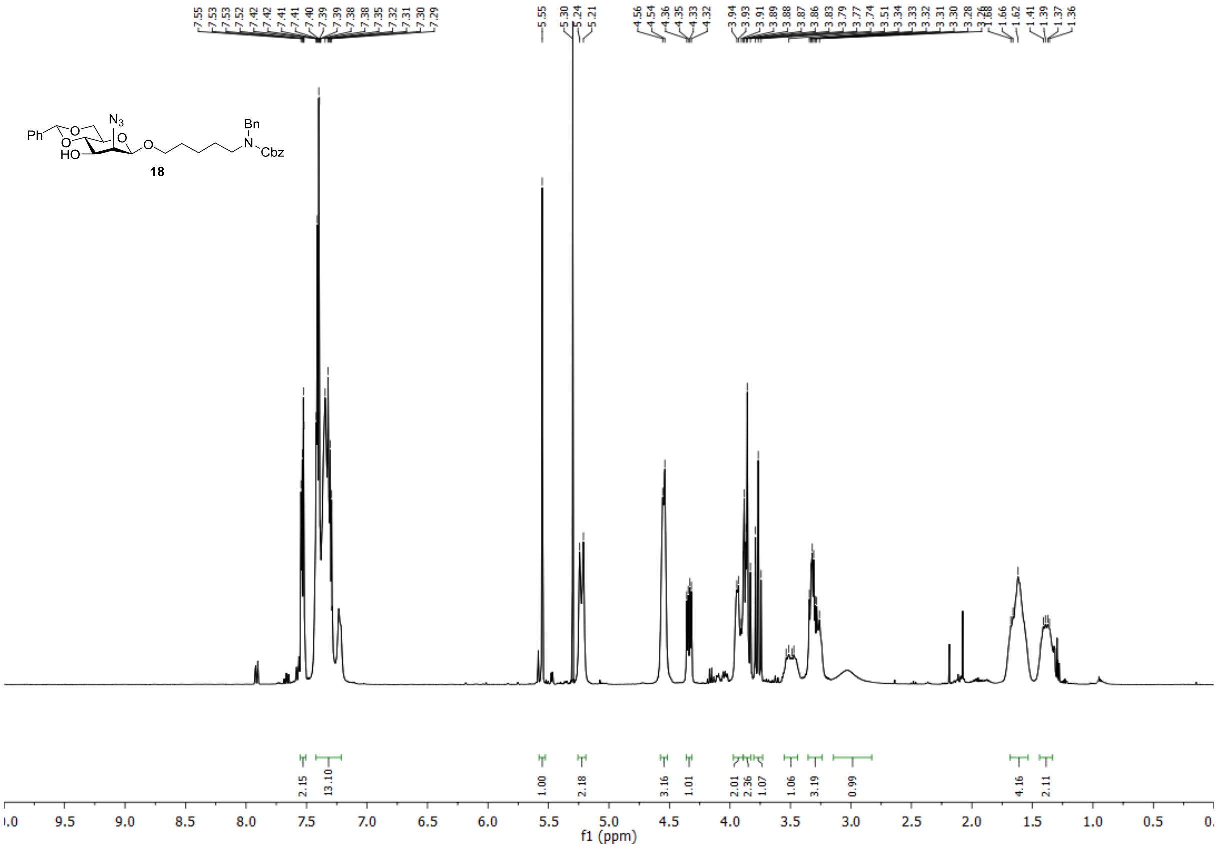

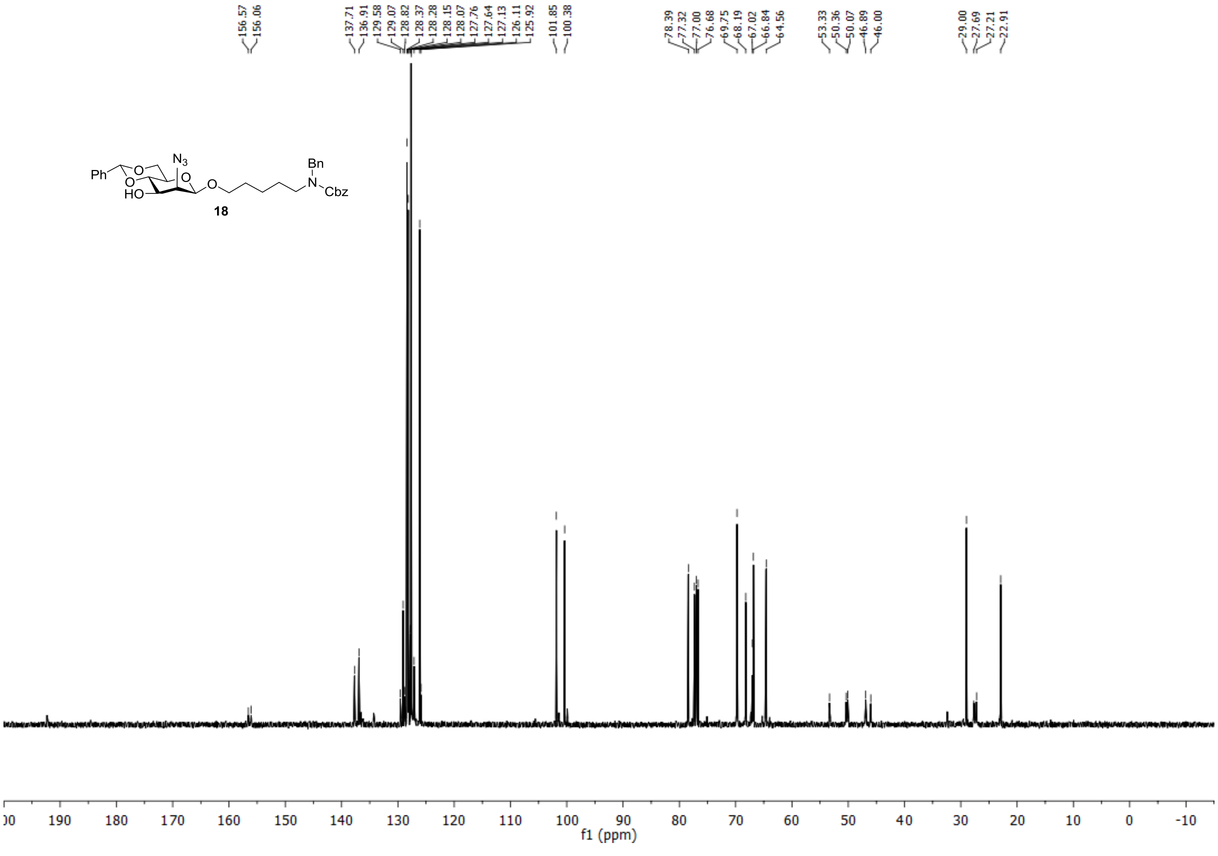

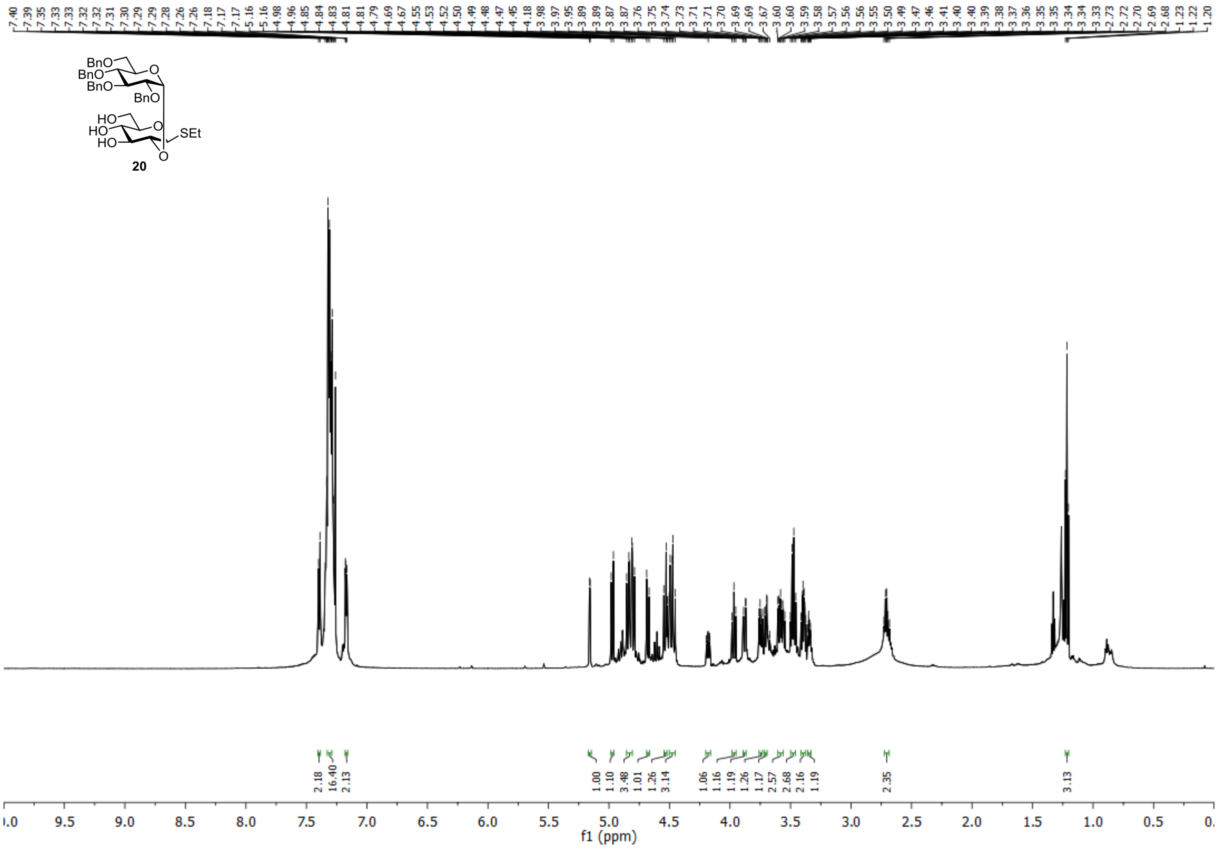
BnO
BnO
BnO
BnO
HO
HO
HO
SEt
O
20
f1 (ppm)

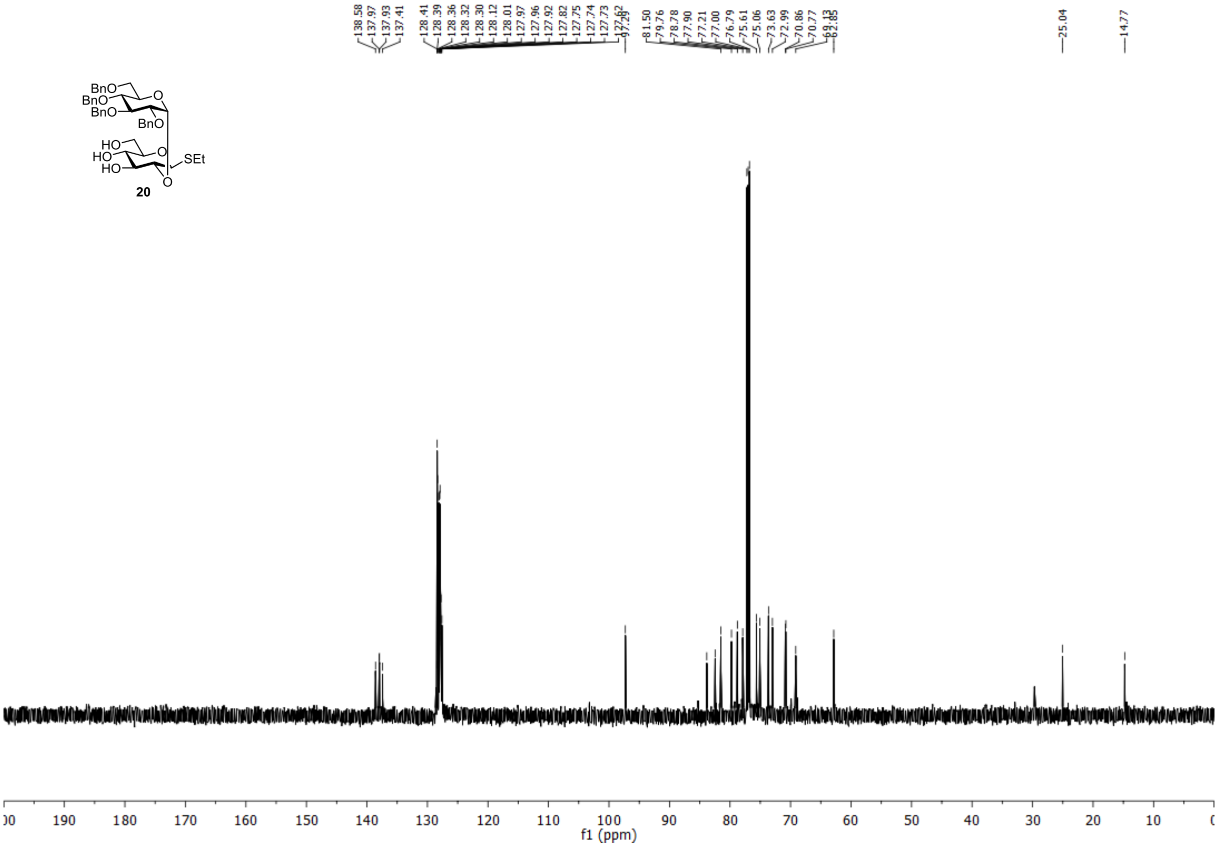
BnO
BnO
BnO
BnO
HO
HO
HO
SEt
O
20
f1 (ppm)

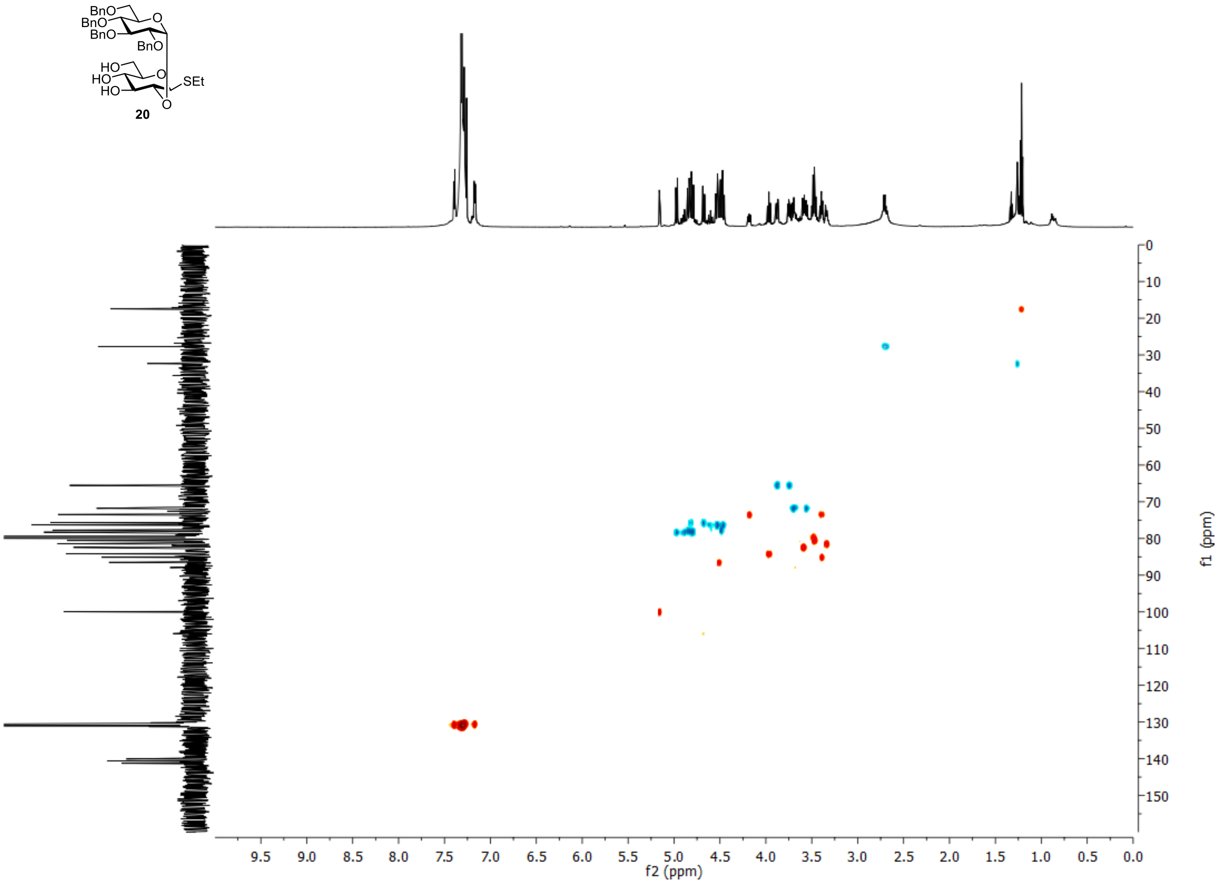

BnO
BnO
BnO
BnO
HO
HO
HO
O
O
O
SEt
20
f2 (ppm)
f1 (ppm)

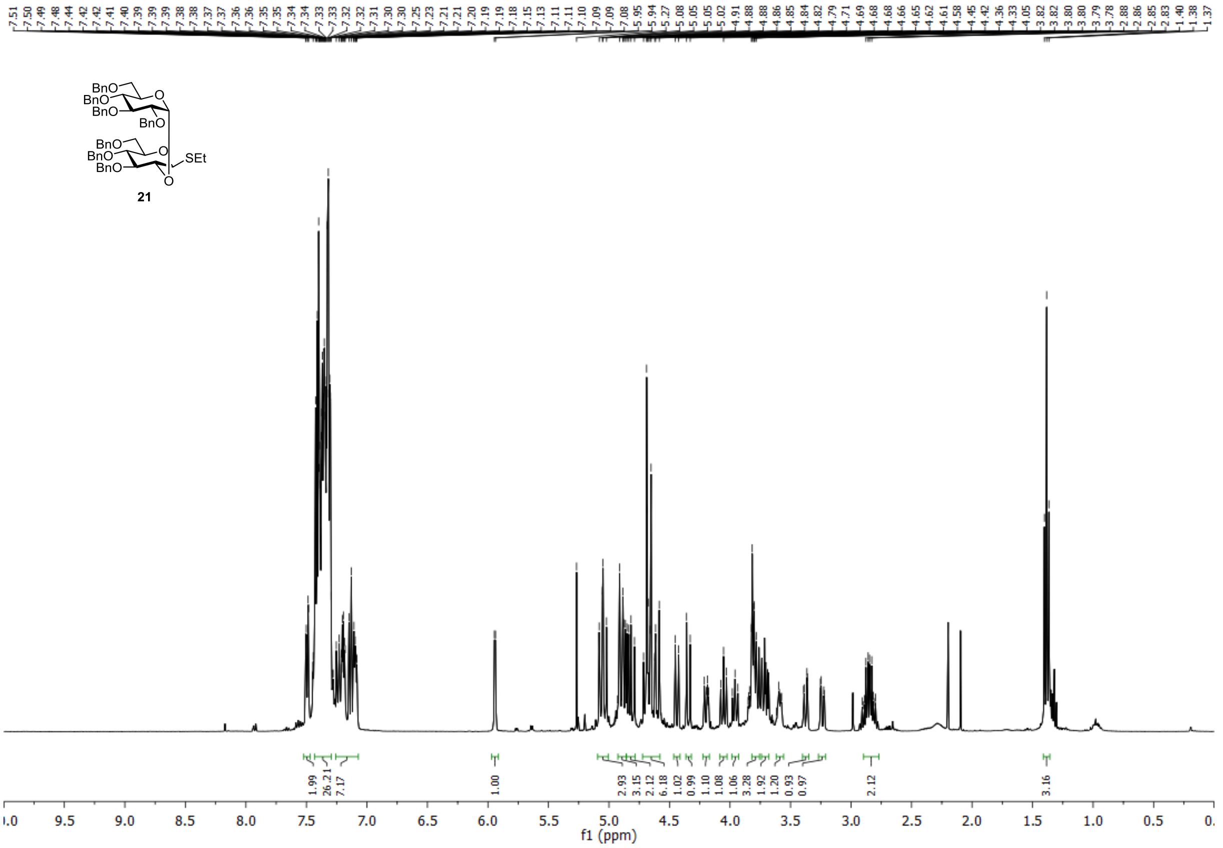

BnO
BnO
BnO
BnO
BnO
BnO
BnO
O
O
O
SEt
21
1.99
26.21
7.17
1.00
2.93
3.15
2.12
6.18
1.02
0.99
1.10
1.08
1.06
3.28
1.92
1.20
0.93
0.97
2.12
3.16
f1 (ppm)

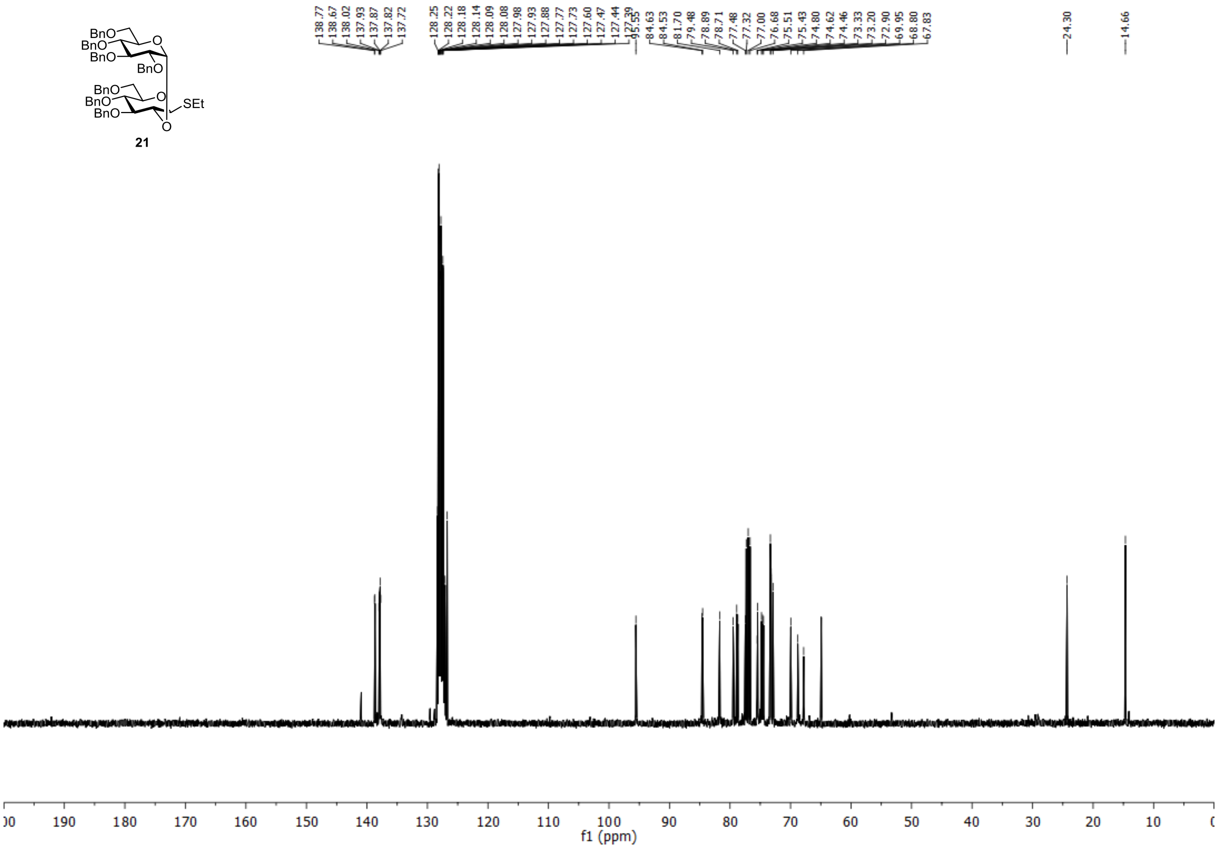
BnO
BnO
BnO
BnO
BnO
BnO
BnO
SEt
21
f1 (ppm)

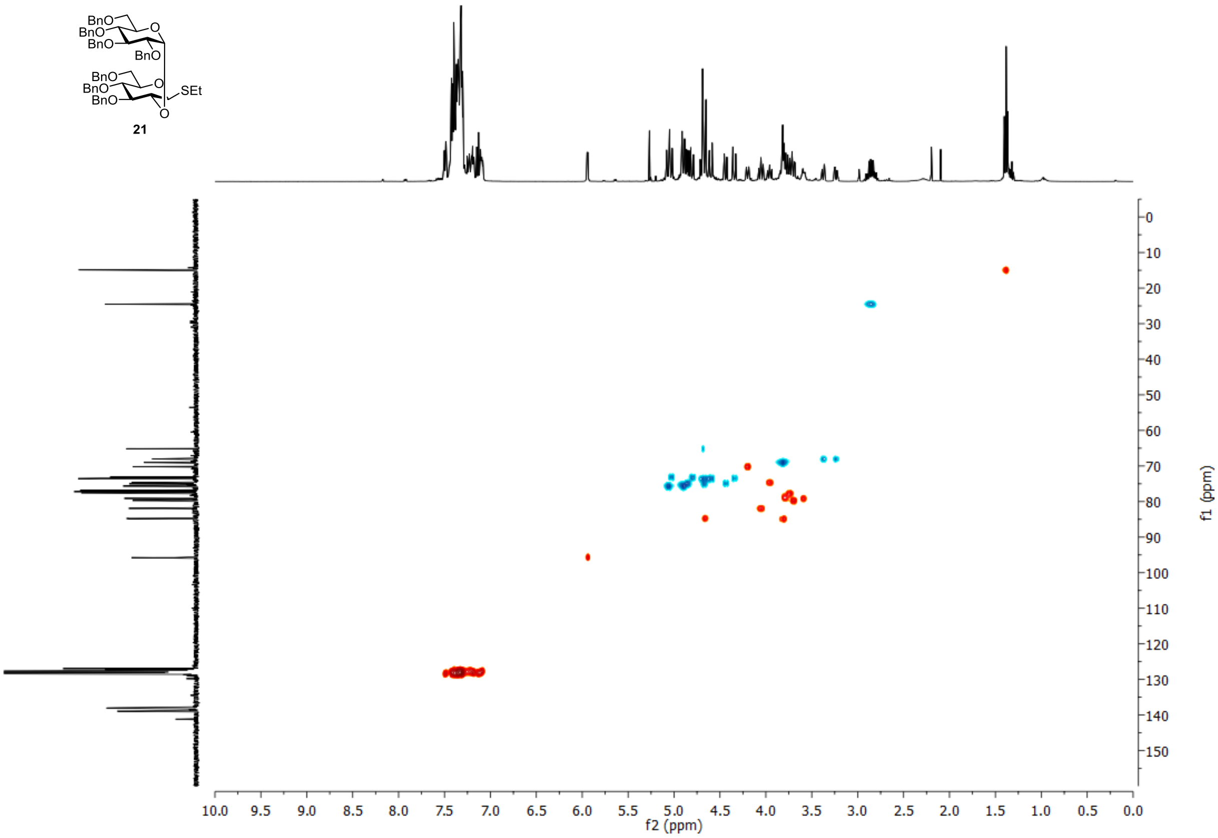
BnO
BnO
BnO
BnO
BnO
BnO
BnO
SEt
21
f2 (ppm)
f1 (ppm)

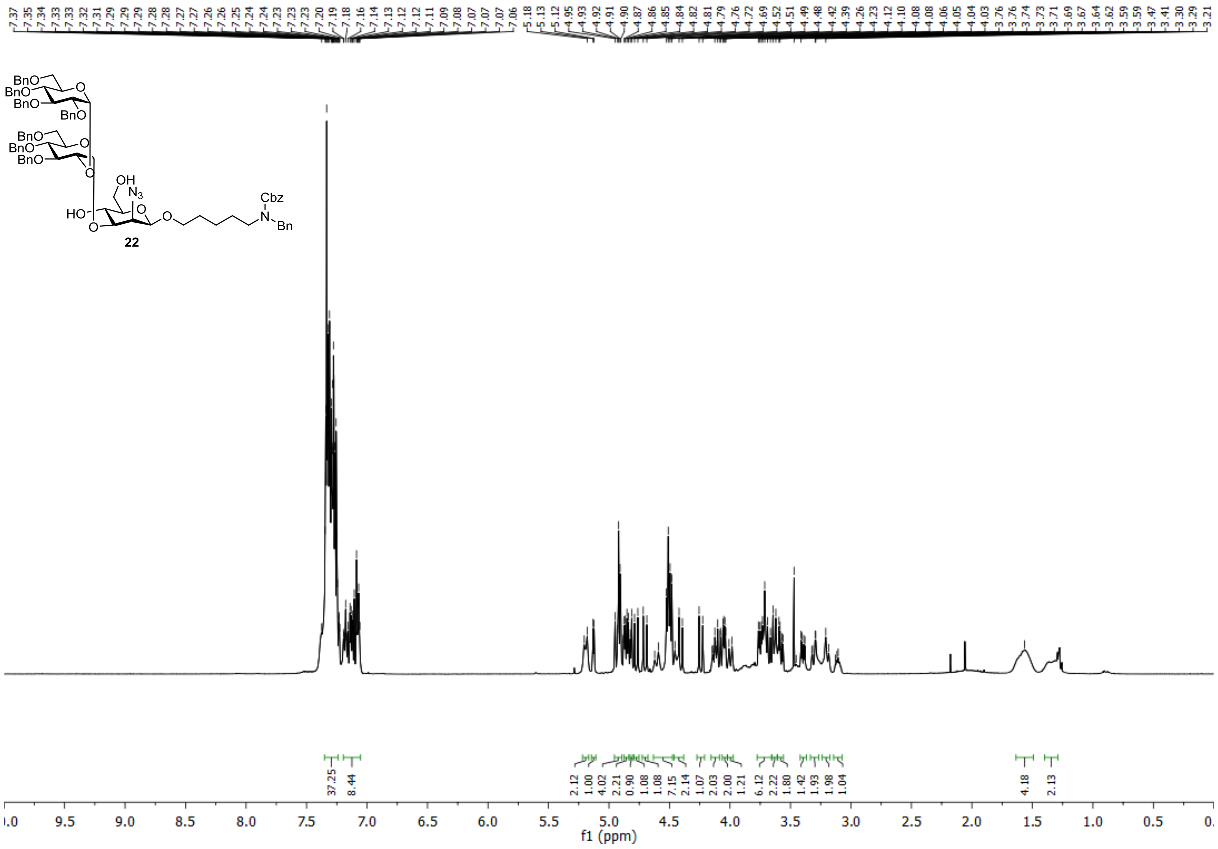
7.37 7.35 7.34 7.33 7.33 7.32 7.31 7.29 7.29 7.29 7.28 7.28 7.27 7.27 7.26 7.26 7.25 7.24 7.24 7.23 7.23 7.23 7.20 7.19 7.18 7.16 7.14 7.13 7.12 7.12 7.11 7.09 7.08 7.07 7.07 7.07 7.06 5.18 5.13 5.12 4.95 4.93 4.92 4.91 4.90 4.87 4.86 4.85 4.84 4.82 4.81 4.79 4.76 4.72 4.69 4.52 4.51 4.49 4.48 4.42 4.39 4.26 4.23 4.12 4.10 4.08 4.08 4.06 4.05 4.04 4.03 3.76 3.76 3.74 3.73 3.71 3.69 3.67 3.64 3.62 3.59 3.59 3.47 3.41 3.30 3.29 3.21
BnO
BnO
BnO
BnO
O
BnO
BnO
BnO
O
OH
N_3
HO
O
O
O
Cbz
N
Bn
22
37.25 8.44 2.12 1.00 4.02 2.21 0.90 1.08 1.08 7.15 2.14 1.07 2.03 2.00 1.21 6.12 2.22 1.80 1.42 1.93 1.98 1.04 4.18 2.13
10.0 9.5 9.0 8.5 8.0 7.5 7.0 6.5 6.0 5.5 5.0 4.5 4.0 3.5 3.0 2.5 2.0 1.5 1.0 0.5 0.0
f1 (ppm)

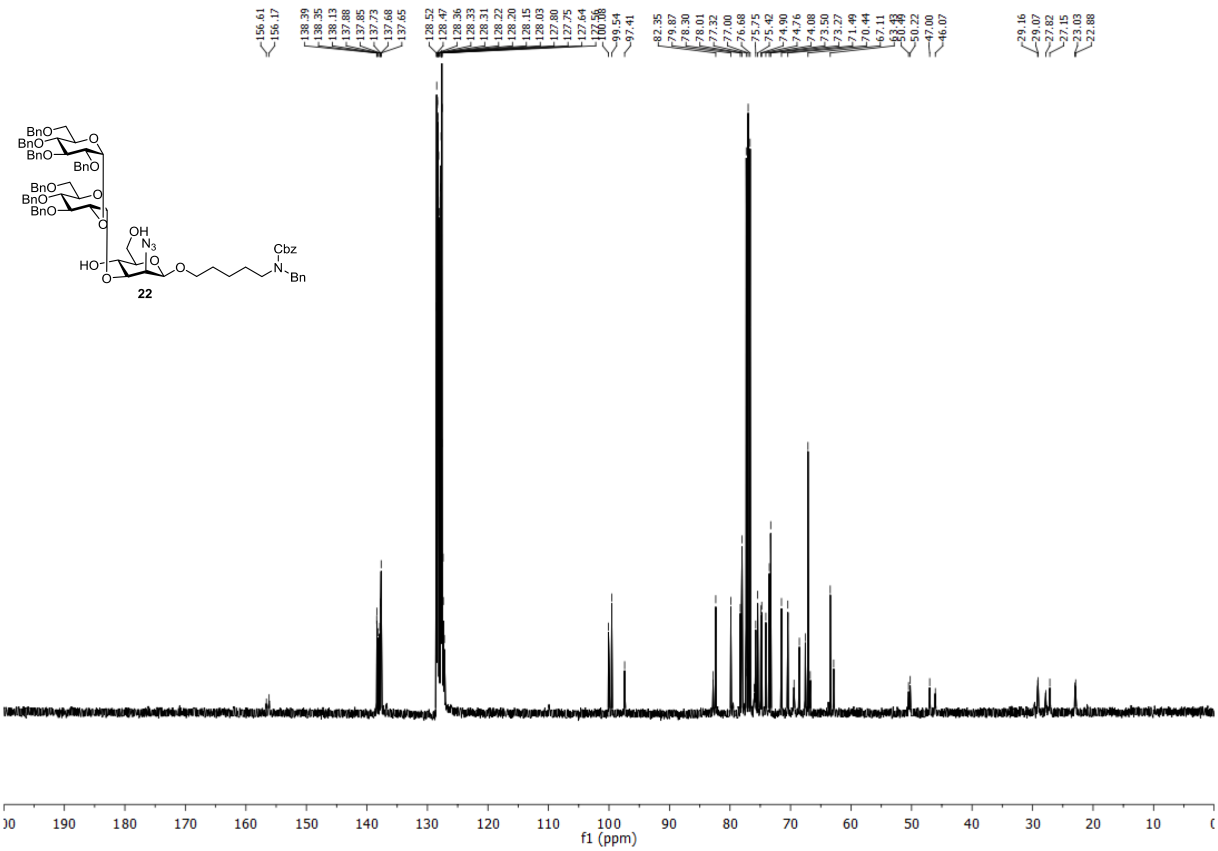
156.61 156.17 138.39 138.35 138.13 137.88 137.85 137.73 137.68 137.65 128.52 128.47 128.36 128.33 128.31 128.22 128.20 128.15 128.03 127.80 127.75 127.64 127.56 100.08 99.54 97.41 82.35 79.87 78.30 78.01 77.32 77.00 76.68 75.75 75.42 74.90 74.76 74.08 73.50 73.27 71.49 70.44 67.11 63.43 50.48 50.22 47.00 46.07 29.16 29.07 27.82 27.15 23.03 22.88
BnO
BnO
BnO
BnO
O
BnO
BnO
BnO
O
OH
N_3
HO
O
O
O
Cbz
N
Bn
22
200 190 180 170 160 150 140 130 120 110 100 90 80 70 60 50 40 30 20 10 0
f1 (ppm)

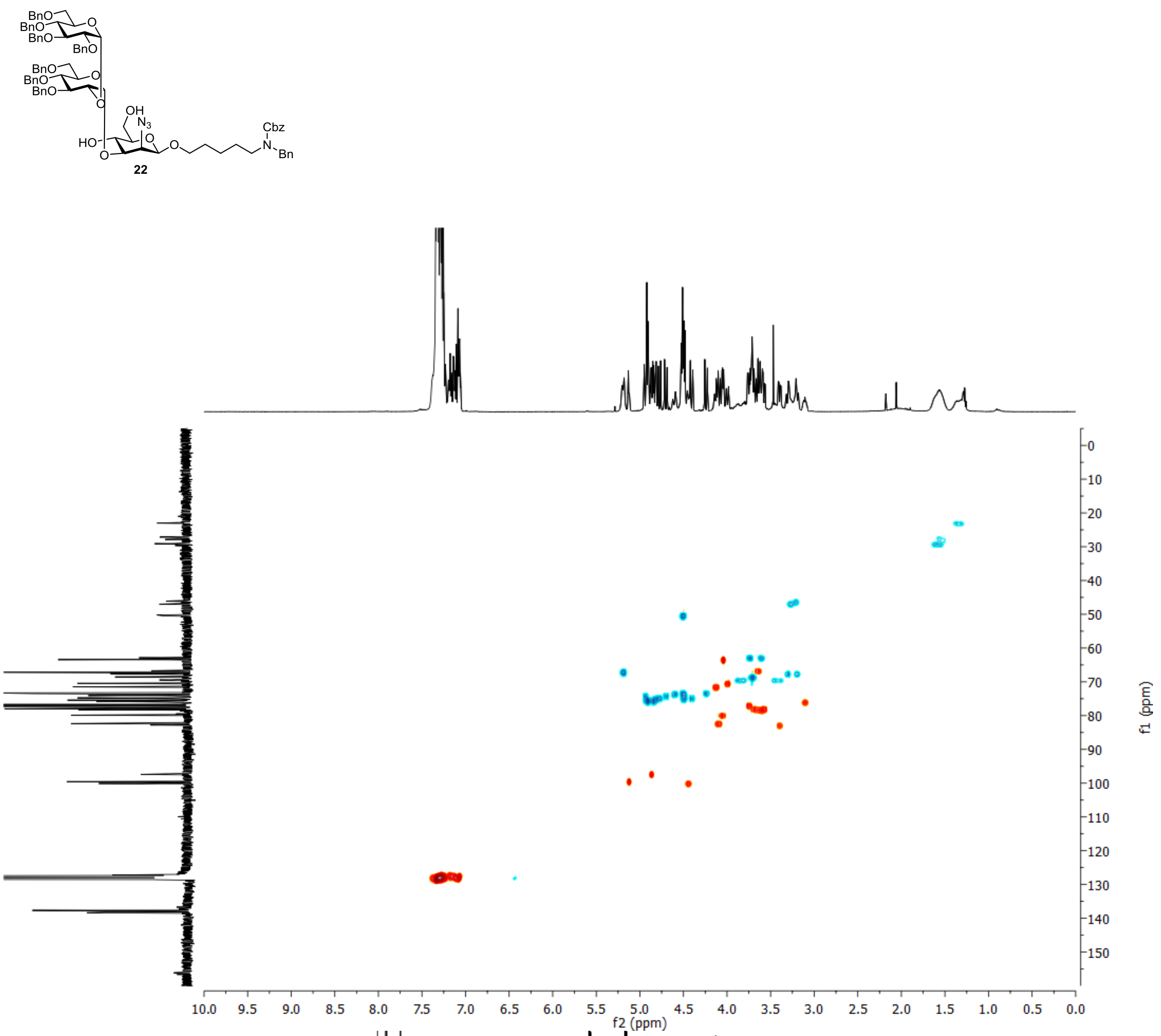
22
f2 (ppm)
f1 (ppm)

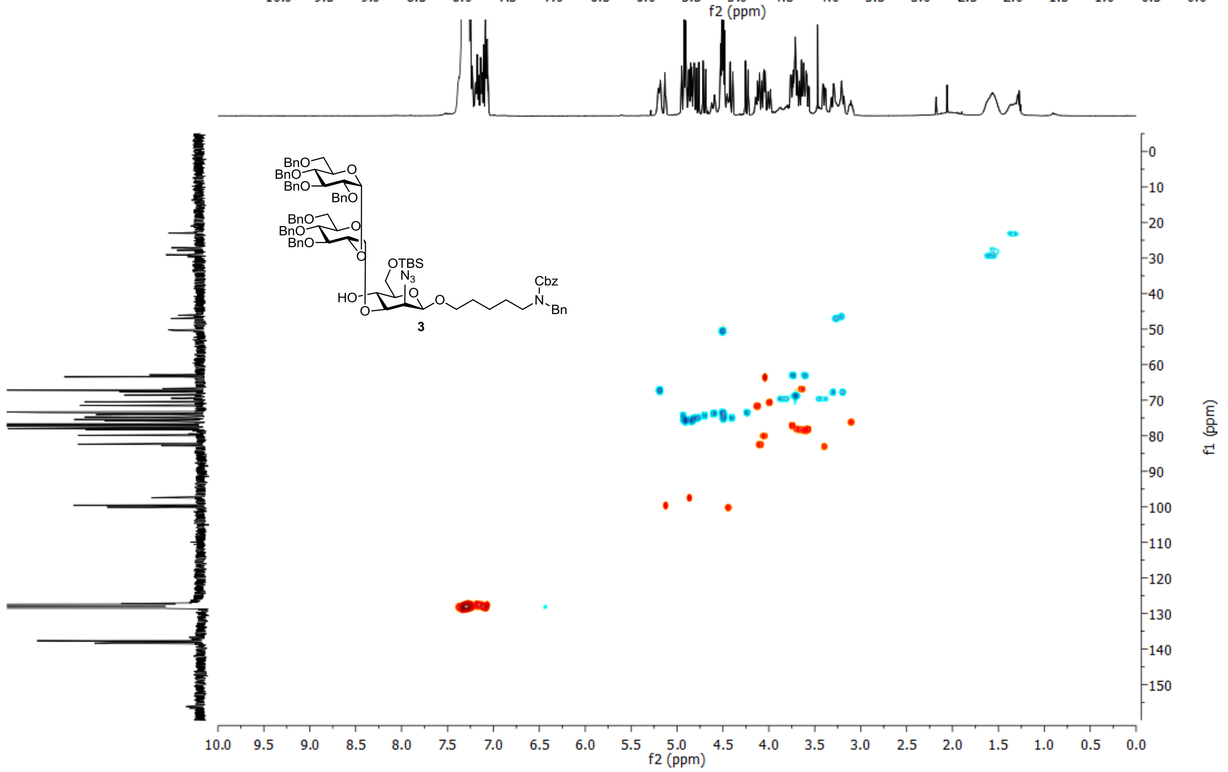
3
f2 (ppm)
f1 (ppm)

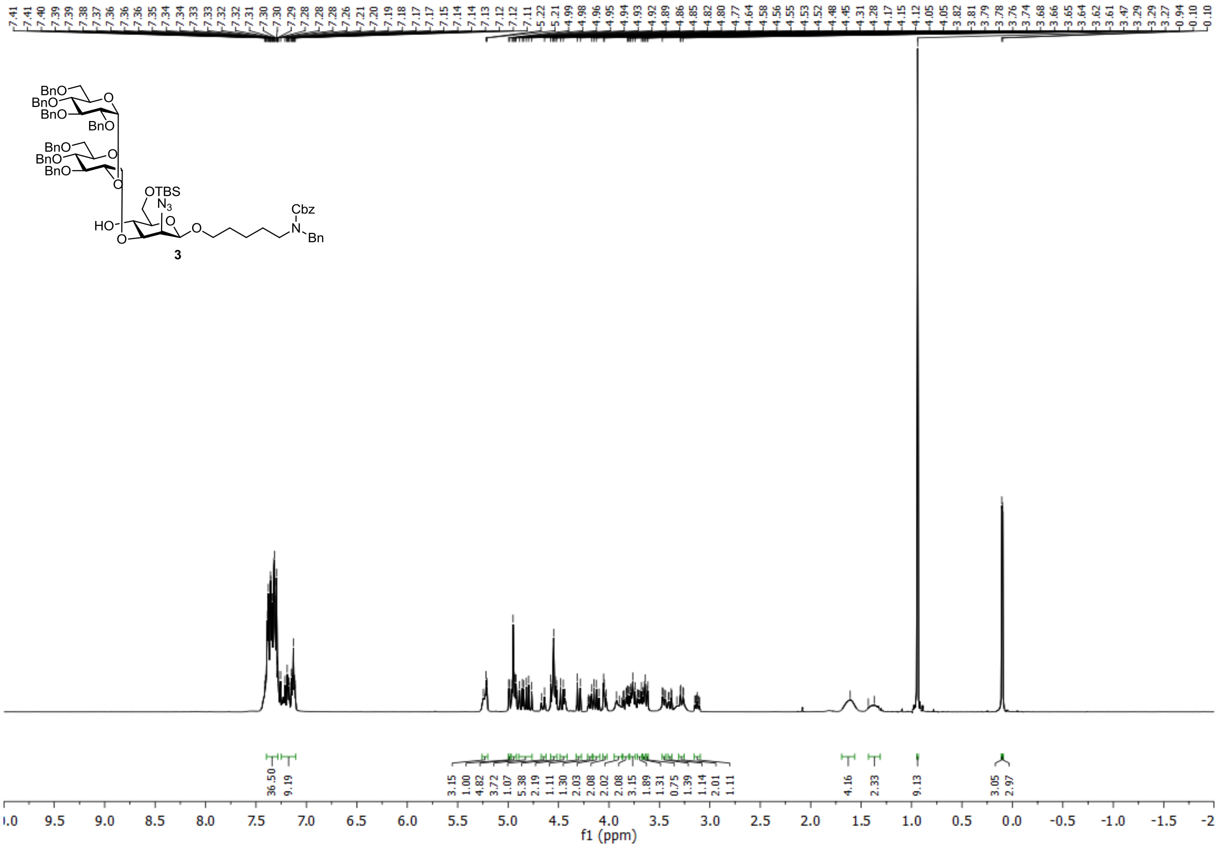
7.41 7.41 7.40 7.39 7.39 7.38 7.37 7.36 7.36 7.36 7.35 7.34 7.34 7.33 7.33 7.32 7.31 7.30 7.30 7.29 7.28 7.28 7.26 7.21 7.20 7.19 7.18 7.17 7.17 7.15 7.14 7.14 7.13 7.12 7.12 7.11 5.22 5.21 4.99 4.98 4.96 4.95 4.94 4.93 4.92 4.89 4.86 4.85 4.82 4.80 4.77 4.64 4.58 4.56 4.55 4.53 4.52 4.48 4.45 4.31 4.28 4.17 4.15 4.12 4.05 4.05 3.82 3.81 3.79 3.78 3.76 3.74 3.68 3.66 3.65 3.64 3.62 3.61 3.47 3.29 3.27 0.94 0.10 0.10
BnO
BnO
BnO
BnO
BnO
BnO
BnO
BnO
OTBS
N_3
Cbz
HO
Bn
3
36.50 9.19 3.15 1.00 4.82 3.72 1.07 5.38 2.19 1.11 1.30 2.03 2.08 2.02 2.08 3.15 1.89 1.31 0.75 1.39 1.14 2.01 1.11 4.16 2.33 9.13 3.05 2.97
f1 (ppm)

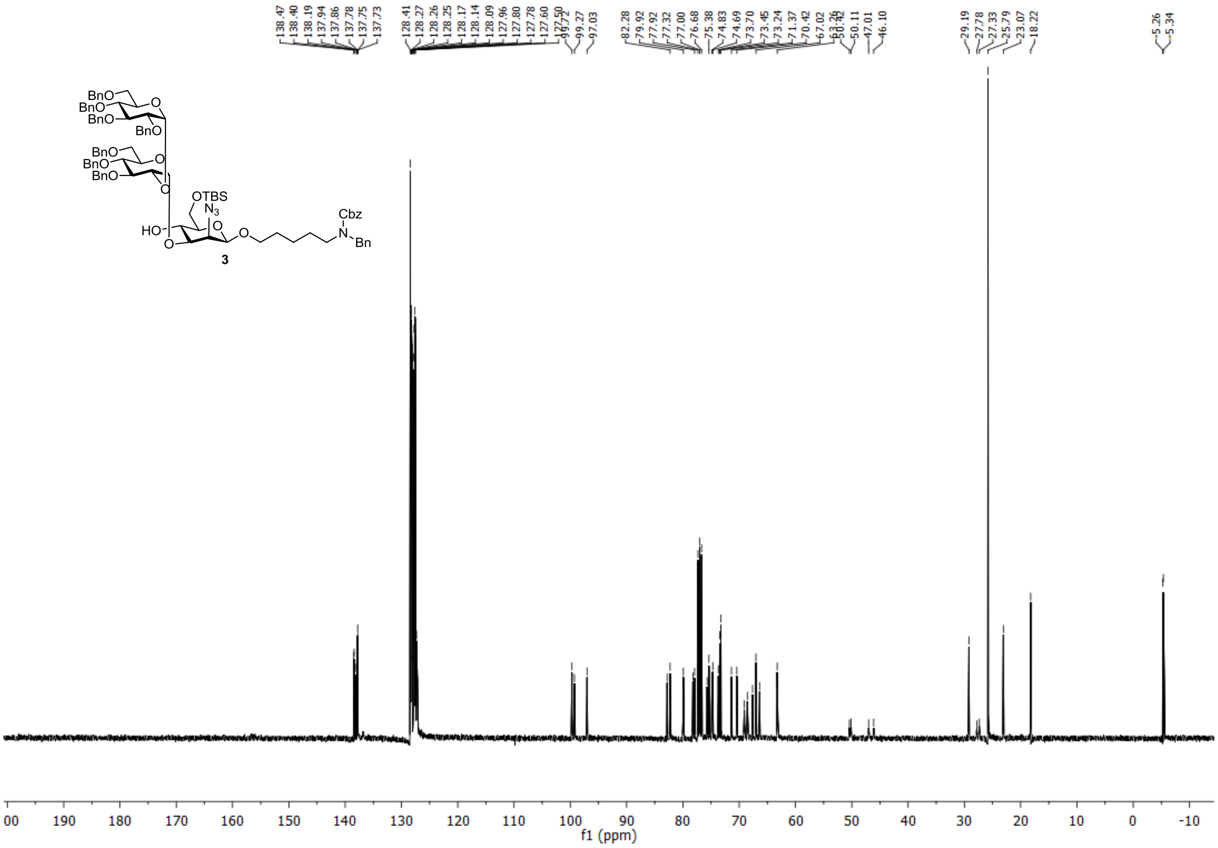
138.47 138.40 138.19 137.94 137.86 137.78 137.75 137.73 128.41 128.27 128.26 128.25 128.17 128.14 128.09 127.96 127.80 127.78 127.60 99.27 97.03 82.28 79.92 77.92 77.32 77.00 76.68 75.38 74.83 74.69 73.70 73.45 73.24 71.37 70.42 67.02 50.11 47.01 46.10 29.19 27.78 27.33 25.79 23.07 18.22 -5.26 -5.34
BnO
BnO
BnO
BnO
BnO
BnO
BnO
BnO
OTBS
N_3
Cbz
HO
Bn
3
f1 (ppm)

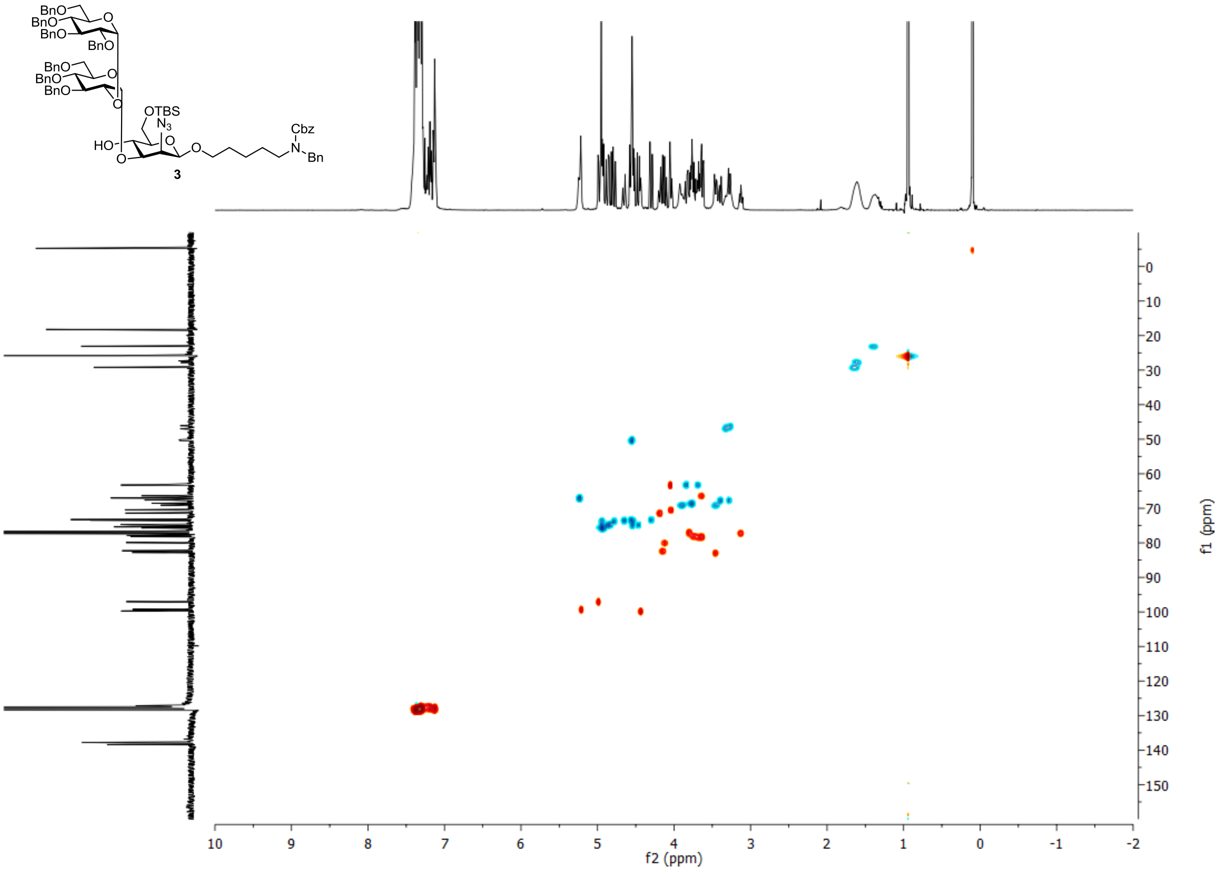

BnO
BnO
BnO
BnO
O
BnO
BnO
BnO
BnO
O
OTBS
N3
HO
O
Cbz
N
Bn
3
f2 (ppm)
f1 (ppm)

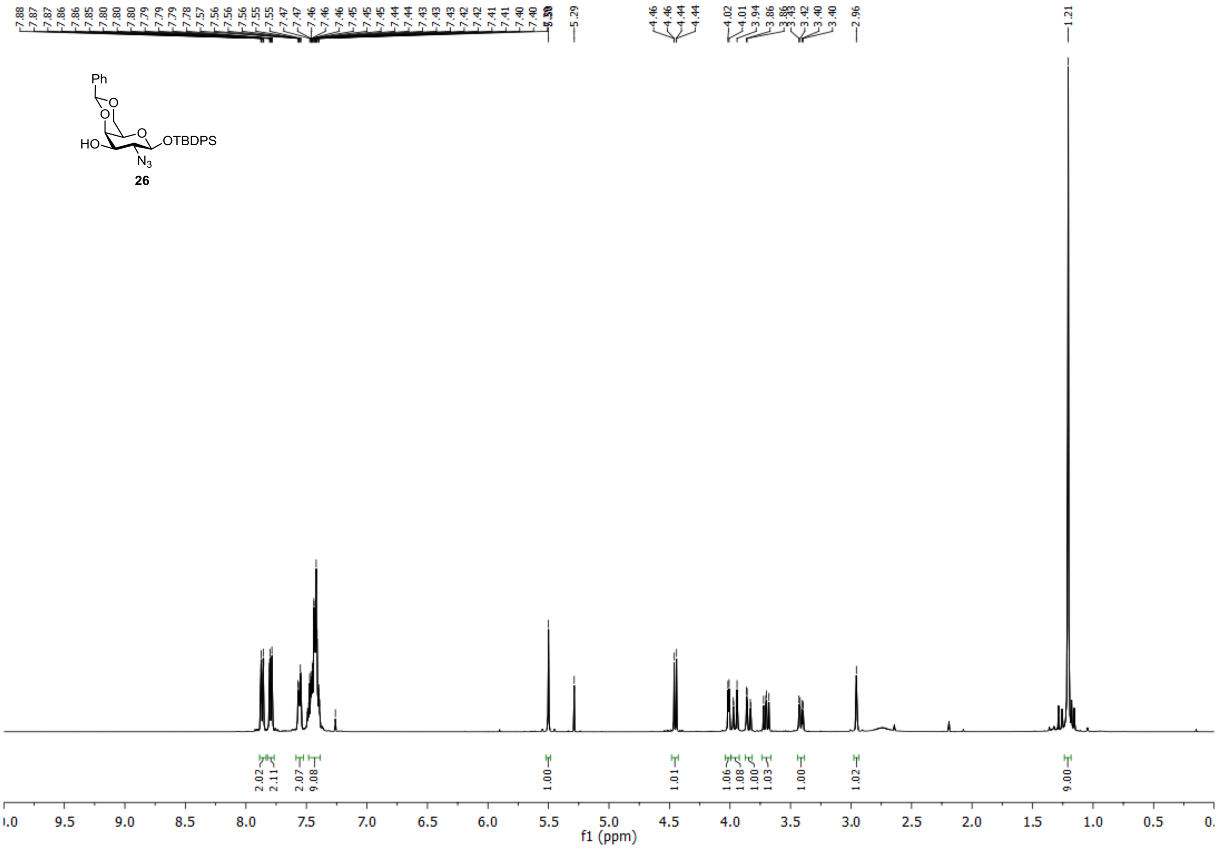

Ph
O
O
HO
O
OTBDPS
N3
26
f1 (ppm)

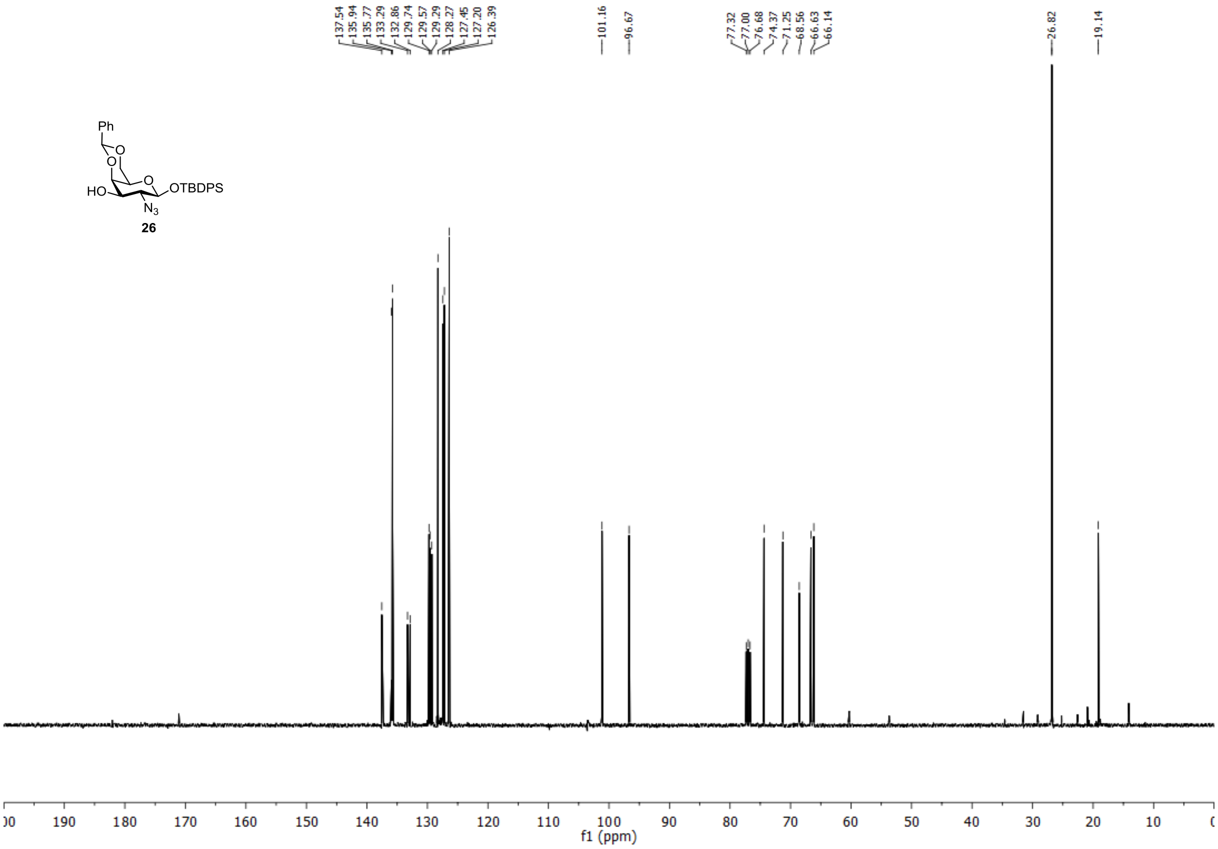

137.54
135.94
135.77
133.29
132.86
129.74
129.57
129.29
128.27
127.45
127.20
126.39
101.16
96.67
77.32
77.00
76.68
74.37
71.25
68.56
66.63
66.14
26.82
19.14
Ph
O
O
HO
O
OTBDPS
N3
26
f1 (ppm)

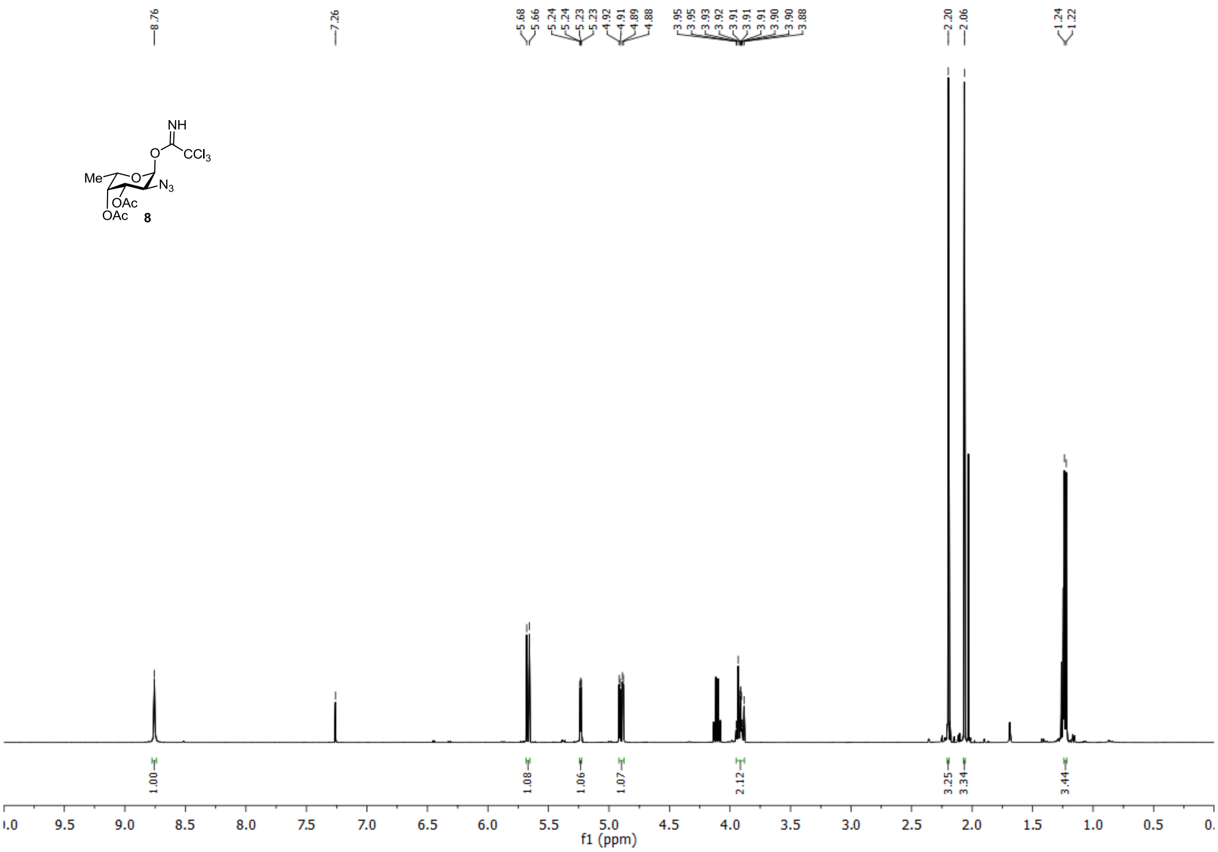

8.76
7.26
5.68
5.66
5.24
5.24
5.23
5.23
4.92
4.91
4.89
4.88
3.95
3.95
3.93
3.92
3.91
3.91
3.91
3.90
3.90
3.88
2.20
2.06
1.24
1.22
NH
O
CCl3
Me
O
N3
OAc
OAc
8
1.00
1.08
1.06
1.07
2.12
3.25
3.34
3.44
f1 (ppm)

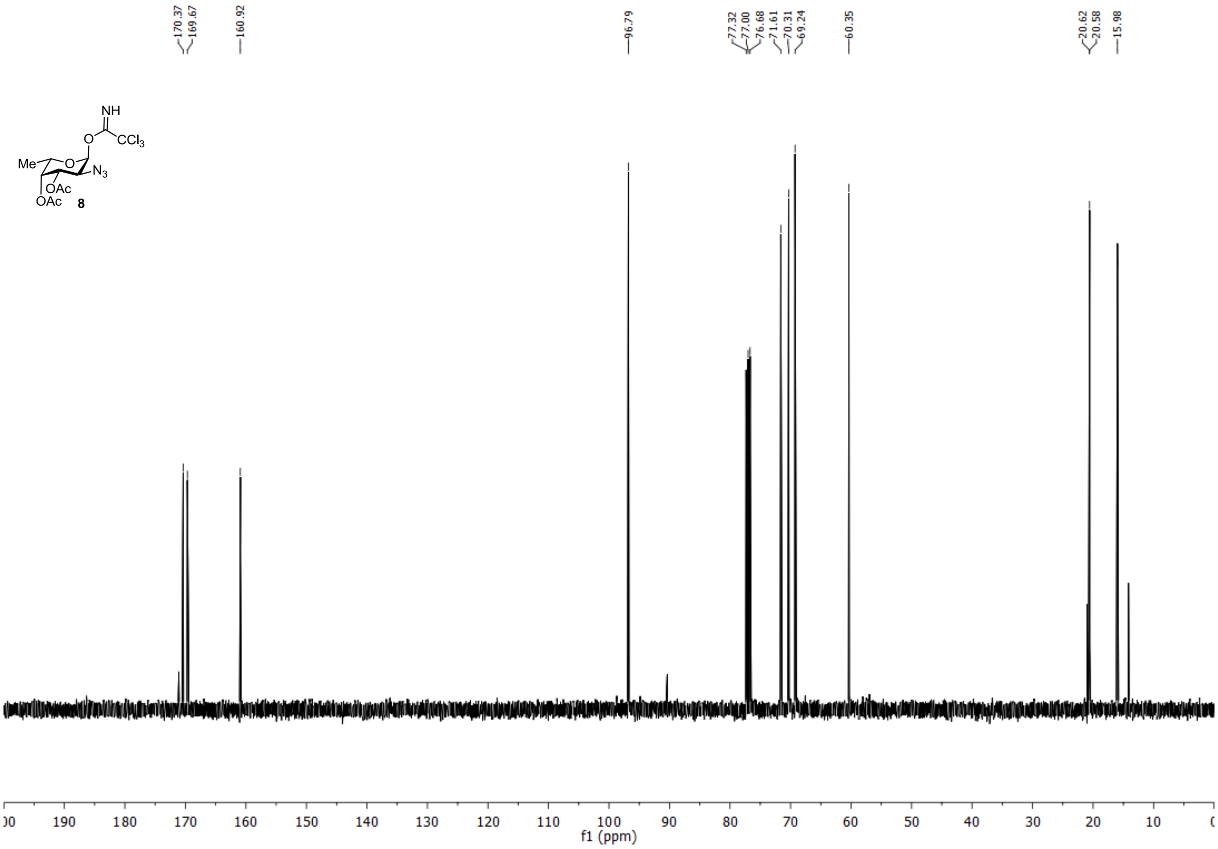
170.37
169.67
160.92
96.79
77.32
77.00
76.68
71.61
70.31
69.24
60.35
20.62
20.58
15.98
NH
O
CCl3
Me
O
N3
OAc
OAc
8
f1 (ppm)

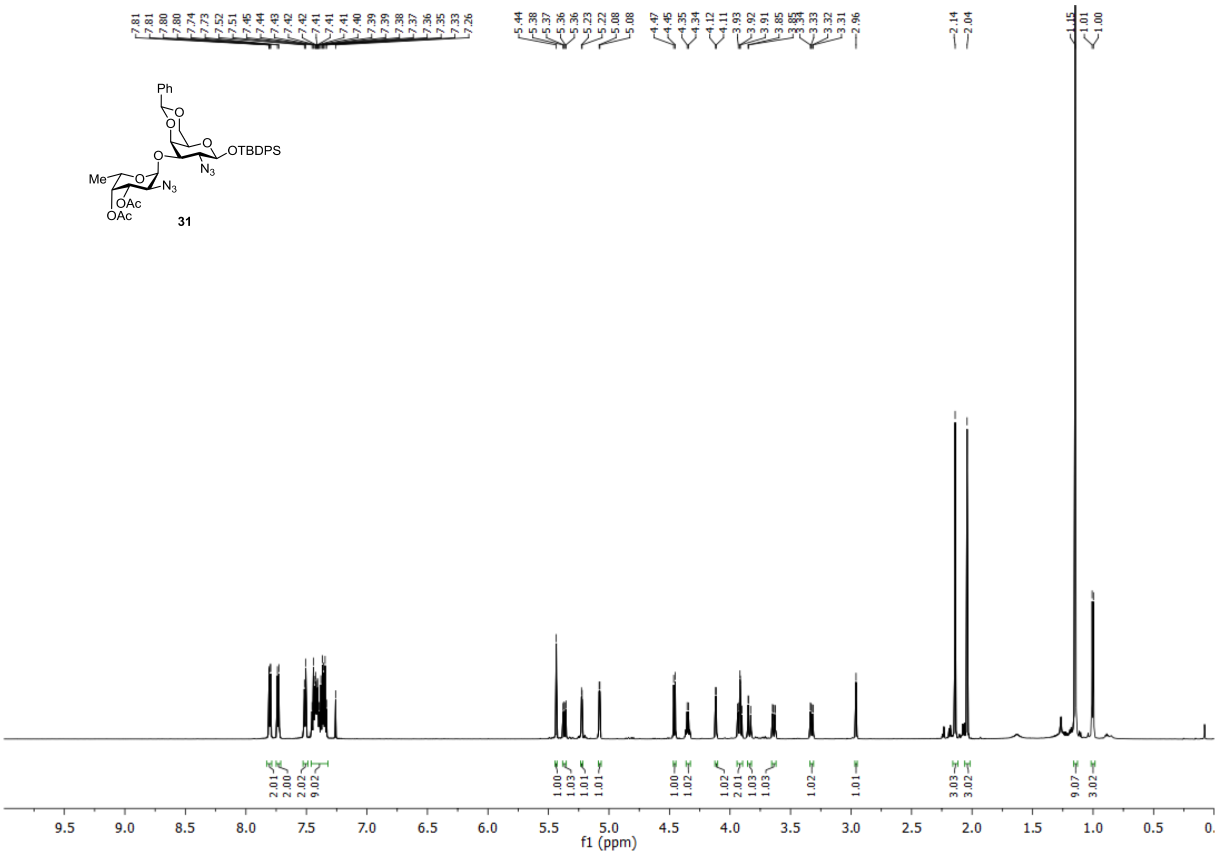
7.81
7.81
7.80
7.80
7.74
7.73
7.52
7.51
7.45
7.44
7.43
7.42
7.41
7.41
7.41
7.40
7.39
7.39
7.38
7.37
7.36
7.35
7.33
7.26
5.44
5.38
5.37
5.36
5.36
5.23
5.22
5.08
5.08
4.47
4.45
4.35
4.34
4.12
4.11
3.93
3.92
3.91
3.85
3.33
3.32
3.31
2.96
2.14
2.04
1.15
1.01
1.00
Ph
O
O
O
O
OTBDPS
N3
Me
O
N3
OAc
OAc
31
2.01
2.00
2.02
9.02
1.00
1.03
1.01
1.01
1.00
1.02
1.02
2.01
1.03
1.03
1.02
1.01
3.03
3.02
9.07
3.02
f1 (ppm)

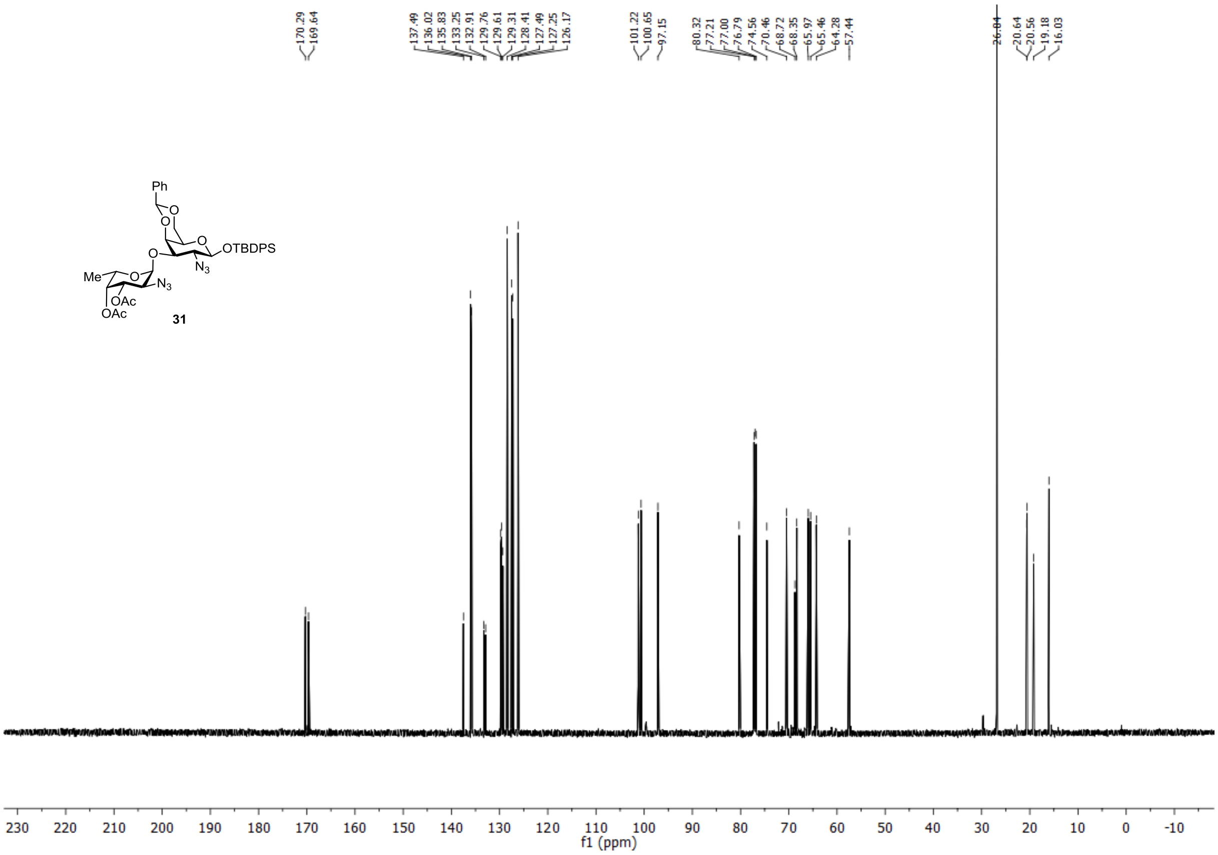
170.29
169.64
137.49
136.02
135.83
133.25
132.91
129.76
129.61
129.31
128.41
127.49
127.25
126.17
101.22
100.65
97.15
80.32
77.21
77.00
76.79
74.56
70.46
68.72
68.35
65.97
65.46
64.28
57.44
26.84
20.64
20.56
19.18
16.03
Ph
O
O
O
O
OTBDPS
N3
Me
O
N3
OAc
OAc
31
230 220 210 200 190 180 170 160 150 140 130 120 110 100 90 80 70 60 50 40 30 20 10 0 -10
f1 (ppm)

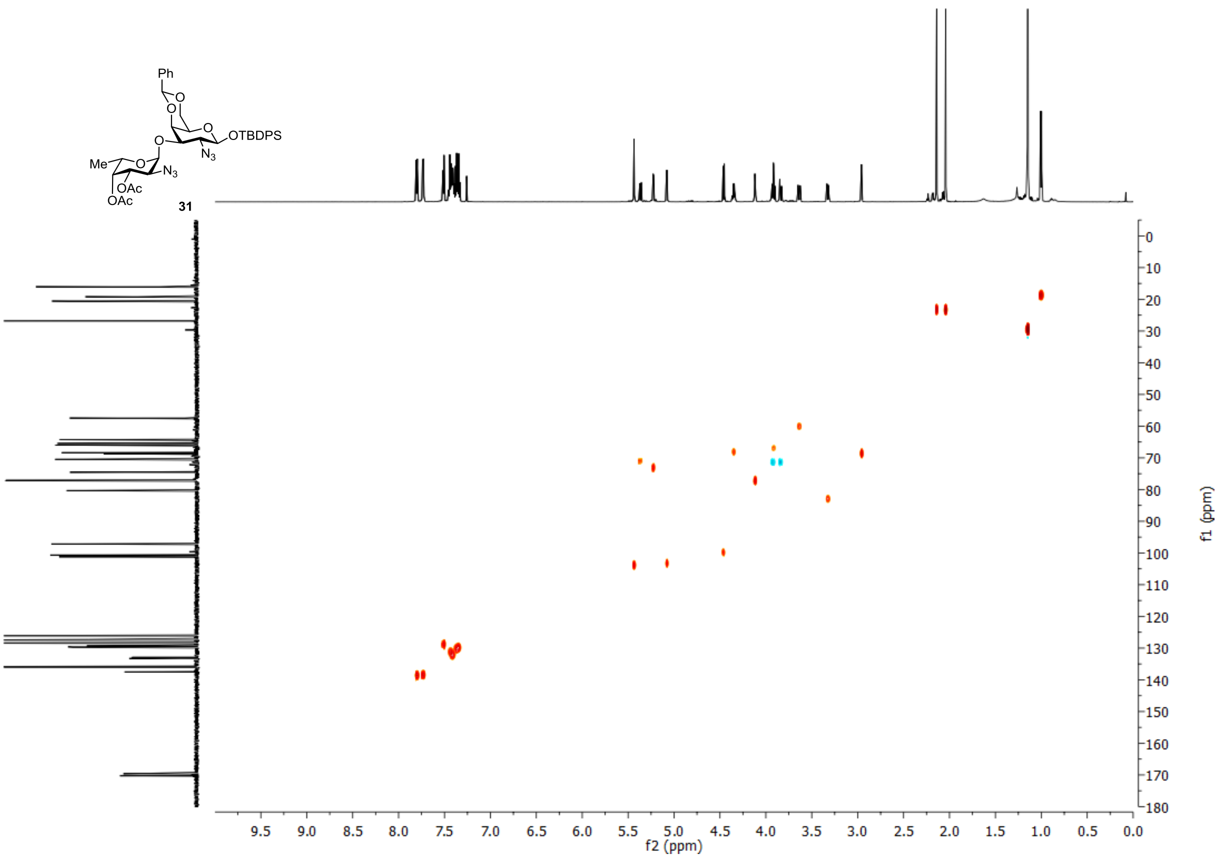

OTBDPS
31
f2 (ppm)
f1 (ppm)

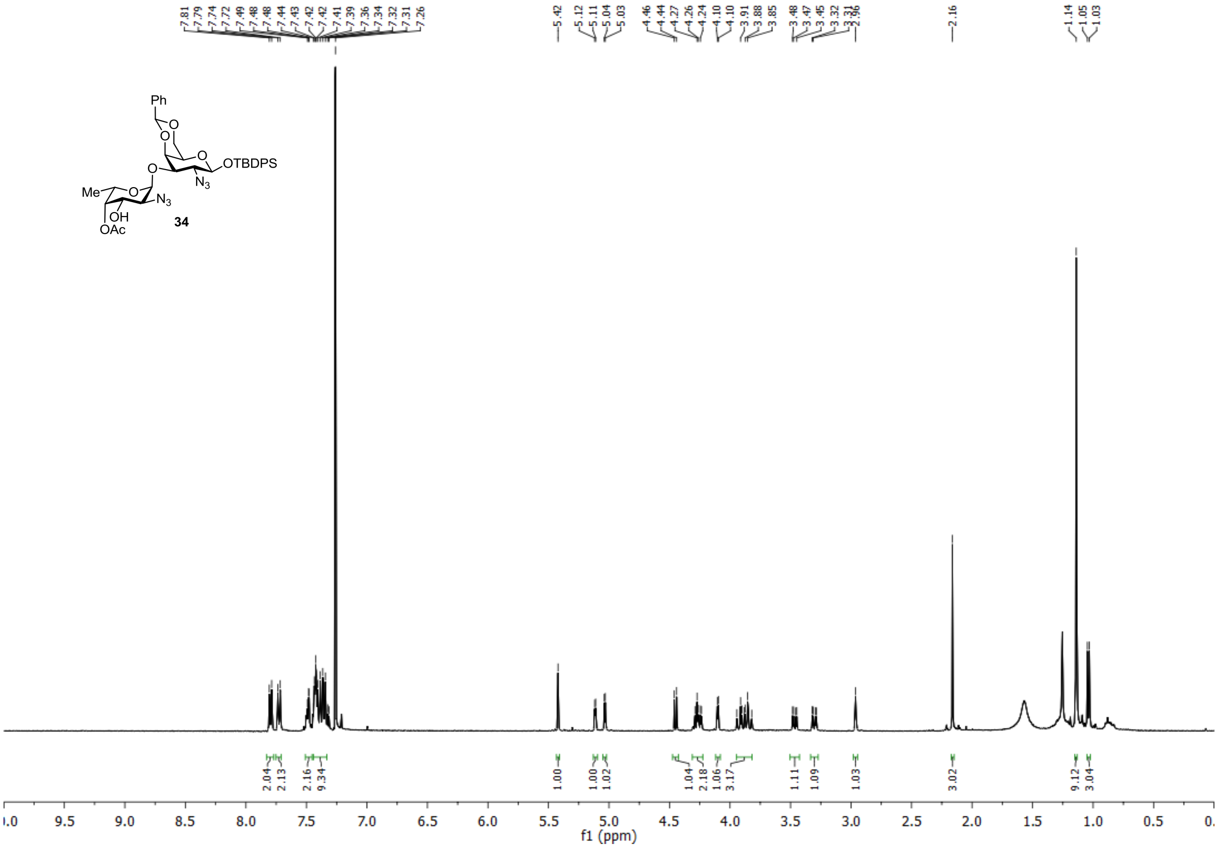

OTBDPS
34
f1 (ppm)

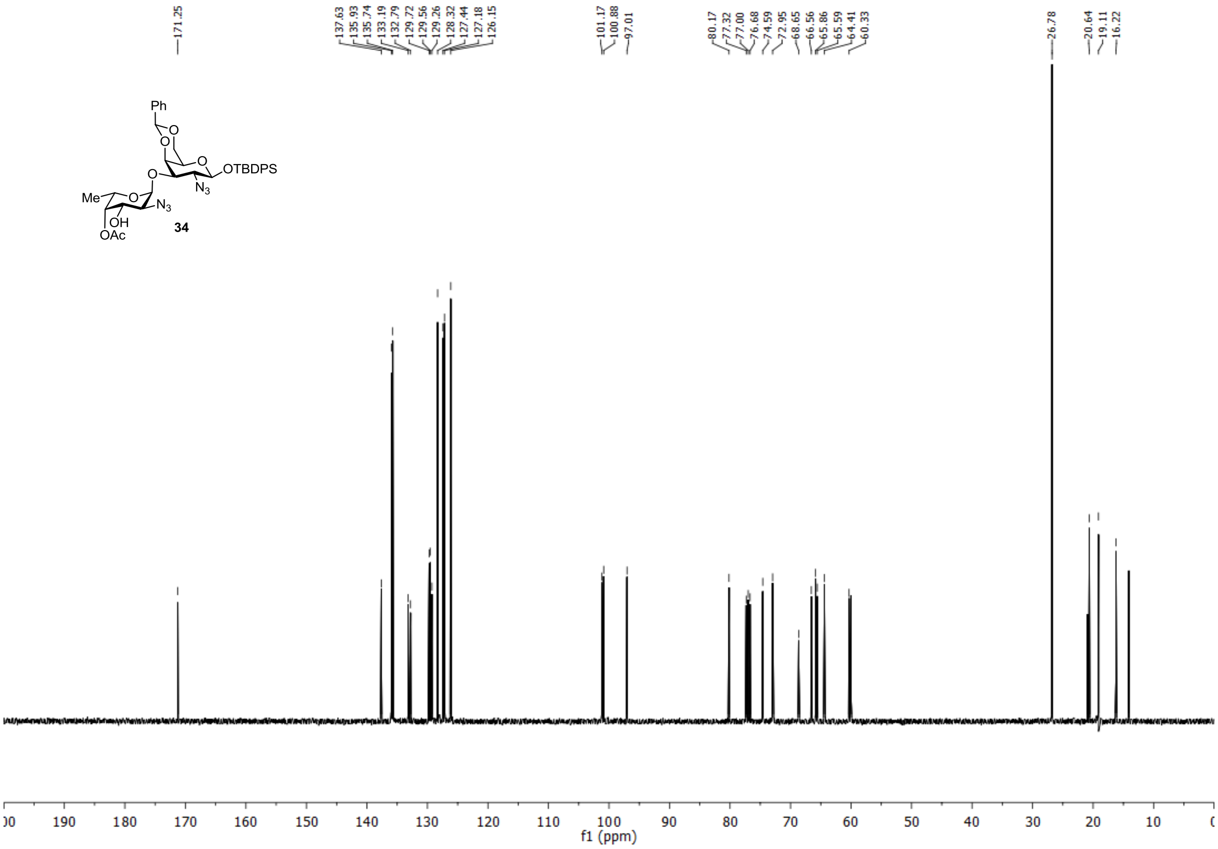

171.25
137.63
135.93
135.74
133.19
132.79
129.72
129.56
129.26
128.32
127.44
127.18
126.15
101.17
100.88
97.01
80.17
77.32
77.00
76.68
74.59
72.95
68.65
66.56
65.86
65.59
64.41
60.33
26.78
20.64
19.11
16.22
Ph
O
O
O
OTBDPS
N3
O
Me
O
N3
OH
OAc
34
f1 (ppm)

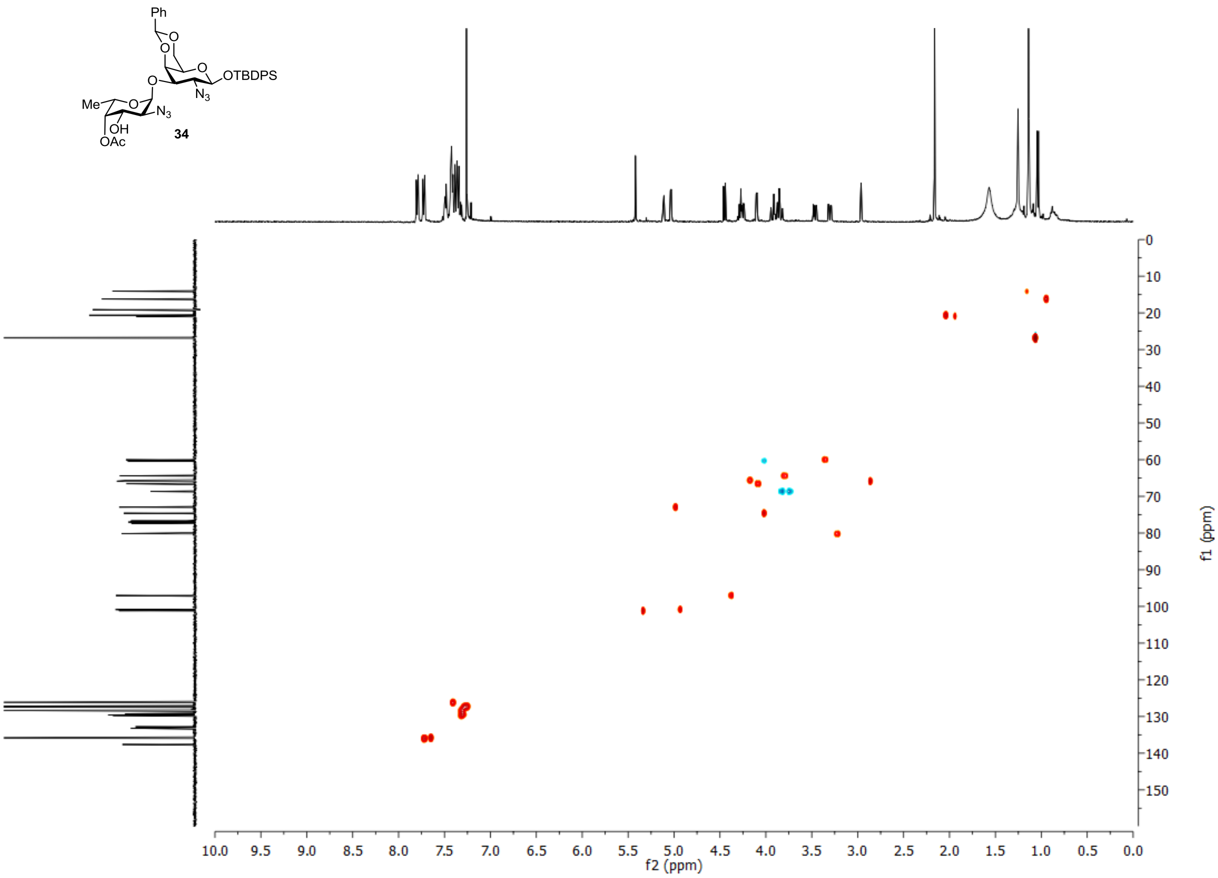

Ph
O
O
O
OTBDPS
N3
O
Me
O
N3
OH
OAc
34
f2 (ppm)
f1 (ppm)

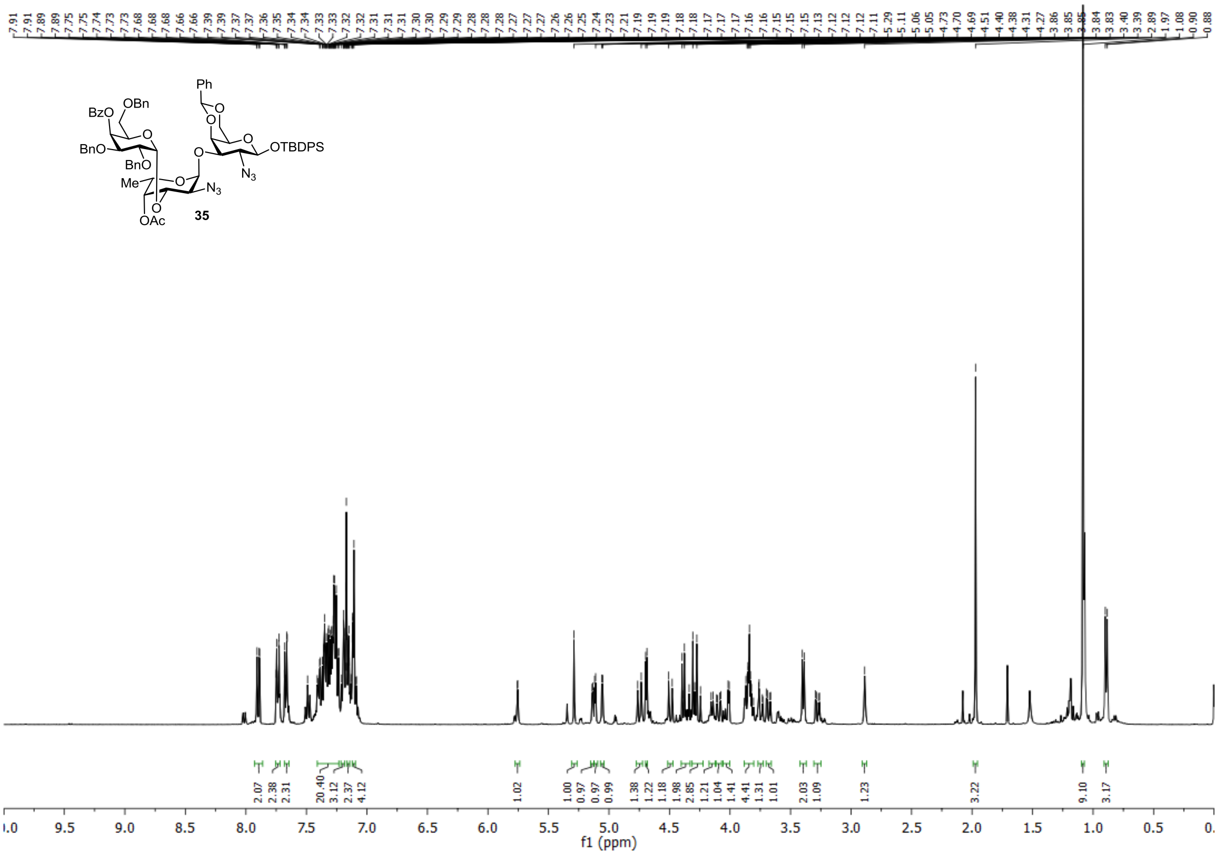

BzO
OBn
BnO
BnO
Me
O
OAc
N3
Ph
O
O
OTBDPS
N3
35
f1 (ppm)

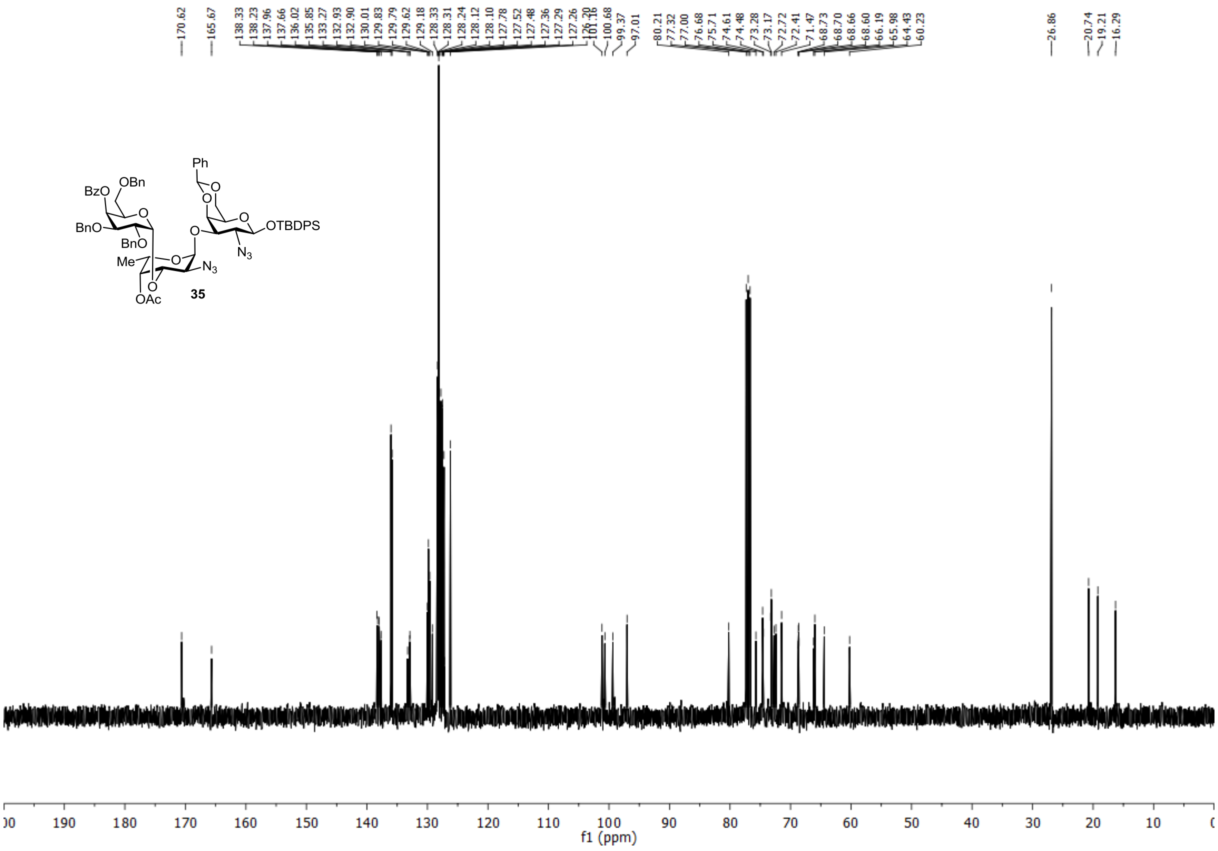

BzO
OBn
BnO
BnO
Me
O
OAc
N3
Ph
O
O
OTBDPS
N3
35
f1 (ppm)

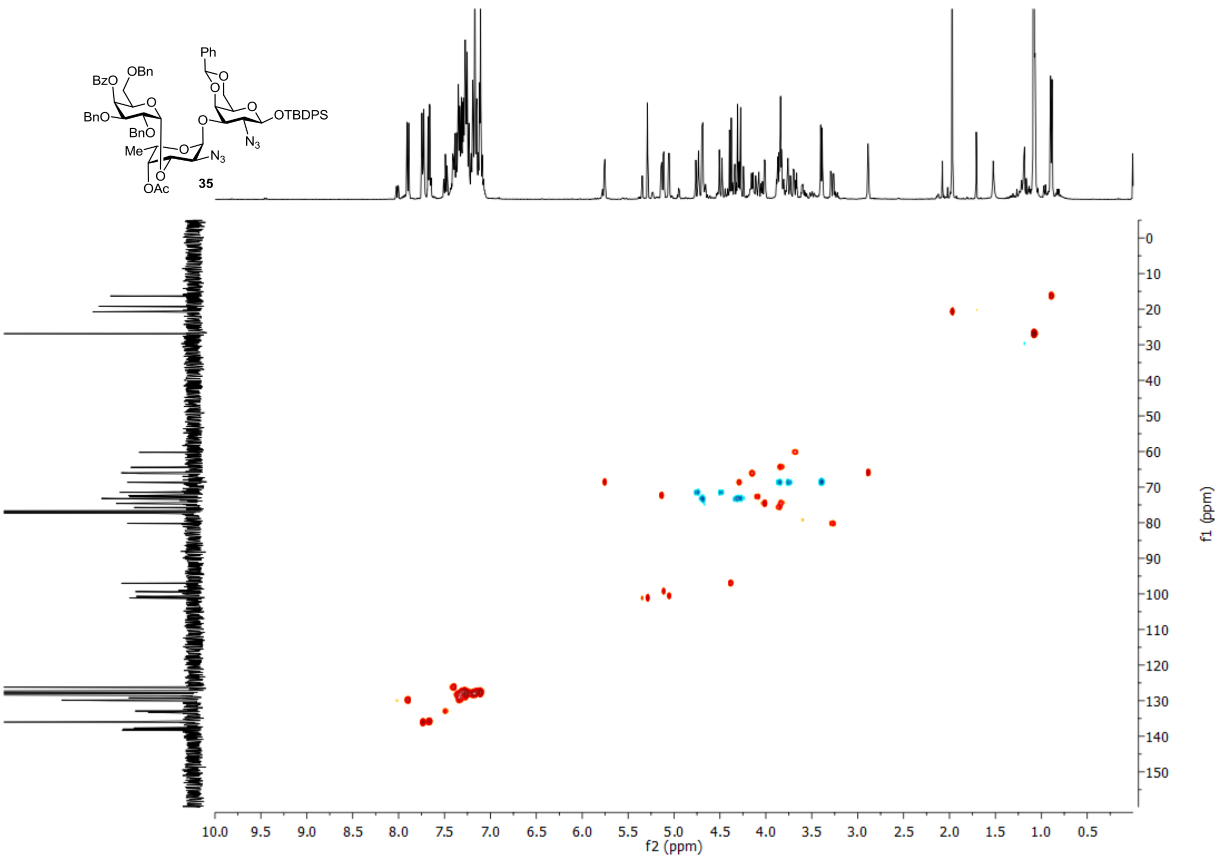
BzO
OBn
BnO
BnO
Me
OAc
Ph
O
OTBDPS
N3
35
f2 (ppm)
f1 (ppm)

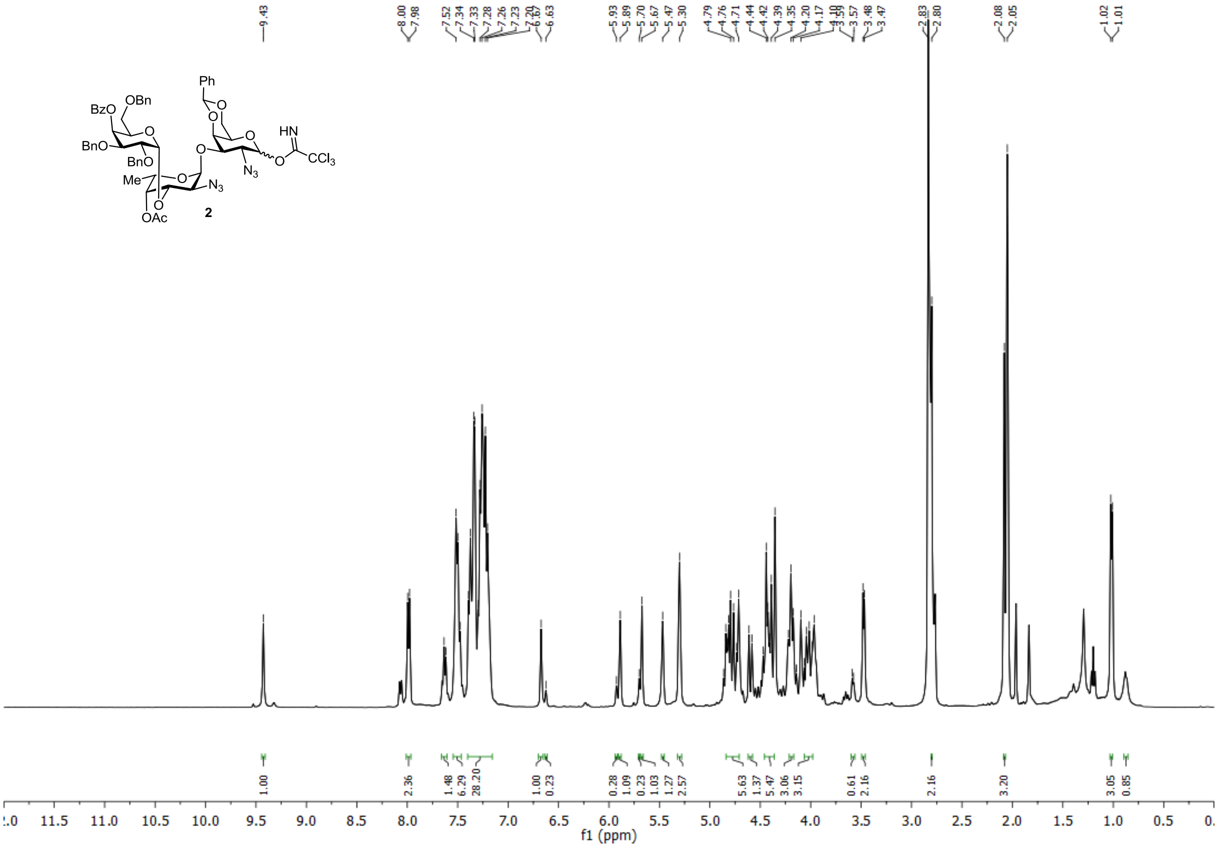
BzO
OBn
BnO
BnO
Me
OAc
Ph
HN
CCl3
N3
2
f1 (ppm)

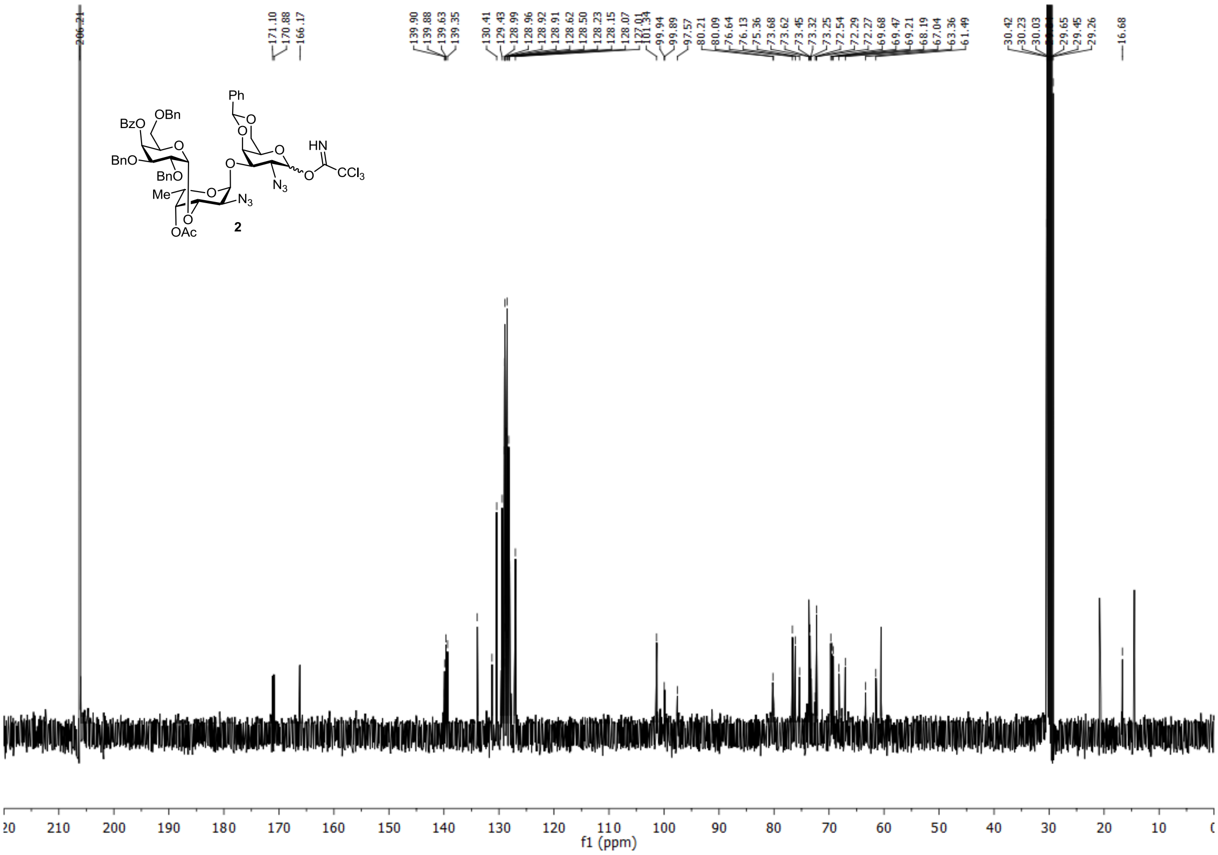
2
f1 (ppm)

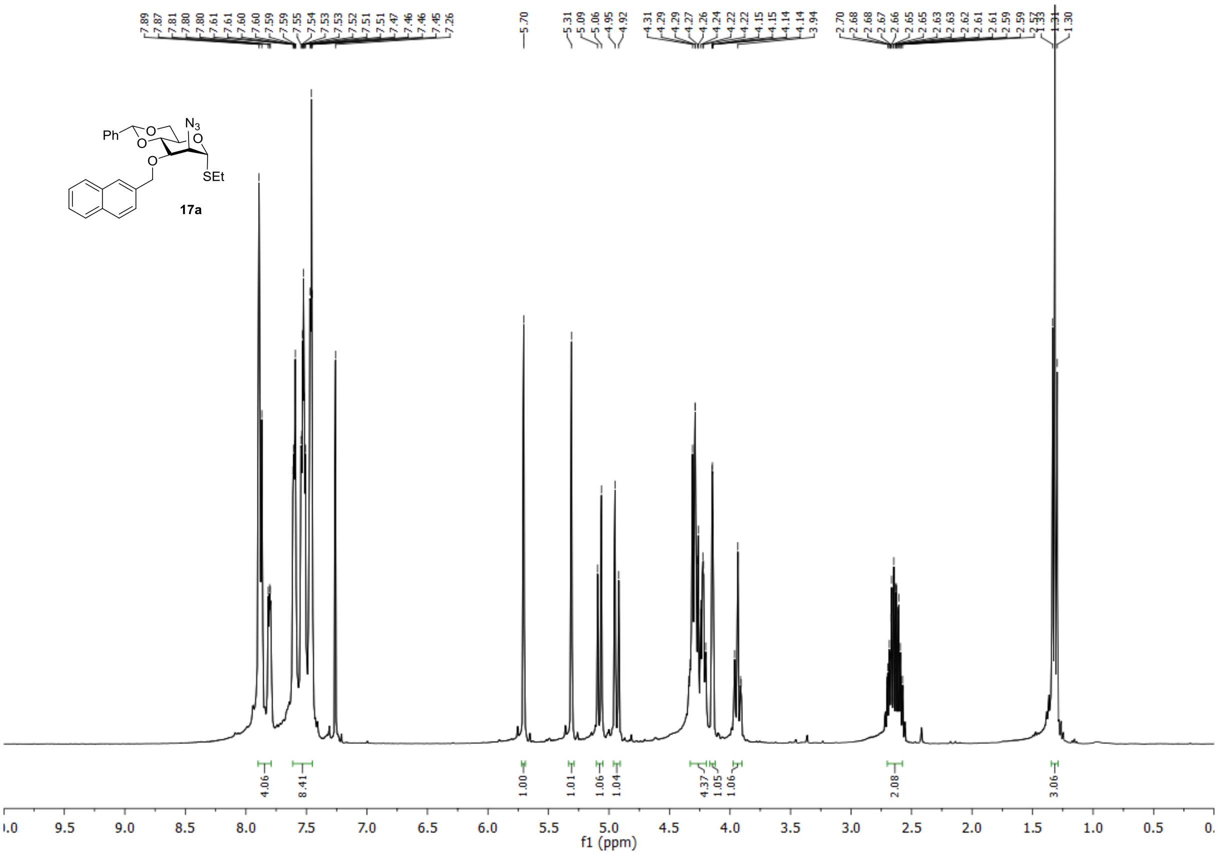
17a
f1 (ppm)

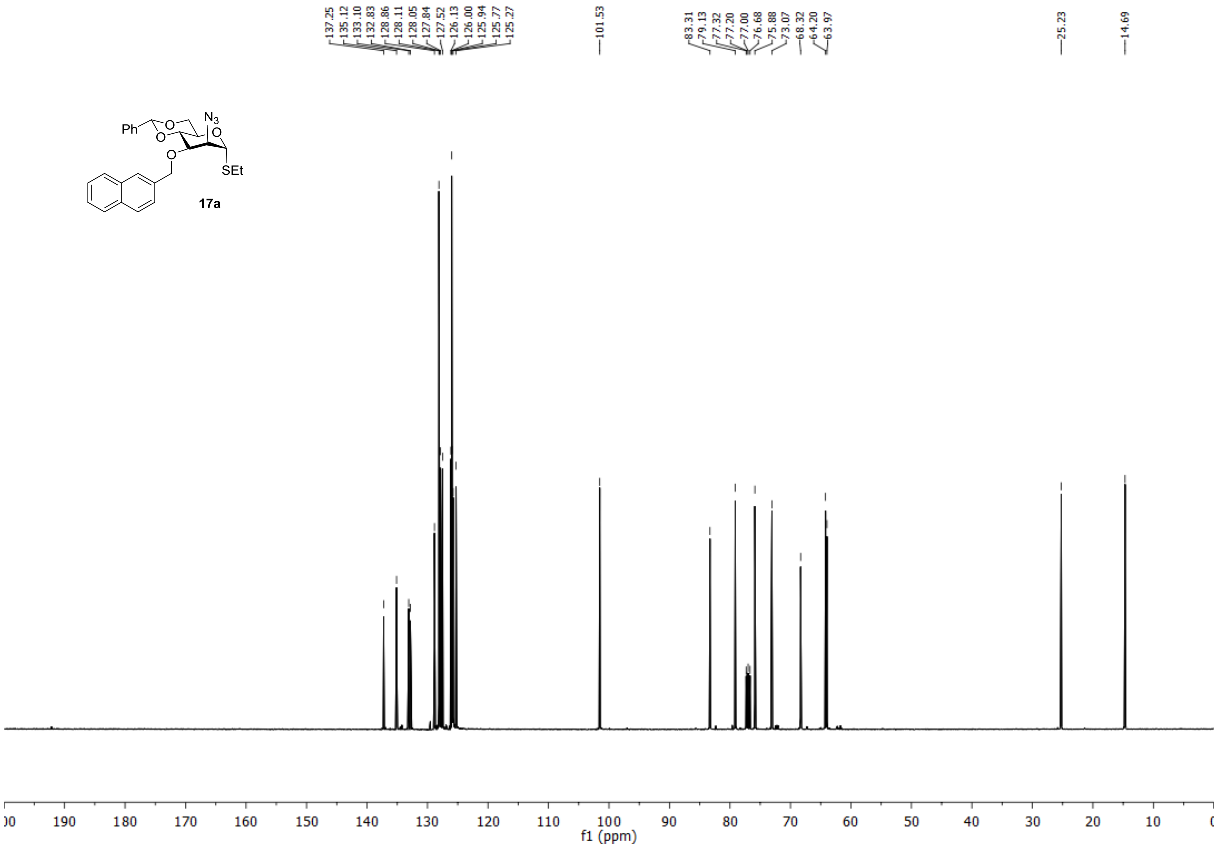
137.25
135.12
133.10
132.83
128.86
128.11
128.05
127.84
127.52
126.13
126.00
125.94
125.77
125.27
101.53
83.31
79.13
77.32
77.20
77.00
76.68
75.88
73.07
68.32
64.20
63.97
25.23
14.69
Ph
O
O
N3
O
O
SEt
17a
f1 (ppm)

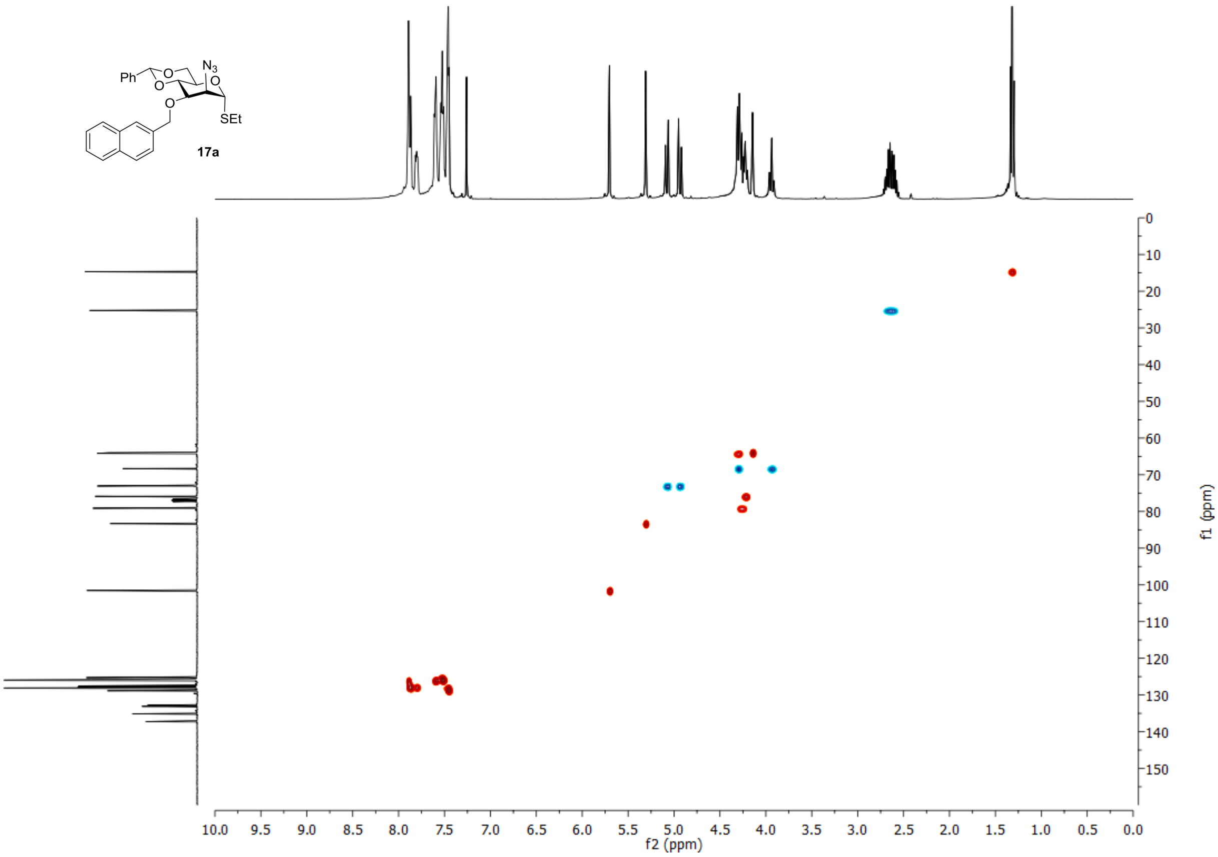
Ph
O
O
N3
O
O
SEt
17a
f2 (ppm)
f1 (ppm)

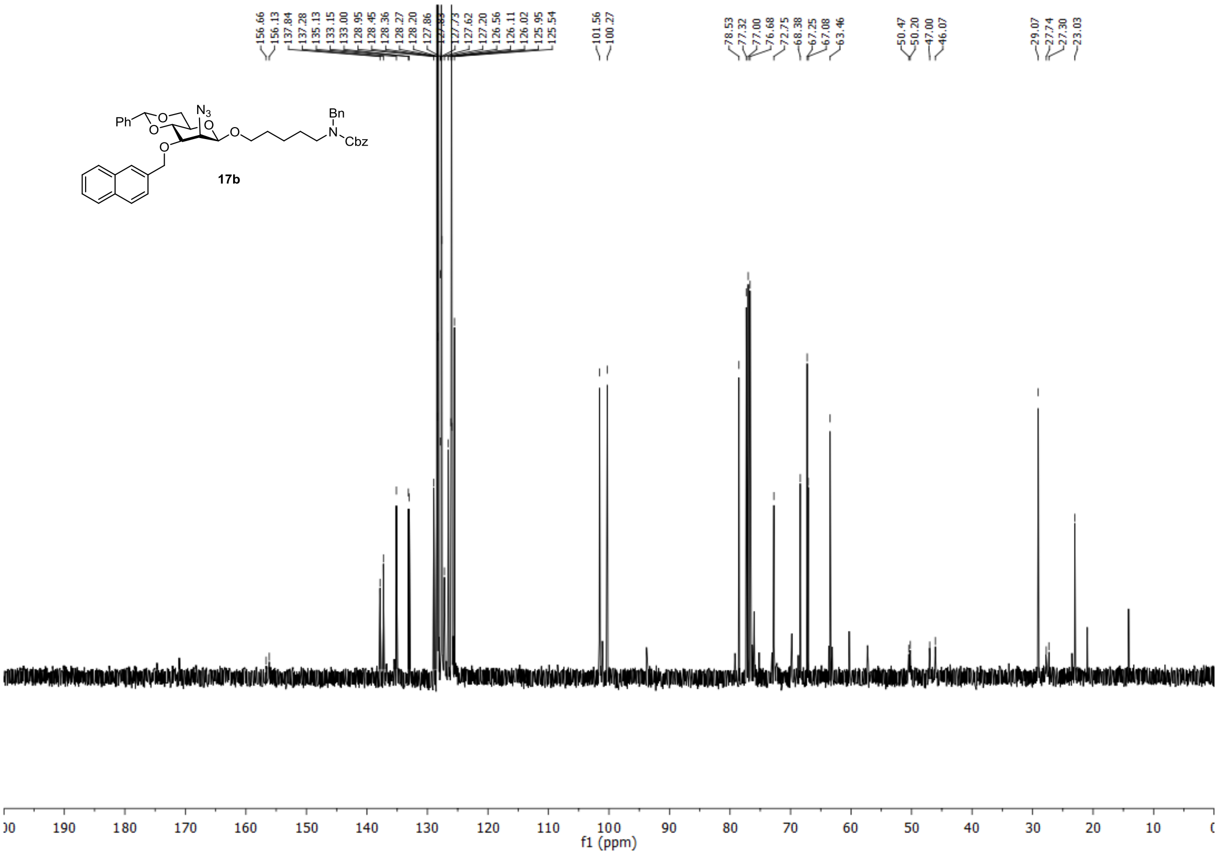
156.66
156.13
137.84
137.28
135.13
133.15
133.00
128.95
128.45
128.36
128.27
128.20
127.86
127.73
127.62
127.20
126.56
126.11
126.02
125.95
125.54
101.56
100.27
78.53
77.32
77.00
76.68
72.75
68.38
67.25
67.08
63.46
50.47
50.20
47.00
46.07
29.07
27.74
27.30
23.03
Ph
N3
Bn
N
Cbz
17b
f1 (ppm)

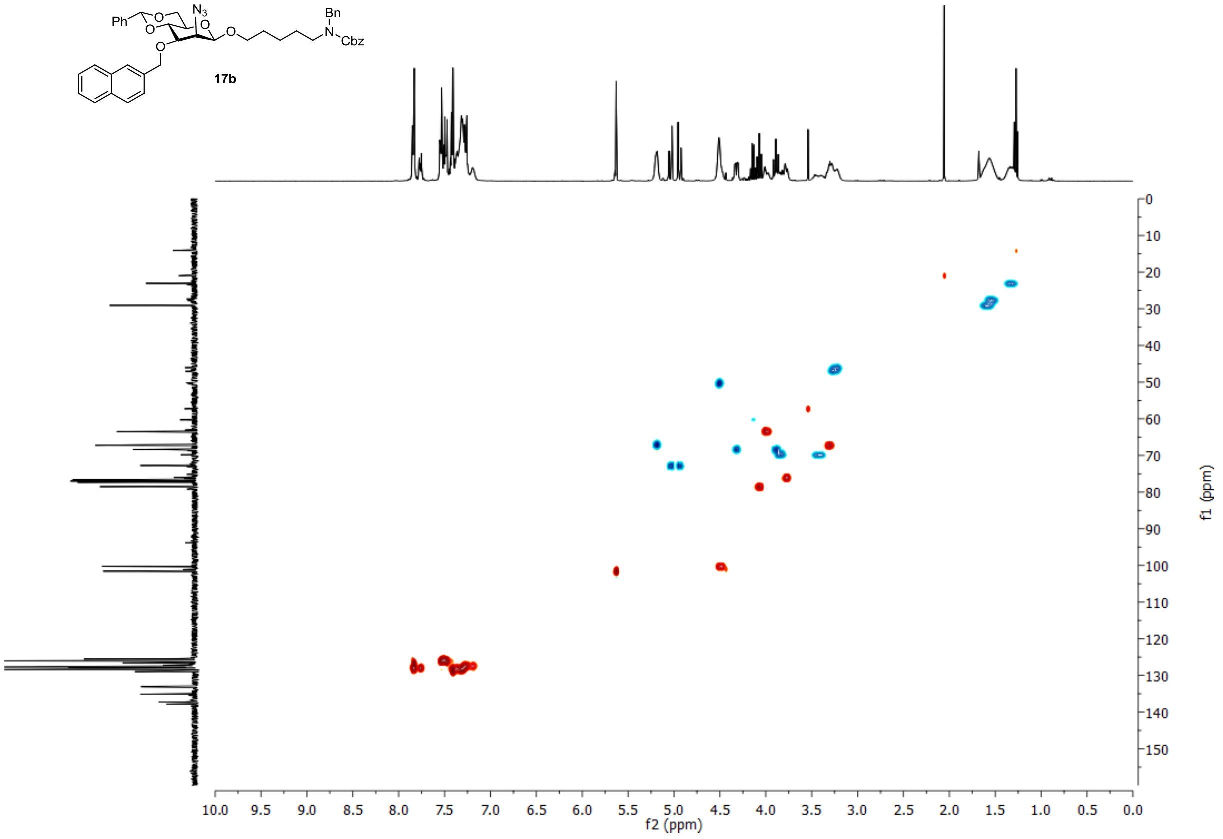
Ph
N3
Bn
N
Cbz
17b
f2 (ppm)
f1 (ppm)

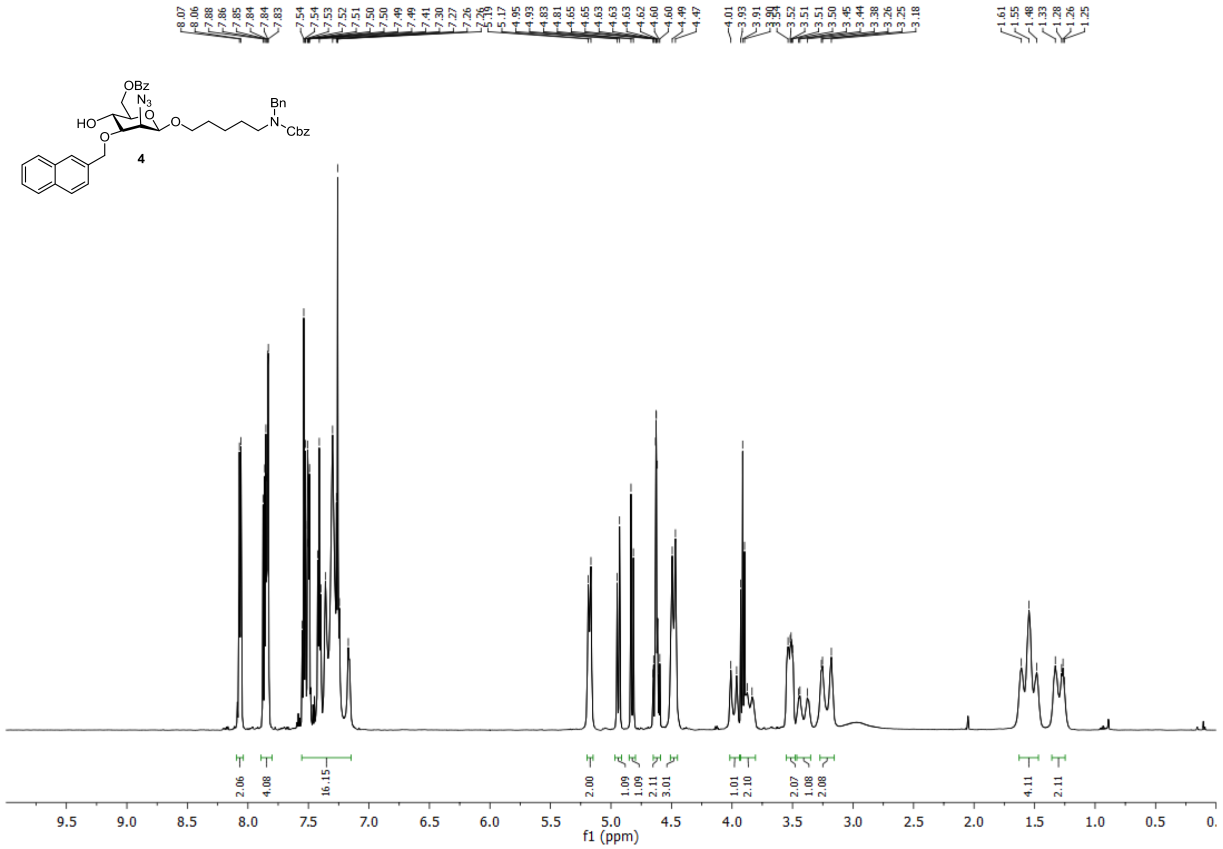
OBz
N_3
HO
O
O
Bn
N
Cbz
4
f1 (ppm)

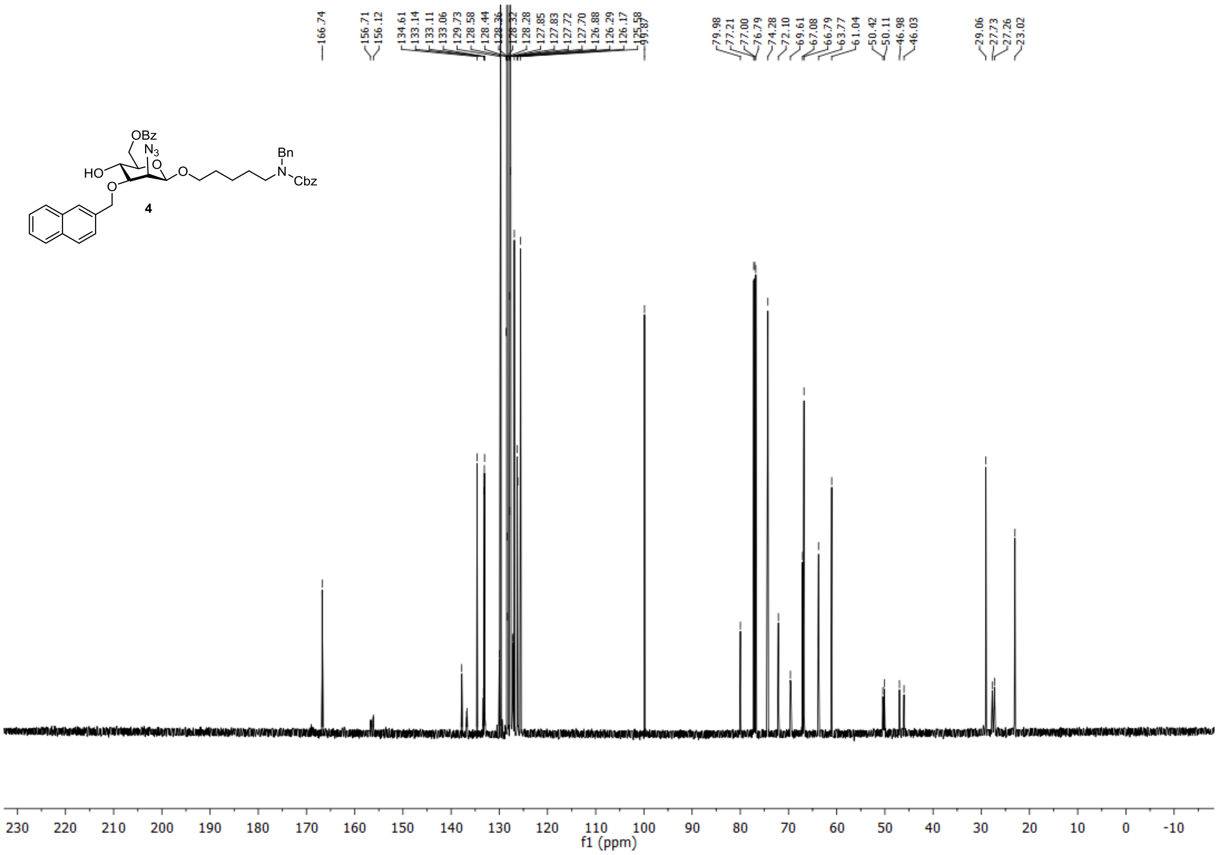
OBz
N_3
HO
O
O
Bn
N
Cbz
4
f1 (ppm)

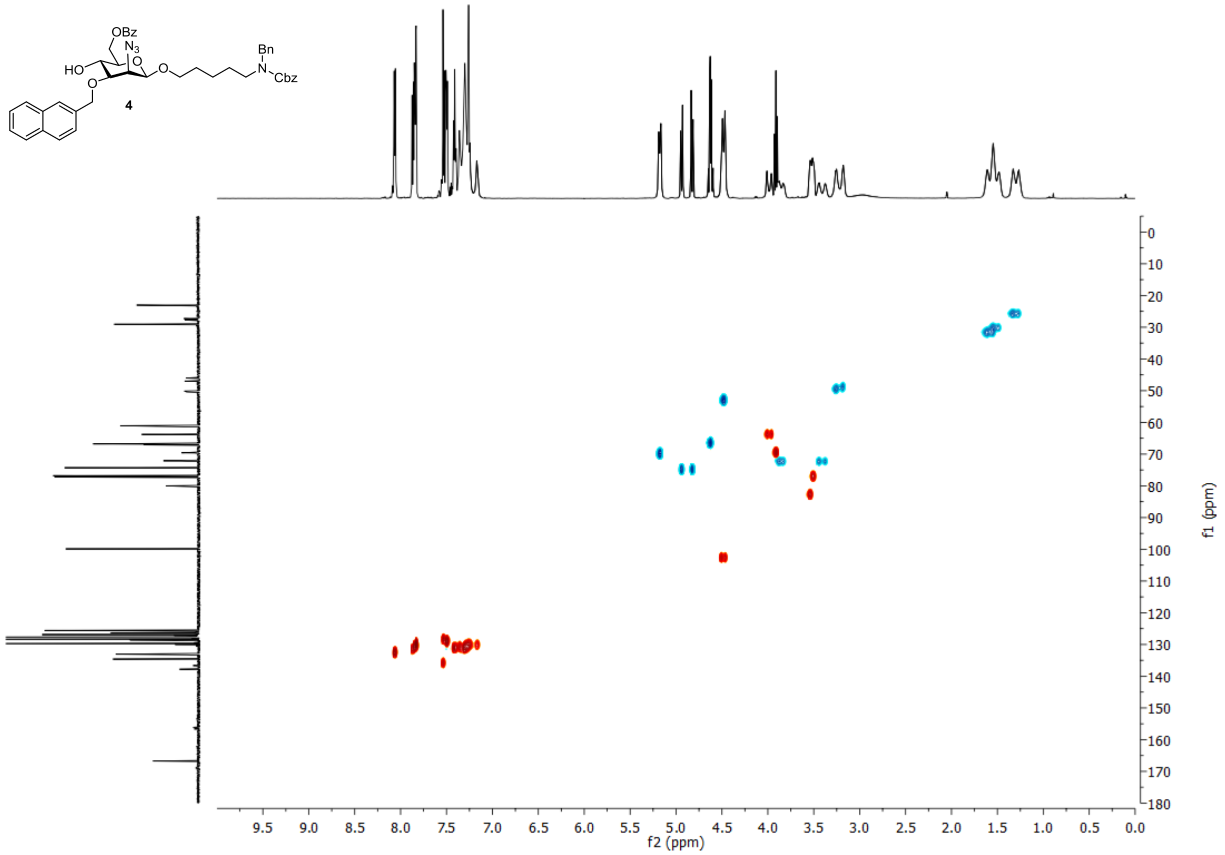
OBz
N3
HO
O
O
Bn
N
Cbz
4
f2 (ppm)
f1 (ppm)

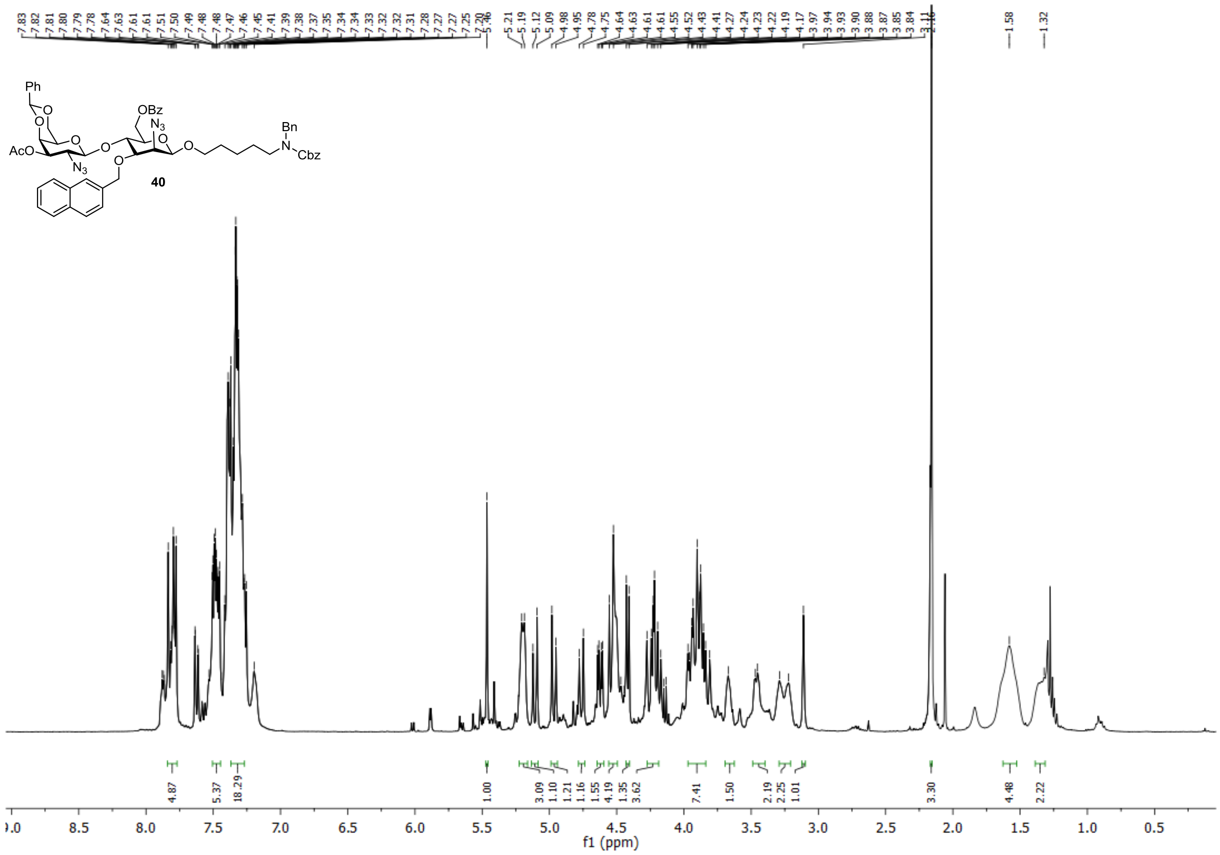
Ph
O
O
OBz
N3
AcO
O
O
N3
O
Bn
N
Cbz
40
f1 (ppm)

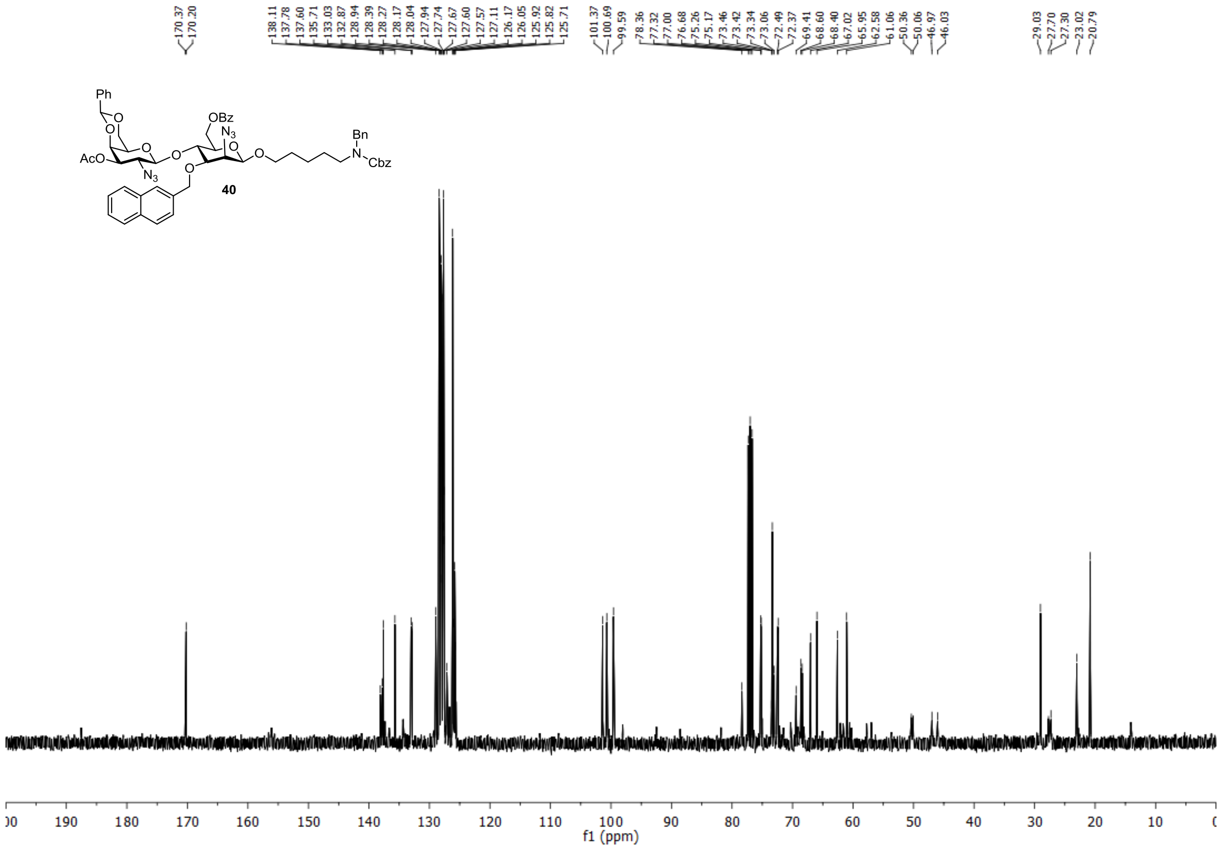
170.37
170.20
138.11
137.78
137.60
135.71
133.03
132.87
128.94
128.39
128.27
128.17
128.04
127.94
127.74
127.67
127.60
127.57
127.11
126.17
126.05
125.92
125.82
125.71
101.37
100.69
99.59
78.36
77.32
77.00
76.68
75.26
75.17
73.46
73.42
73.34
73.06
72.49
72.37
69.41
68.60
68.40
67.02
65.95
62.58
61.06
50.36
50.06
46.97
46.03
29.03
27.70
27.30
23.02
20.79
Ph
OBz
N3
AcO
N3
Bn
N
Cbz
40
f1 (ppm)

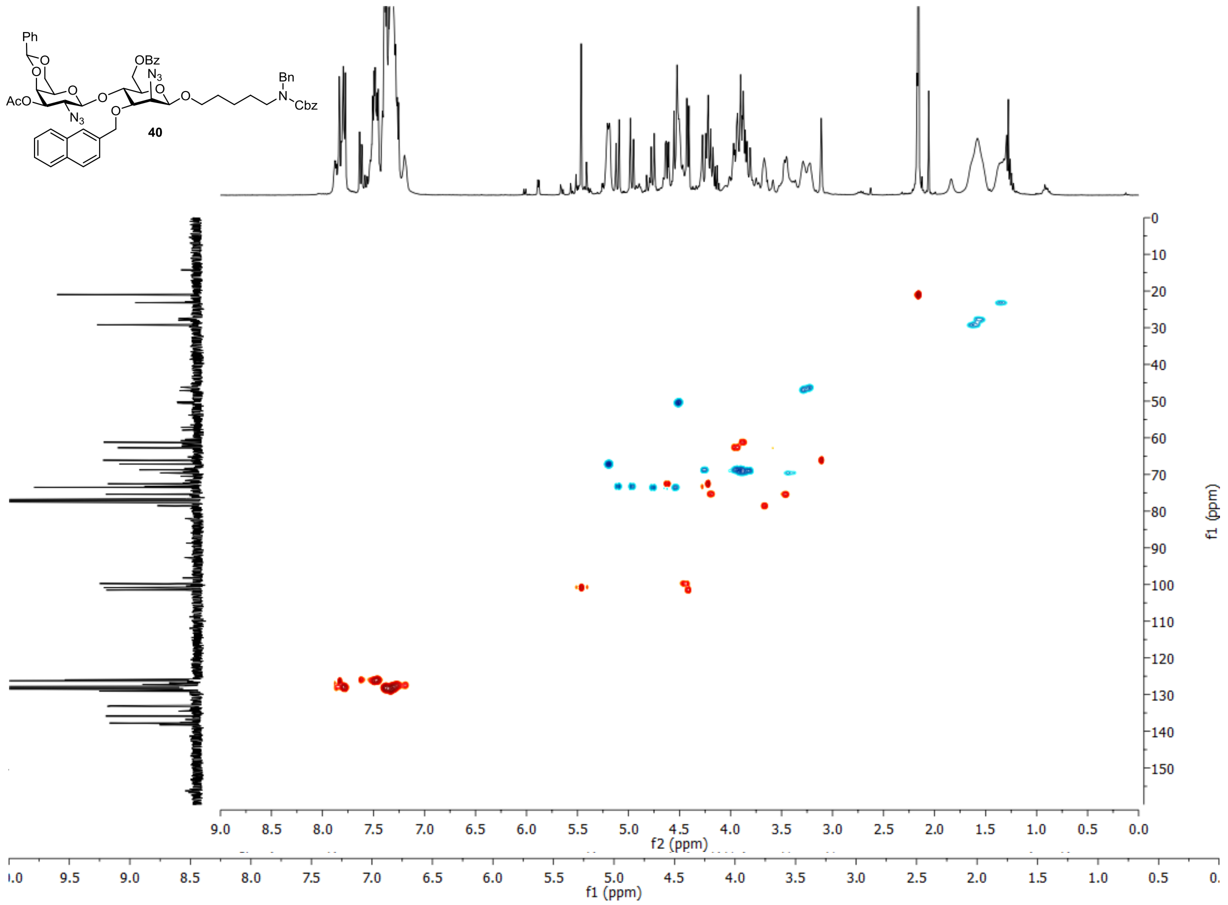
Ph
OBz
N3
AcO
N3
Bn
N
Cbz
40
f2 (ppm)
f1 (ppm)

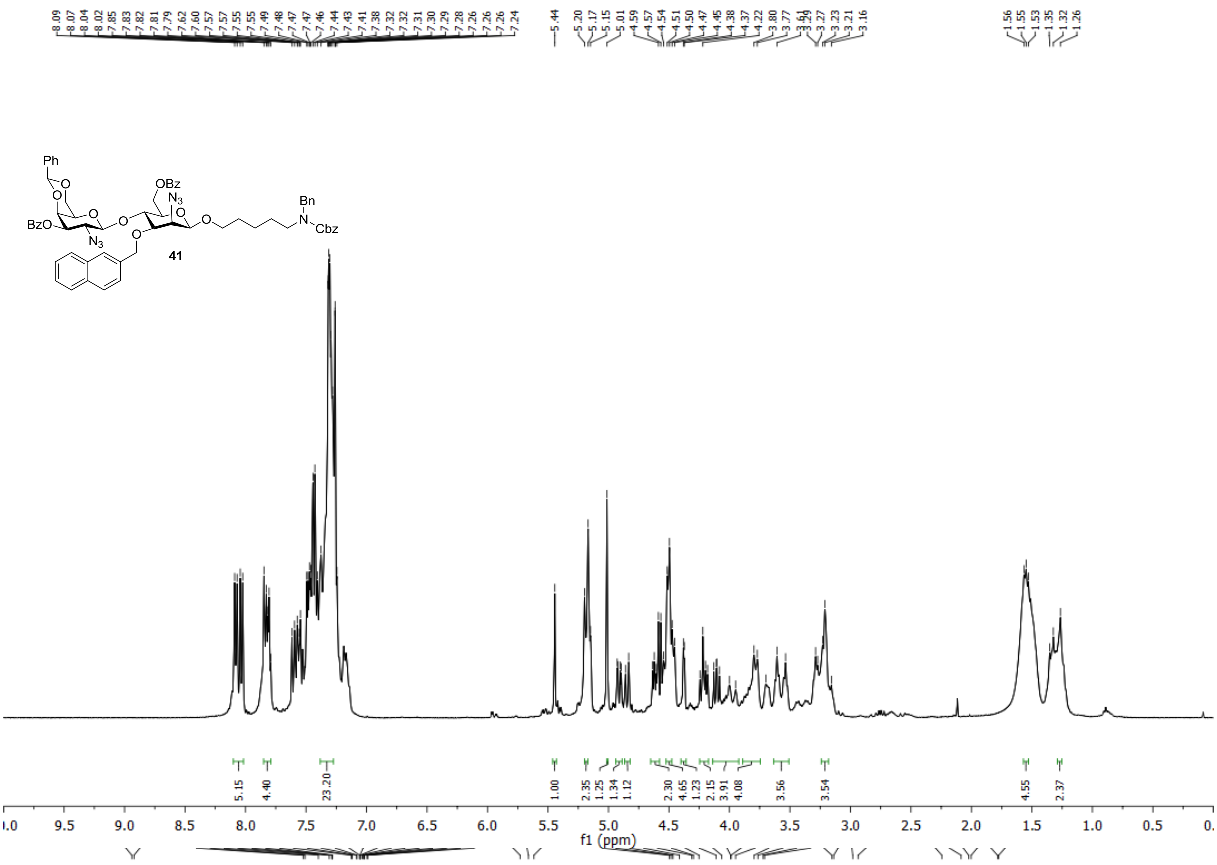

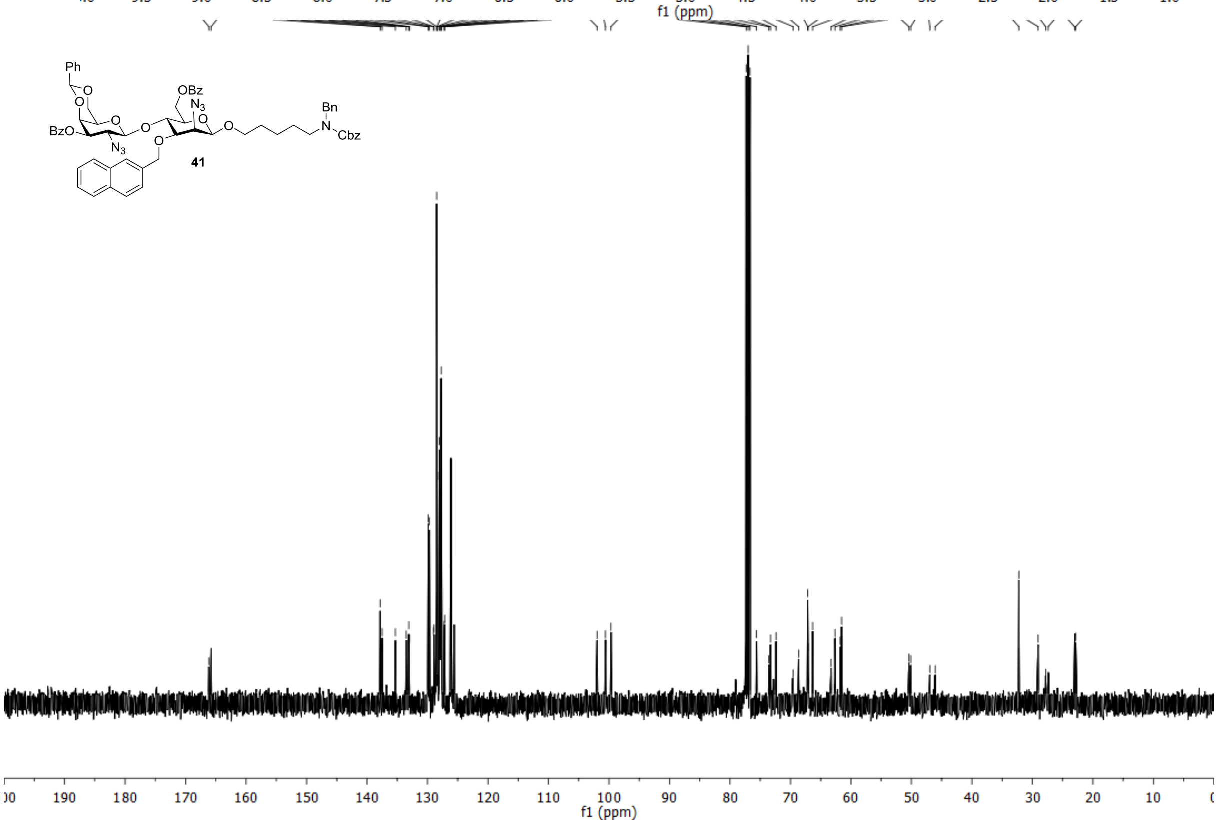

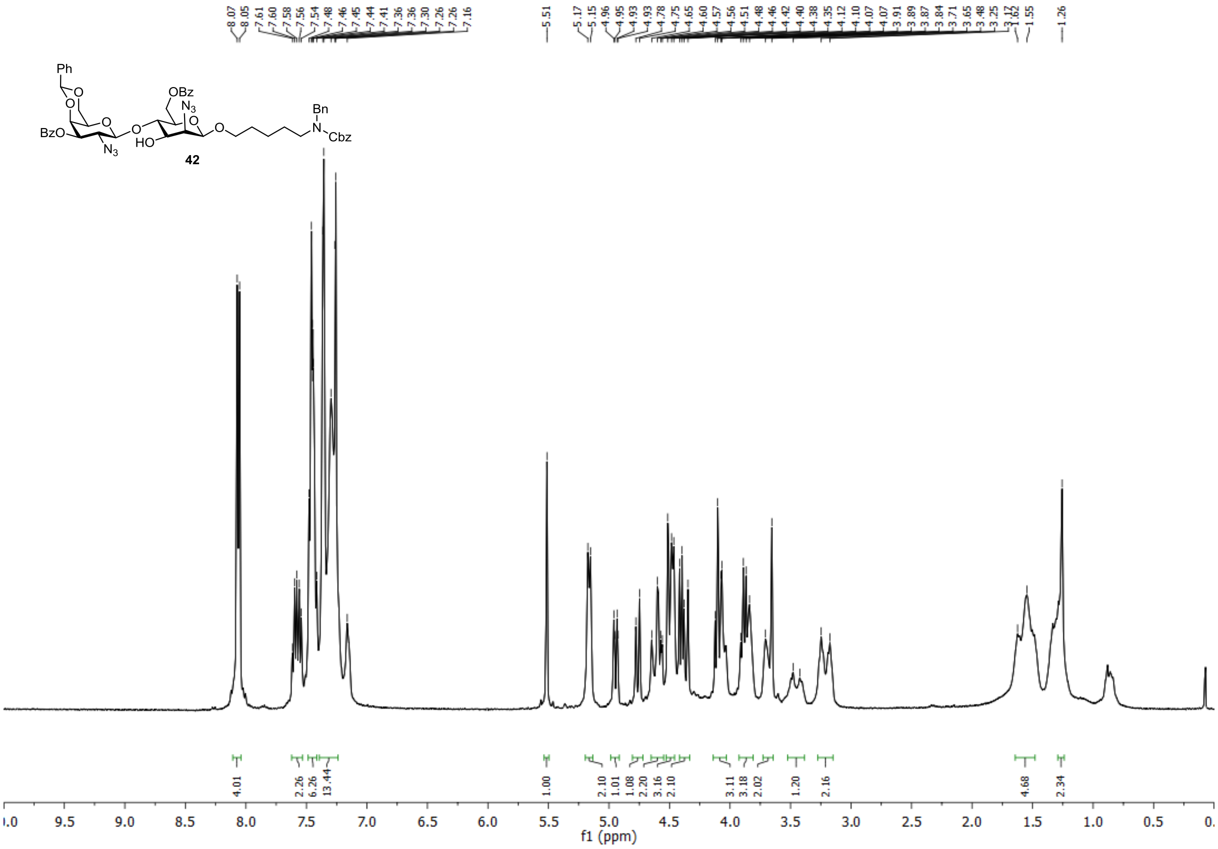

Ph
BzO
N3
OBz
HO
Bn
N
Cbz
42
f1 (ppm)

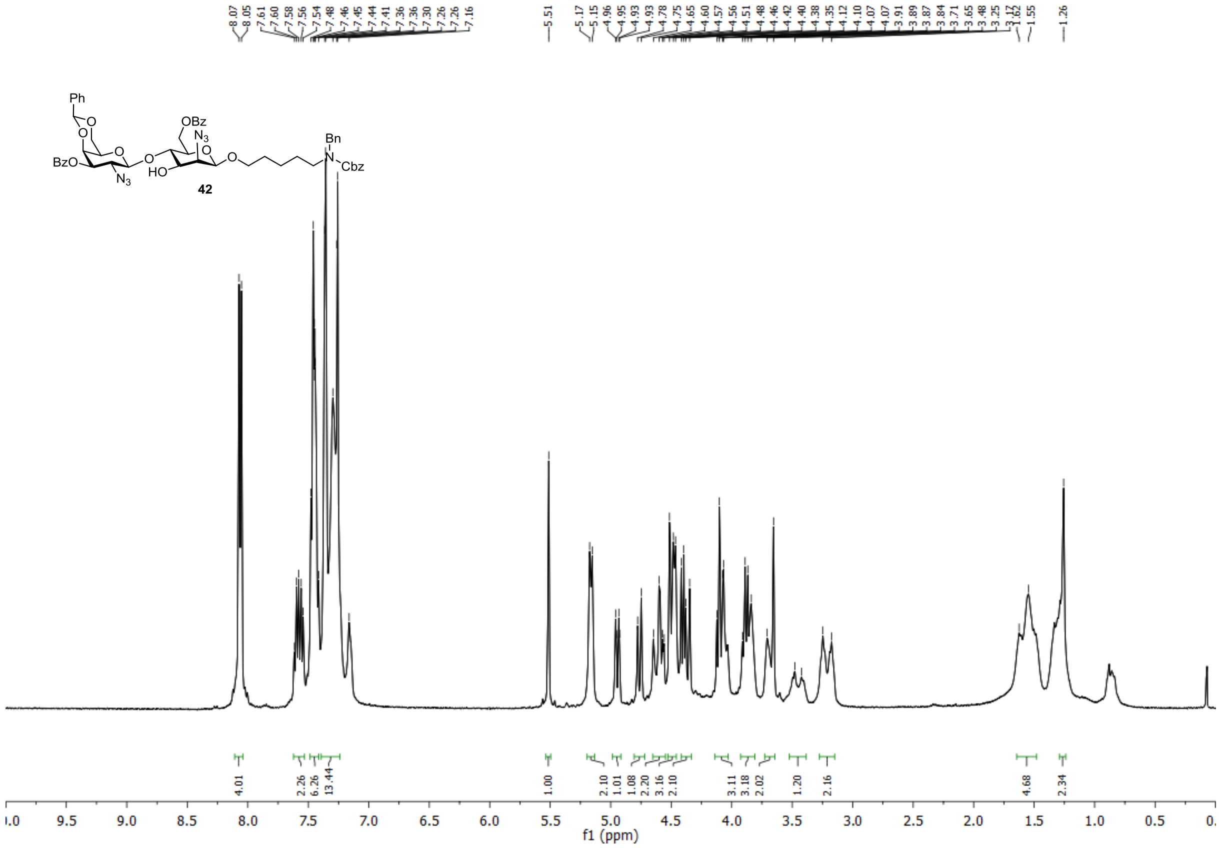

Ph
BzO
N3
OBz
HO
Bn
N
Cbz
42
f1 (ppm)

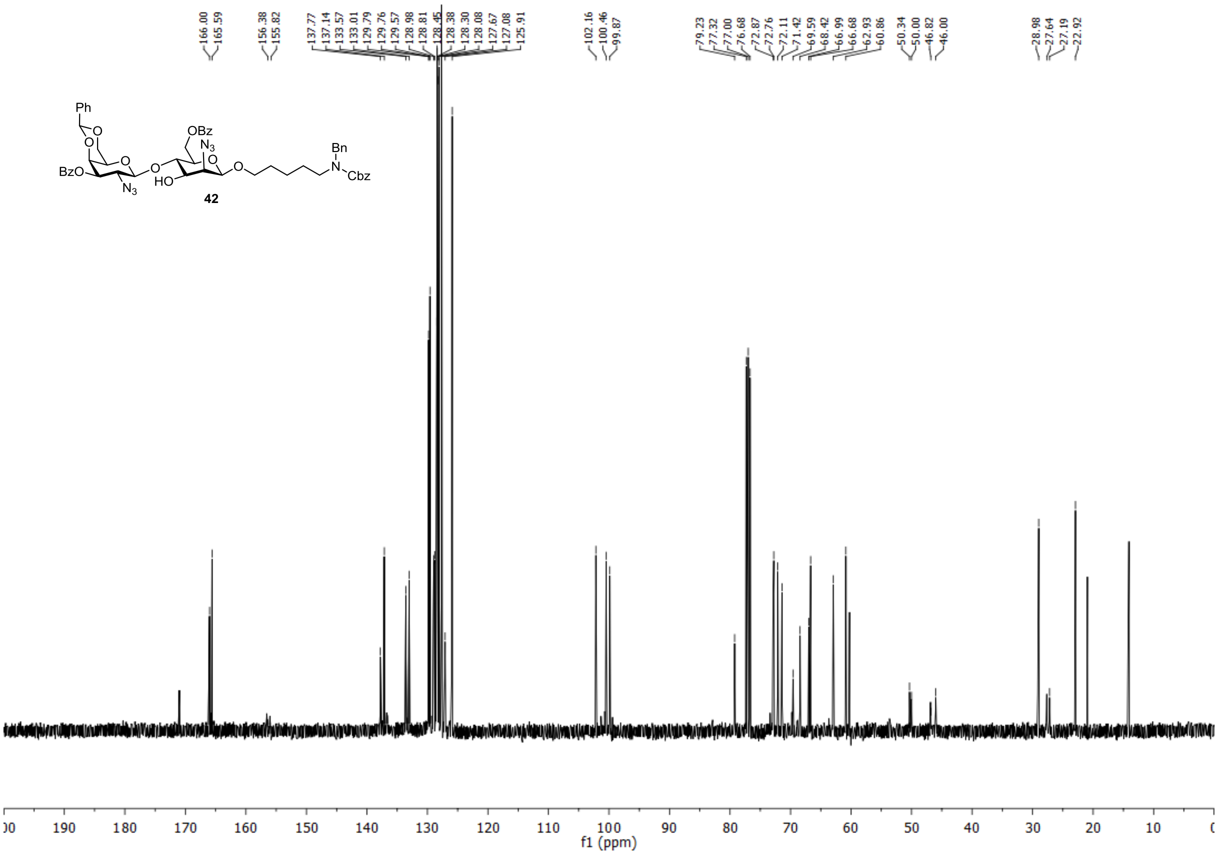
166.00
165.59
156.38
155.82
137.77
137.14
133.57
133.01
129.79
129.76
129.57
128.98
128.81
128.45
128.38
128.30
128.08
127.67
127.08
125.91
102.16
100.46
99.87
79.23
77.32
77.00
76.68
72.87
72.76
72.11
71.42
69.59
68.42
66.99
66.68
62.93
60.86
50.34
50.00
46.82
46.00
28.98
27.64
27.19
22.92
Ph
O
O
BzO
O
N_3
O
OBz
N_3
O
HO
O
Bn
N
Cbz
42
f1 (ppm)

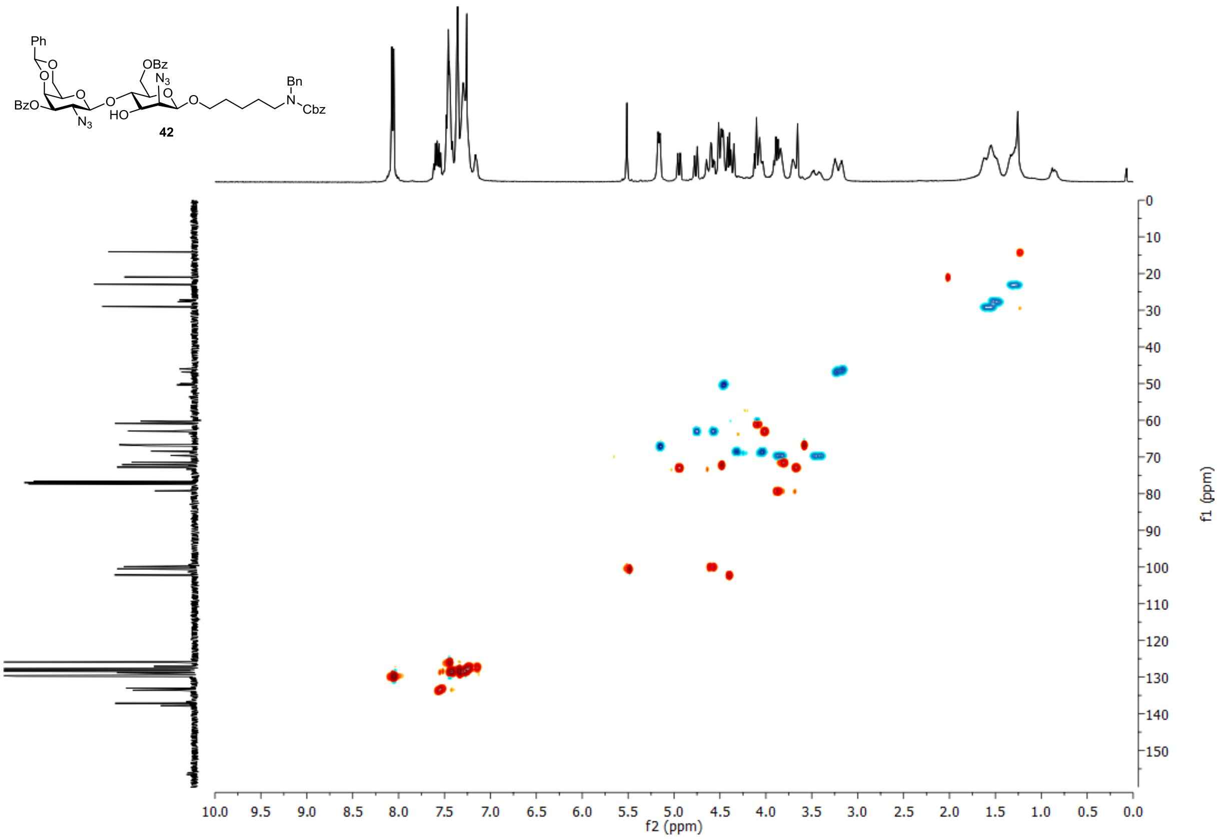
Ph
O
O
BzO
O
N_3
O
OBz
N_3
O
HO
O
Bn
N
Cbz
42
f2 (ppm)
f1 (ppm)

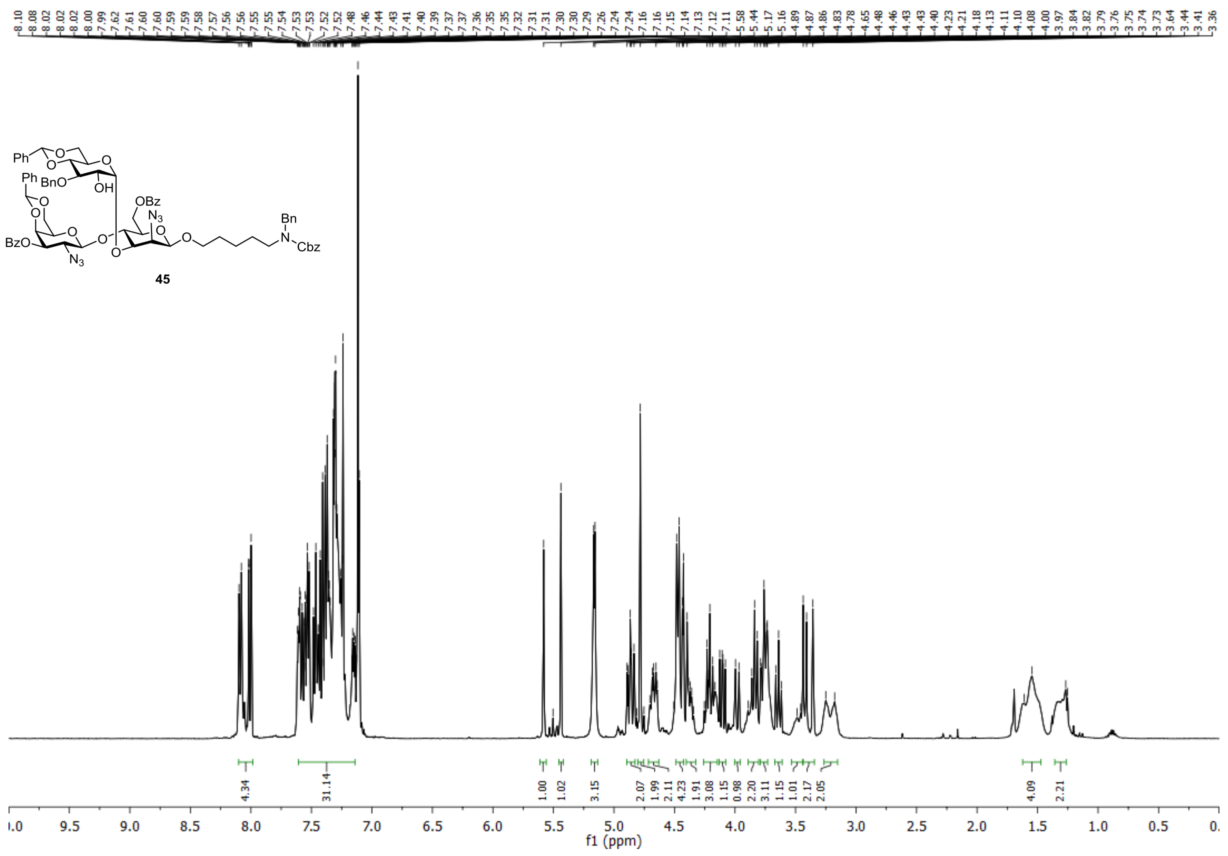
45
f1 (ppm)

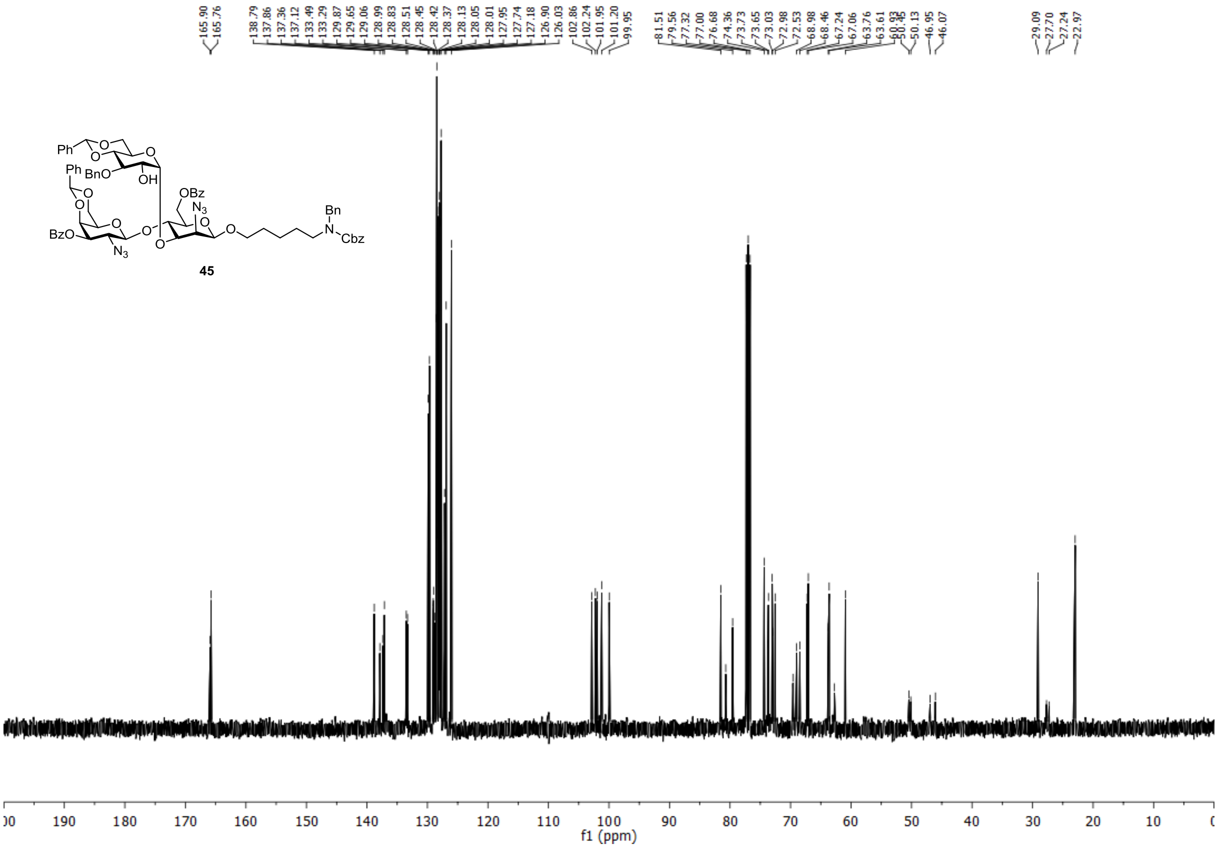

45

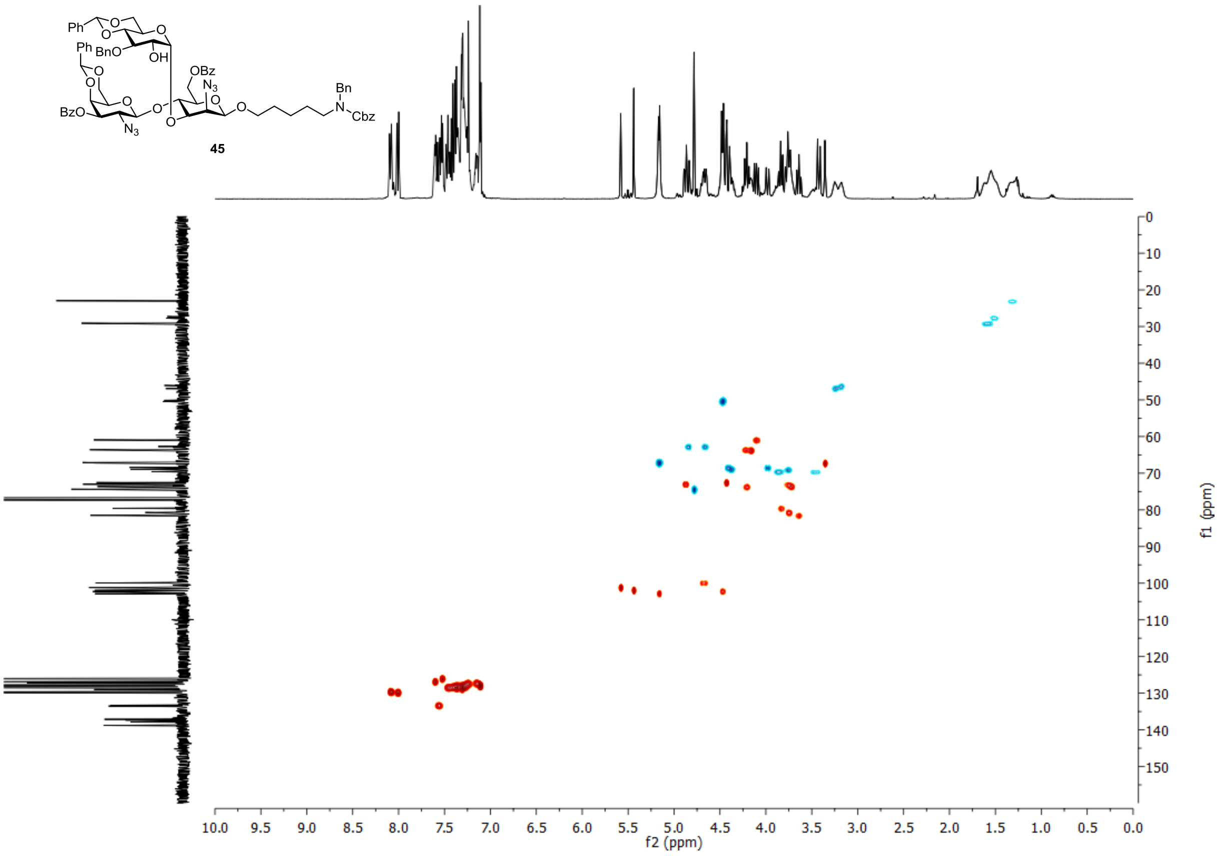

45

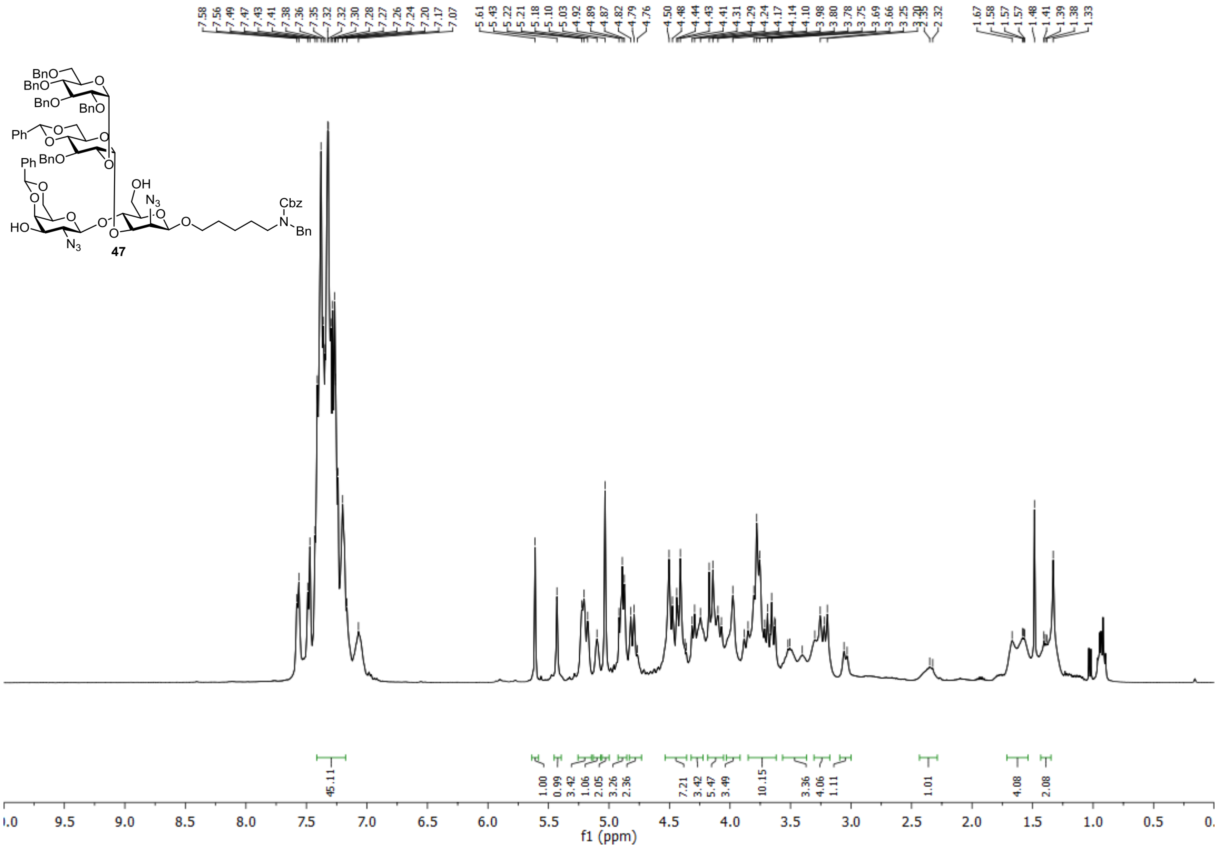

7.58 7.56 7.49 7.47 7.43 7.41 7.38 7.36 7.35 7.32 7.32 7.30 7.28 7.27 7.26 7.24 7.20 7.17 7.07
5.61 5.43 5.22 5.21 5.18 5.10 5.03 4.92 4.89 4.87 4.82 4.79 4.76
4.50 4.48 4.44 4.43 4.41 4.31 4.29 4.24 4.17 4.14 4.10 3.98 3.80 3.78 3.75 3.69 3.66 3.30 3.25 2.35 2.32
1.67 1.58 1.57 1.57 1.48 1.41 1.39 1.38 1.33
BnO BnO BnO BnO Ph O BnO Ph O HO N3 OH N3 O Cbz N Bn
47
45.11
1.00 0.99 3.42 1.06 2.05 3.26 2.36 7.21 3.42 5.47 3.49 10.15 3.36 4.06 1.11 1.01 4.08 2.08
f1 (ppm)

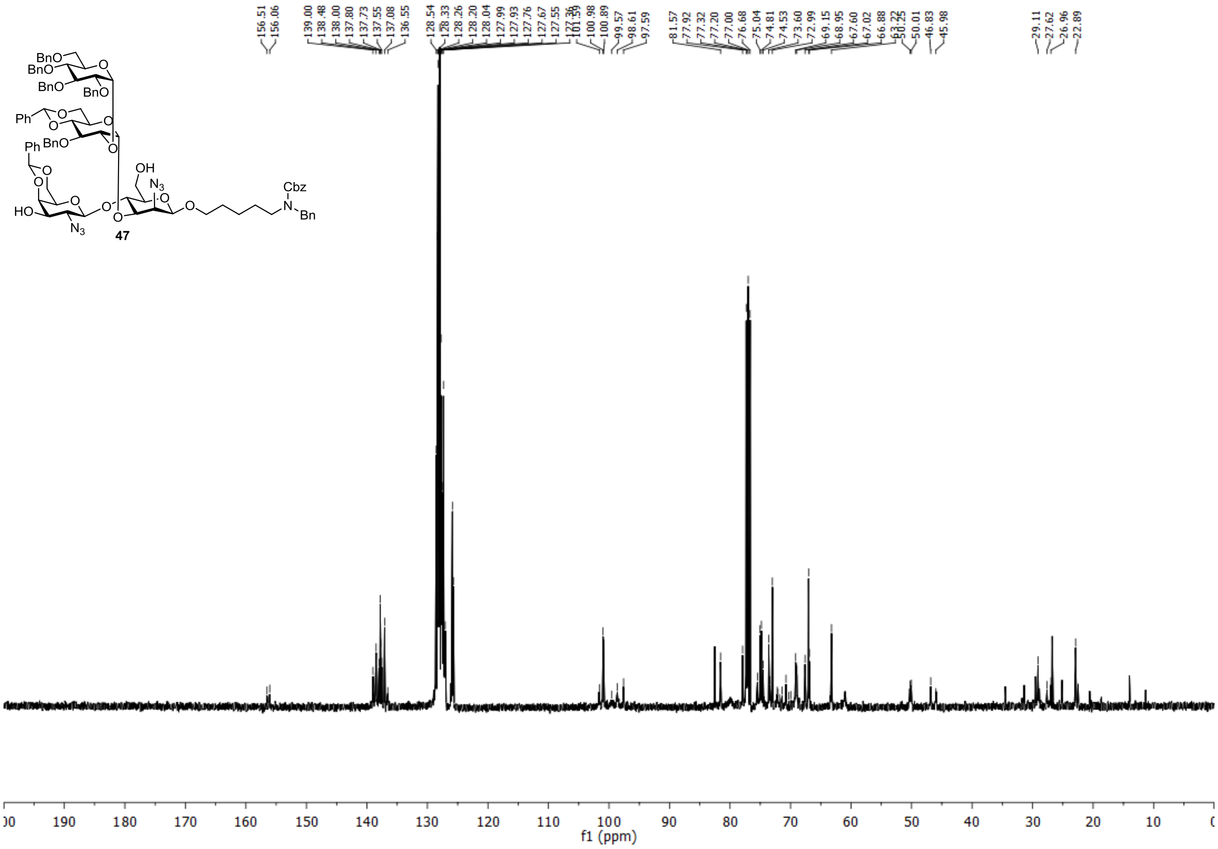

156.51 156.06
139.00 138.48 138.00 137.80 137.73 137.55 137.08 136.55
128.54 128.33 128.26 128.20 128.04 127.99 127.93 127.76 127.67 127.55 101.50 100.98 100.89 99.57 98.61 97.59
81.57 77.92 77.32 77.20 77.00 76.68 75.04 74.81 74.53 73.60 72.99 69.15 68.95 67.60 67.02 66.88 63.22 50.25 50.01 46.83 45.98
29.11 27.62 26.96 22.89
BnO BnO BnO BnO Ph O BnO Ph O HO N3 OH N3 O Cbz N Bn
47
f1 (ppm)

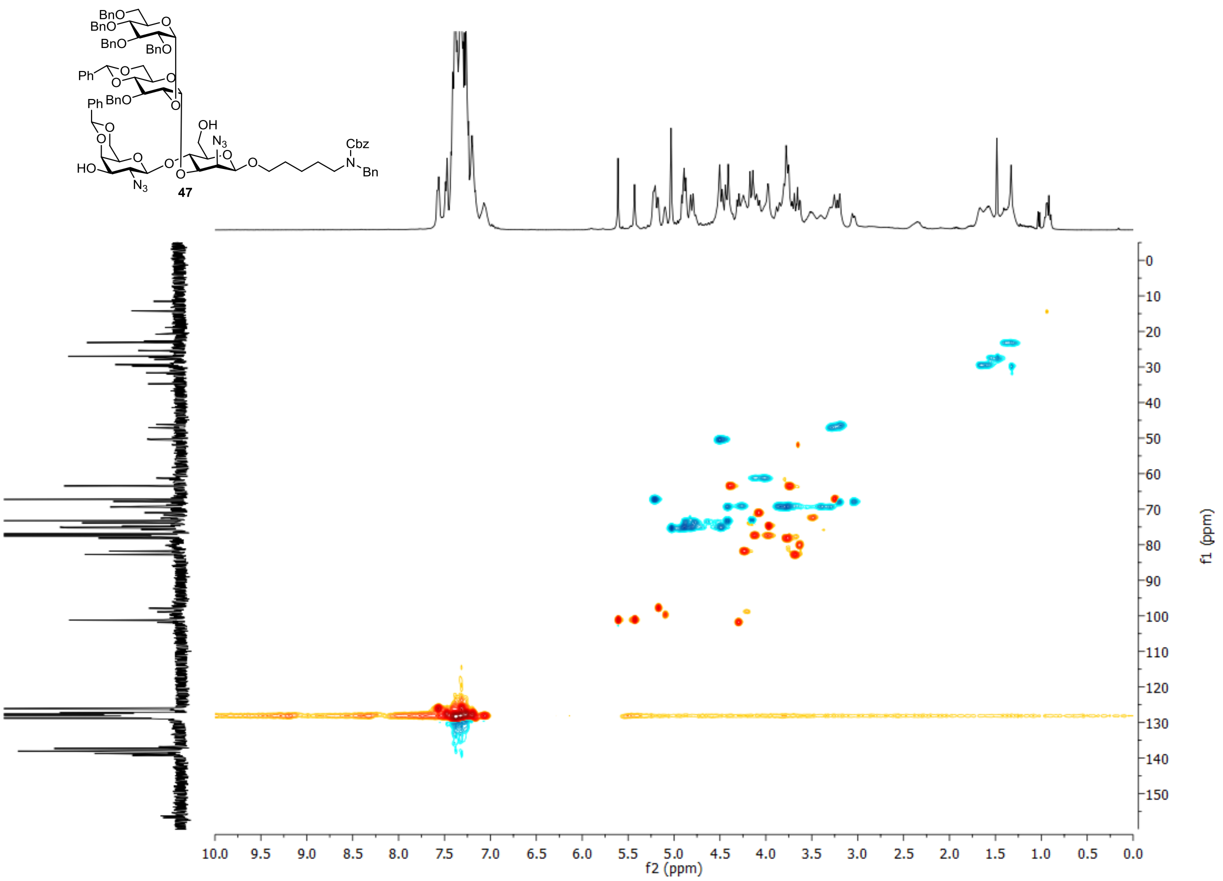
BnO
BnO
BnO
BnO
Ph
O
BnO
Ph
OH
N3
HO
N3
Cbz
N
Bn
47
f2 (ppm)
f1 (ppm)

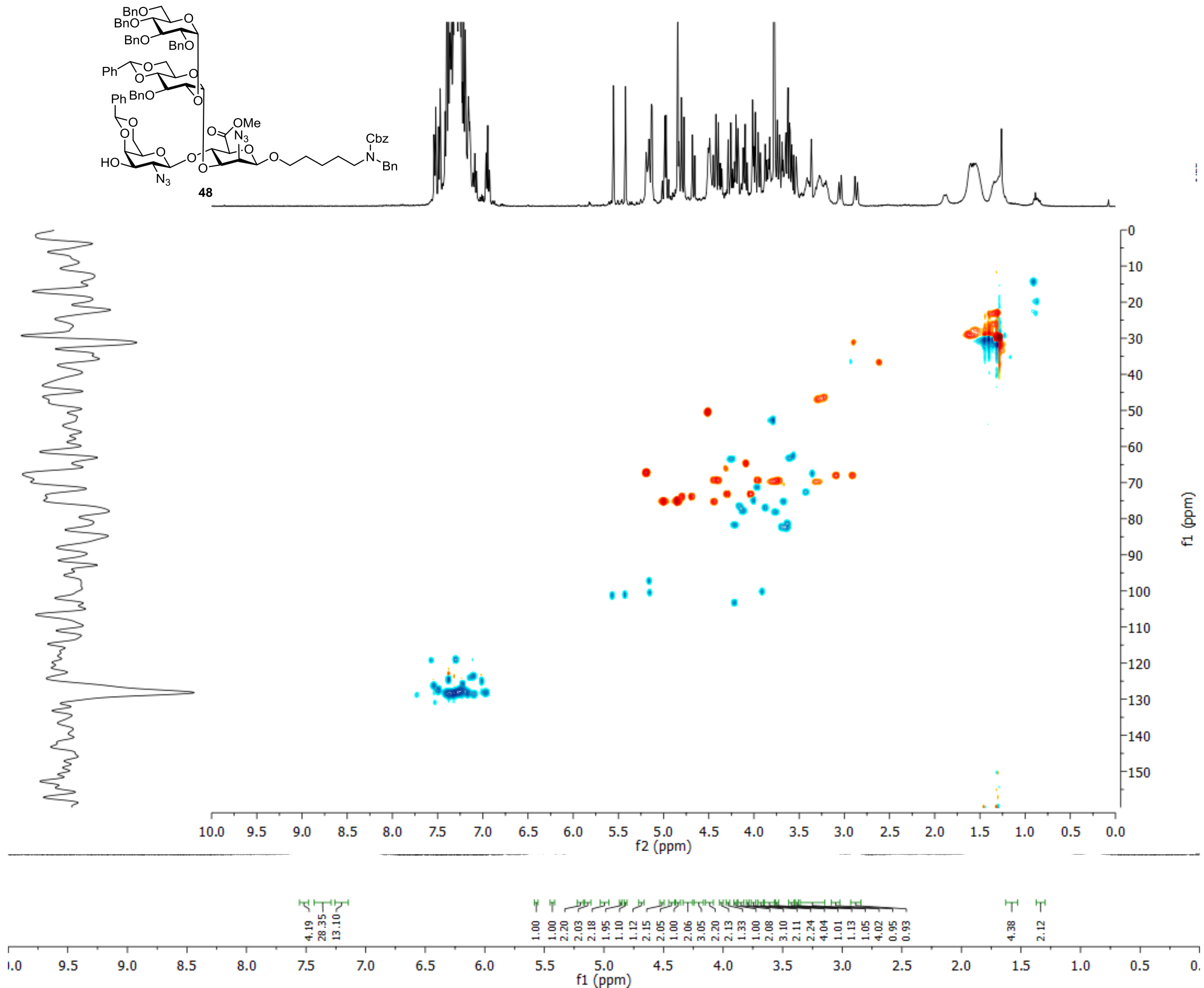
BnO
BnO
BnO
BnO
Ph
BnO
Ph
OMe
N3
HO
N3
Cbz
N
Bn
48
f2 (ppm)
f1 (ppm)

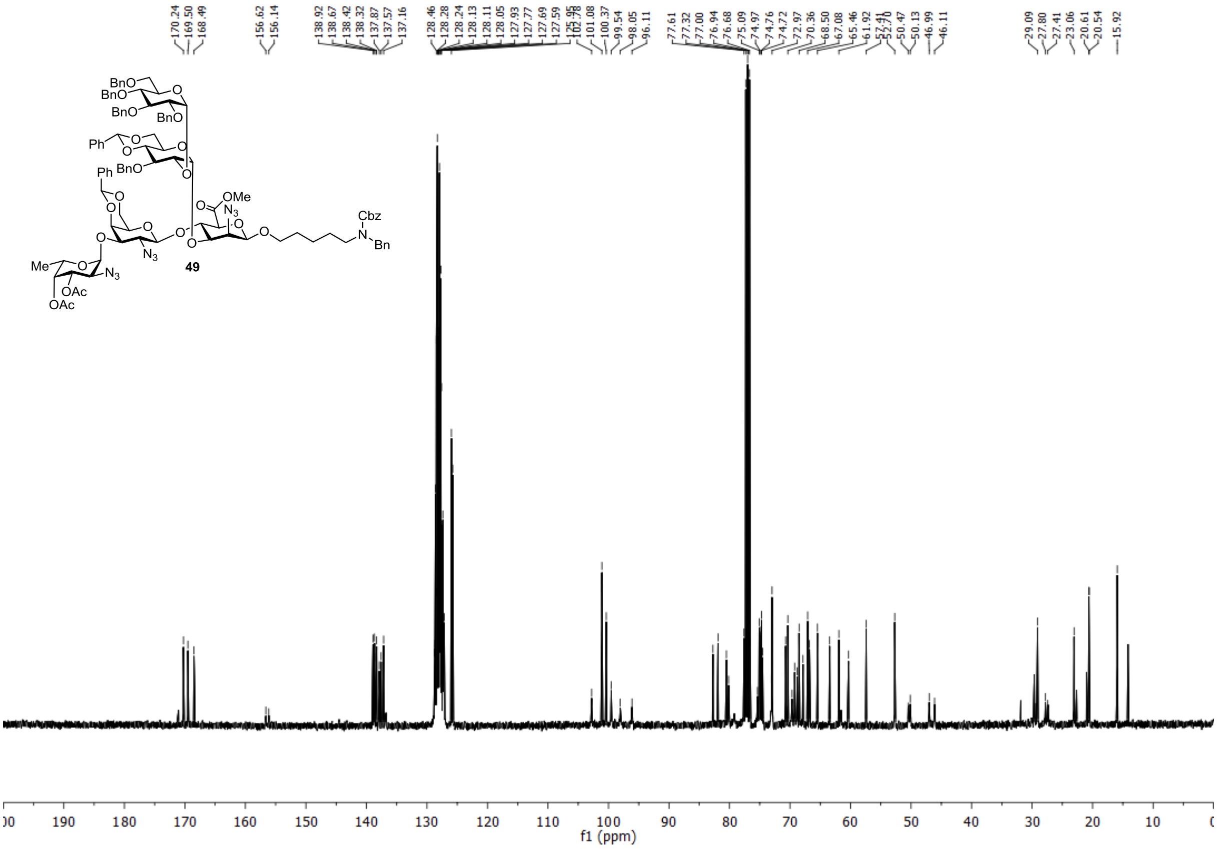
BnO
BnO
BnO
BnO
Ph
BnO
Ph
OMe
N3
Cbz
N
Bn
Me
N3
N3
OAc
OAc
49
f1 (ppm)

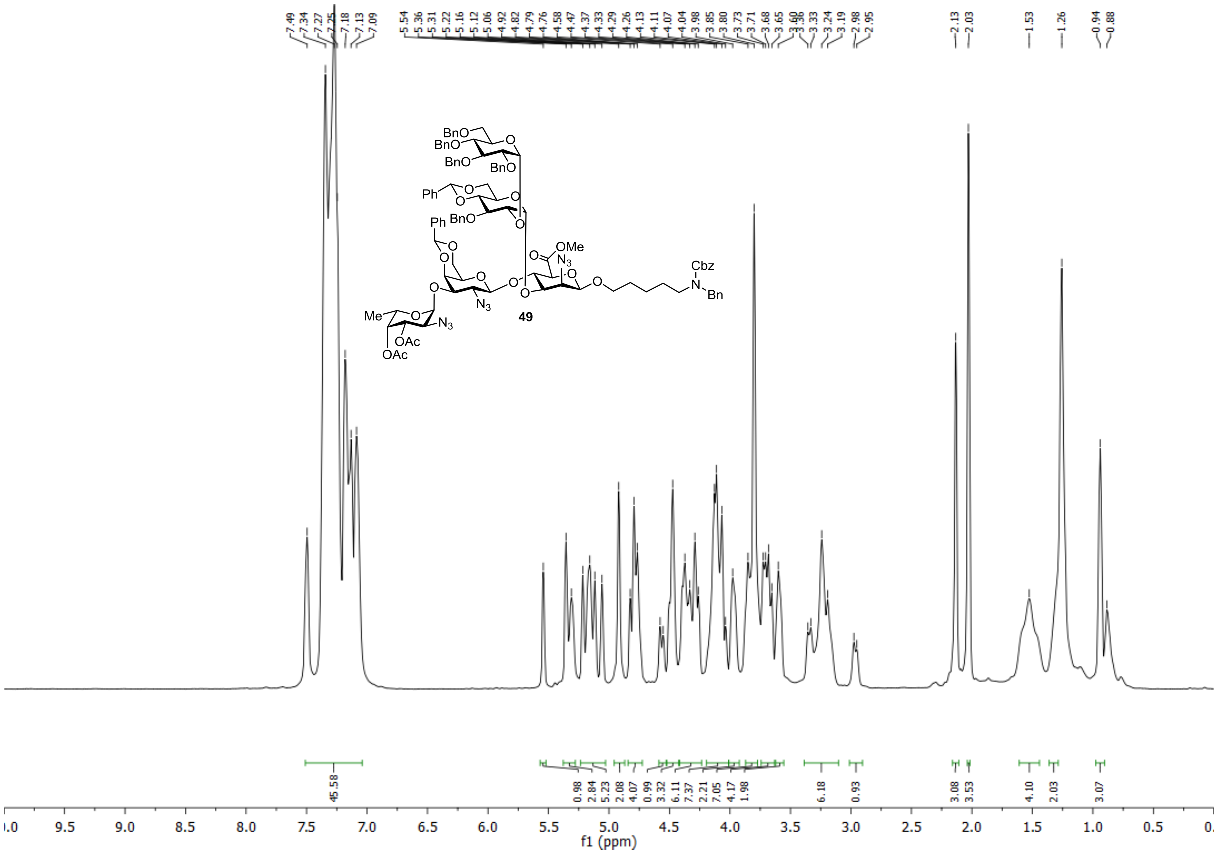
BnO
BnO
BnO
BnO
Ph
BnO
Ph
OMe
N3
Cbz
N
Bn
Me
N3
N3
OAc
OAc
49
f1 (ppm)

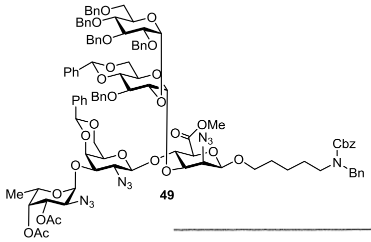
BnO
BnO
BnO
BnO
O
Ph
O
O
O
BnO
O
Ph
O
O
O
O
N3
O
O
OMe
N3
O
O
O
Cbz
N
Bn
Me
O
N3
OAc
OAc
49

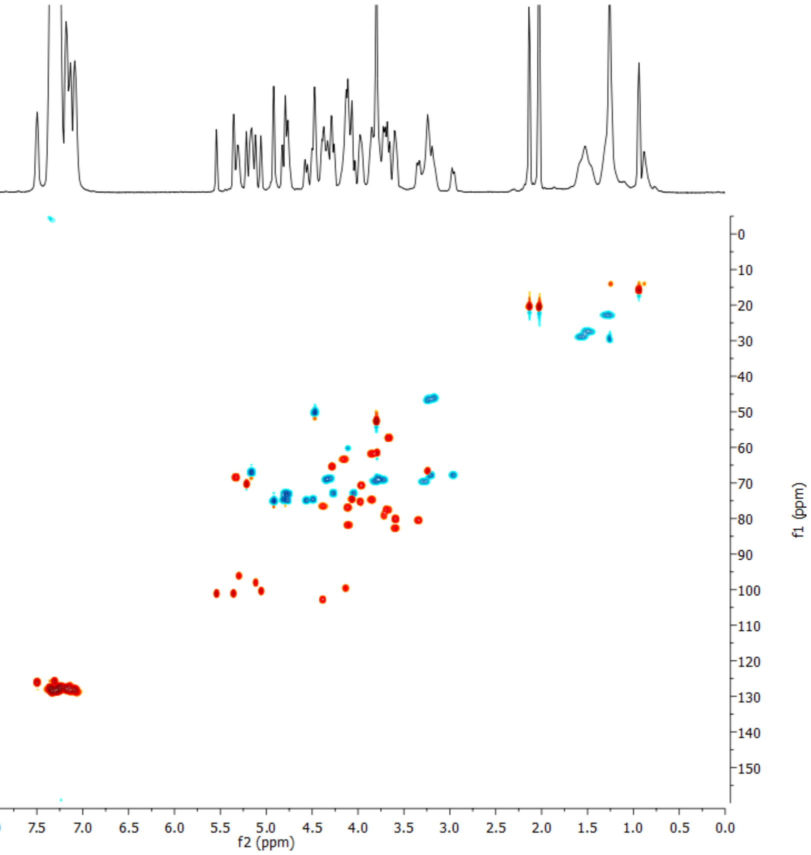
10.0
9.5
9.0
8.5
8.0
7.5
7.0
6.5
6.0
5.5
5.0
4.5
4.0
3.5
3.0
2.5
2.0
1.5
1.0
0.5
0.0
f2 (ppm)
0
10
20
30
40
50
60
70
80
90
100
110
120
130
140
150
f1 (ppm)

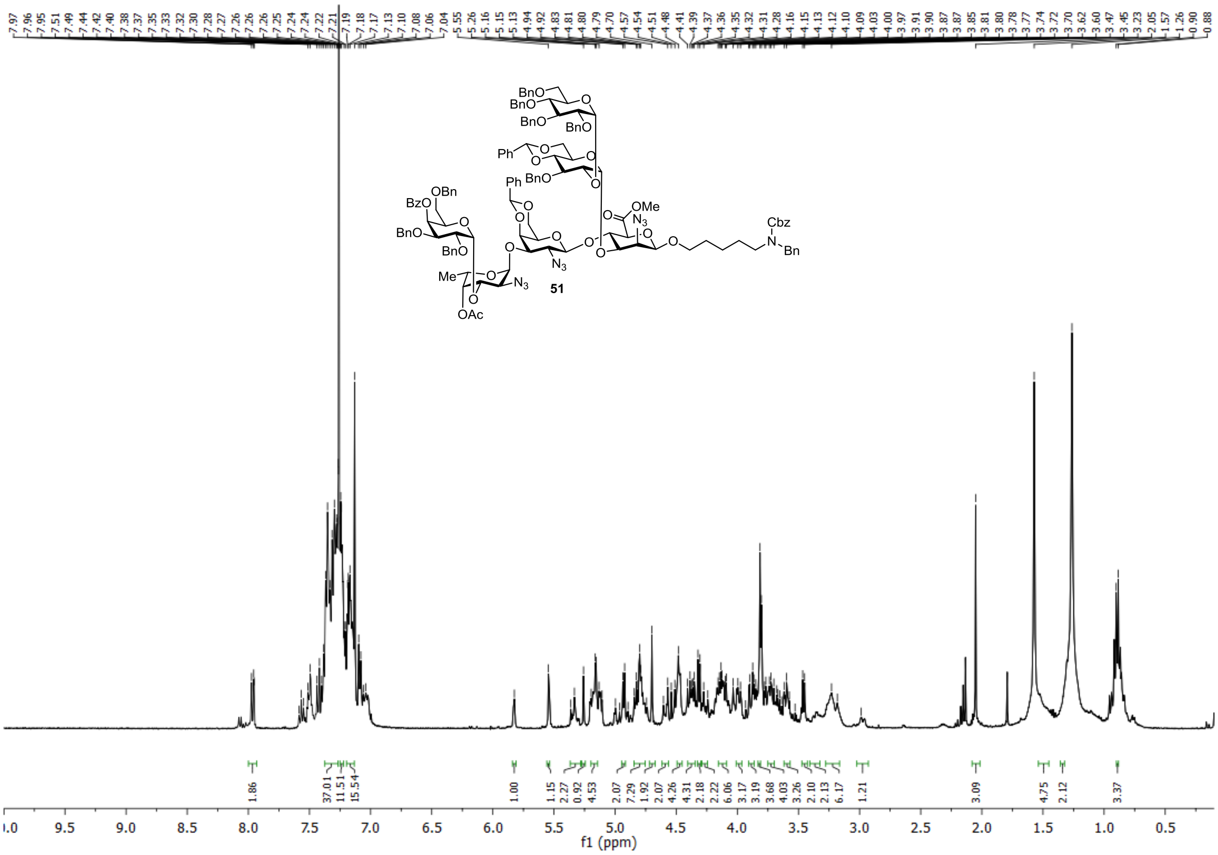
7.97 7.96 7.95 7.51 7.49 7.44 7.42 7.40 7.38 7.37 7.35 7.33 7.32 7.30 7.28 7.27 7.26 7.26 7.25 7.24 7.24 7.22 7.21 7.19 7.18 7.17 7.13 7.10 7.08 7.06 7.04 5.55 5.26 5.16 5.15 5.13 4.94 4.92 4.83 4.81 4.80 4.79 4.70 4.57 4.54 4.51 4.48 4.41 4.39 4.37 4.36 4.35 4.32 4.31 4.28 4.16 4.15 4.13 4.12 4.10 4.09 4.03 4.00 3.97 3.91 3.90 3.87 3.85 3.81 3.80 3.78 3.77 3.74 3.72 3.70 3.62 3.60 3.47 3.45 3.23 2.05 1.57 1.26 0.90 0.88
BnO
BnO
BnO
BnO
Ph
BnO
Ph
BzO
OBn
BnO
BnO
OMe
N3
Cbz
N
Bn
N3
Me
N3
51
OAc
1.86 37.01 11.51 15.54 1.00 1.15 2.27 0.92 4.53 2.07 7.29 1.92 2.07 4.26 4.31 2.18 2.22 6.06 3.17 3.19 3.68 4.03 3.26 2.10 2.13 6.17 1.21 3.09 4.75 2.12 3.37
9.5 9.0 8.5 8.0 7.5 7.0 6.5 6.0 5.5 5.0 4.5 4.0 3.5 3.0 2.5 2.0 1.5 1.0 0.5
f1 (ppm)

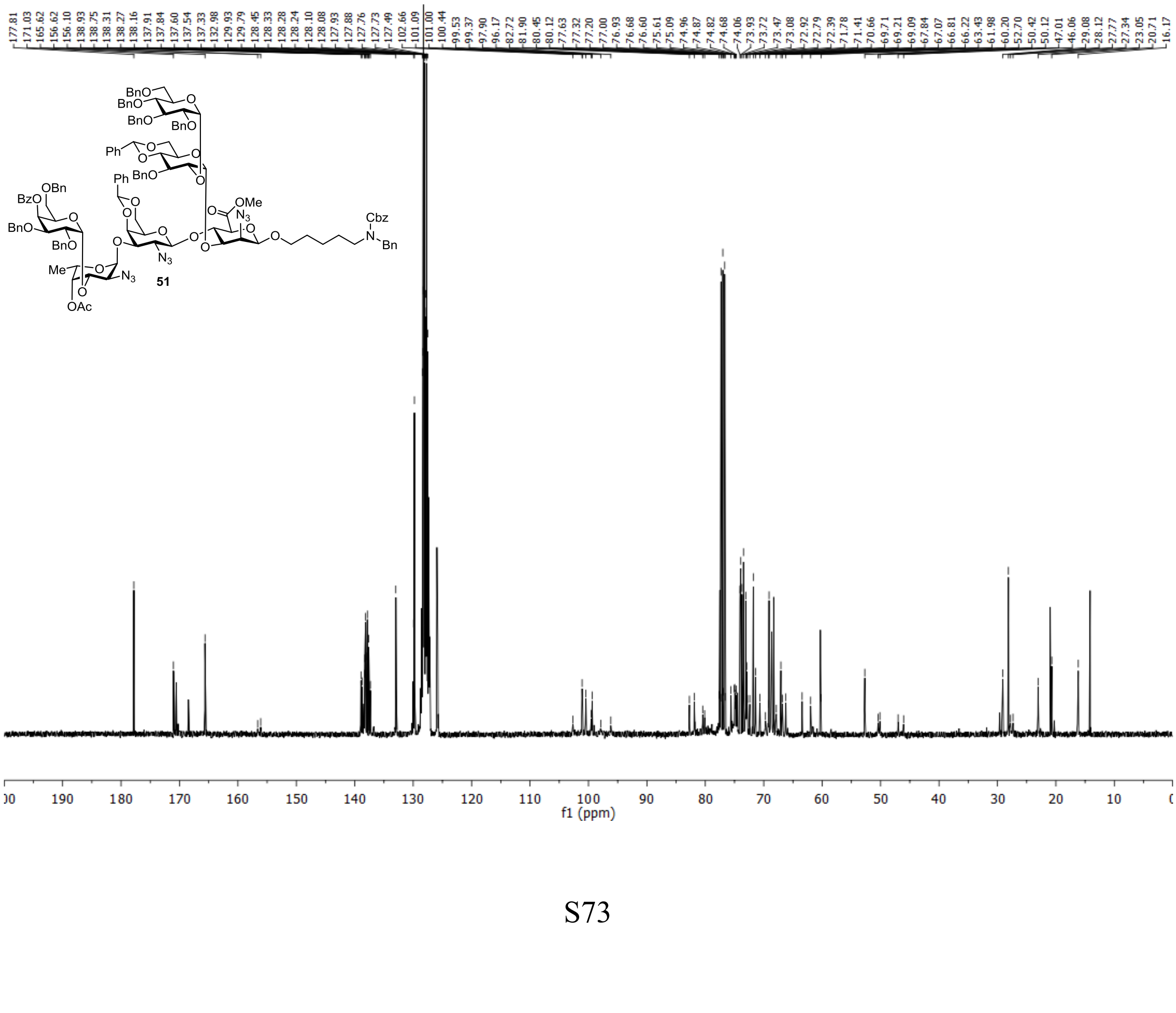
177.81 171.03 165.62 156.62 156.10 138.93 138.75 138.31 138.27 138.16 137.91 137.84 137.60 137.54 137.33 132.98 129.93 129.79 128.45 128.33 128.28 128.24 128.10 128.08 127.93 127.88 127.76 127.73 127.49 102.66 101.09 101.00 100.44 99.53 99.37 97.90 96.17 82.72 81.90 80.45 80.12 77.63 77.32 77.20 77.00 76.93 76.68 76.60 75.61 75.09 74.96 74.87 74.82 74.68 74.06 73.93 73.72 73.47 73.08 72.92 72.79 72.39 71.78 71.41 70.66 69.71 69.21 69.09 67.84 67.07 66.81 66.22 63.43 61.98 60.20 52.70 50.42 50.12 47.01 46.06 29.08 28.12 27.77 27.34 23.05 20.71 16.17
BnO
BnO
BnO
BnO
Ph
BnO
Ph
BzO
OBn
BnO
BnO
OMe
N3
Cbz
N
Bn
N3
Me
N3
51
OAc
190 180 170 160 150 140 130 120 110 100 90 80 70 60 50 40 30 20 10
f1 (ppm)

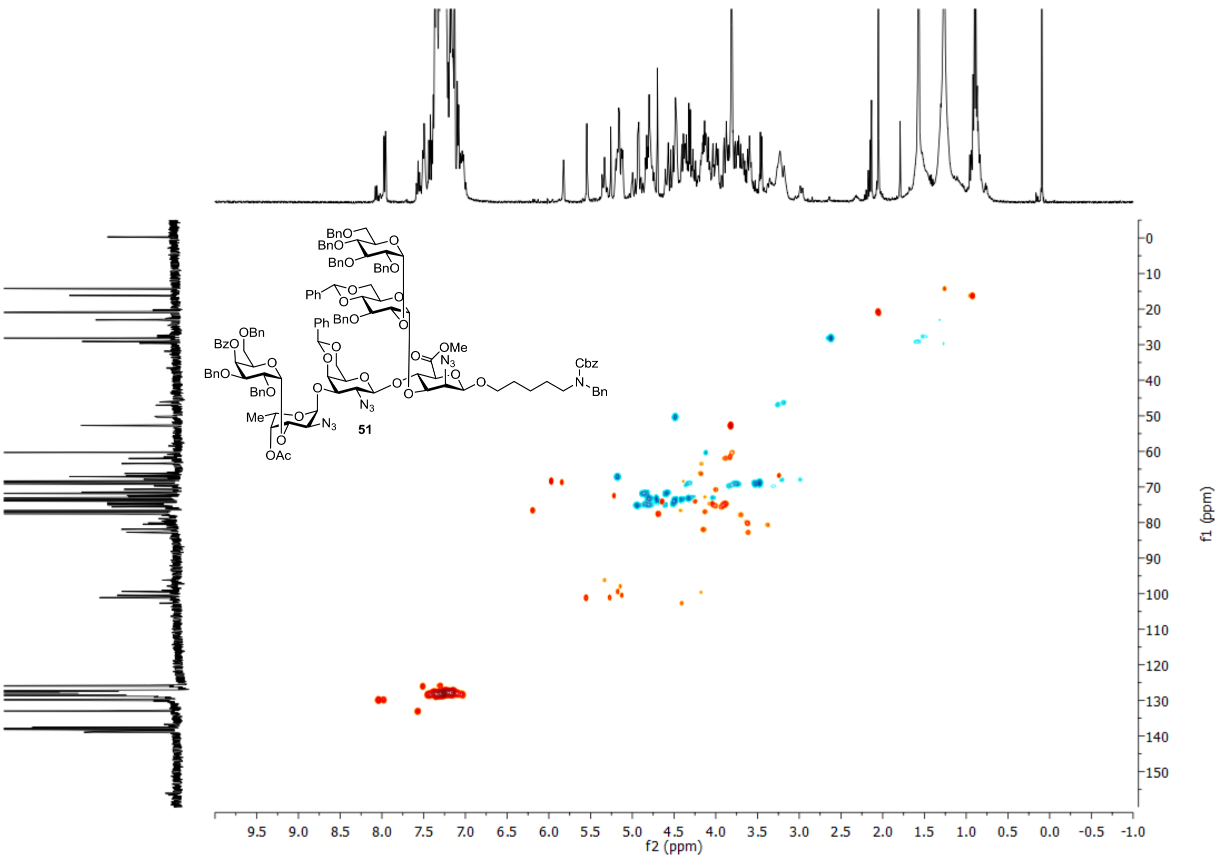

51
f2 (ppm)
f1 (ppm)

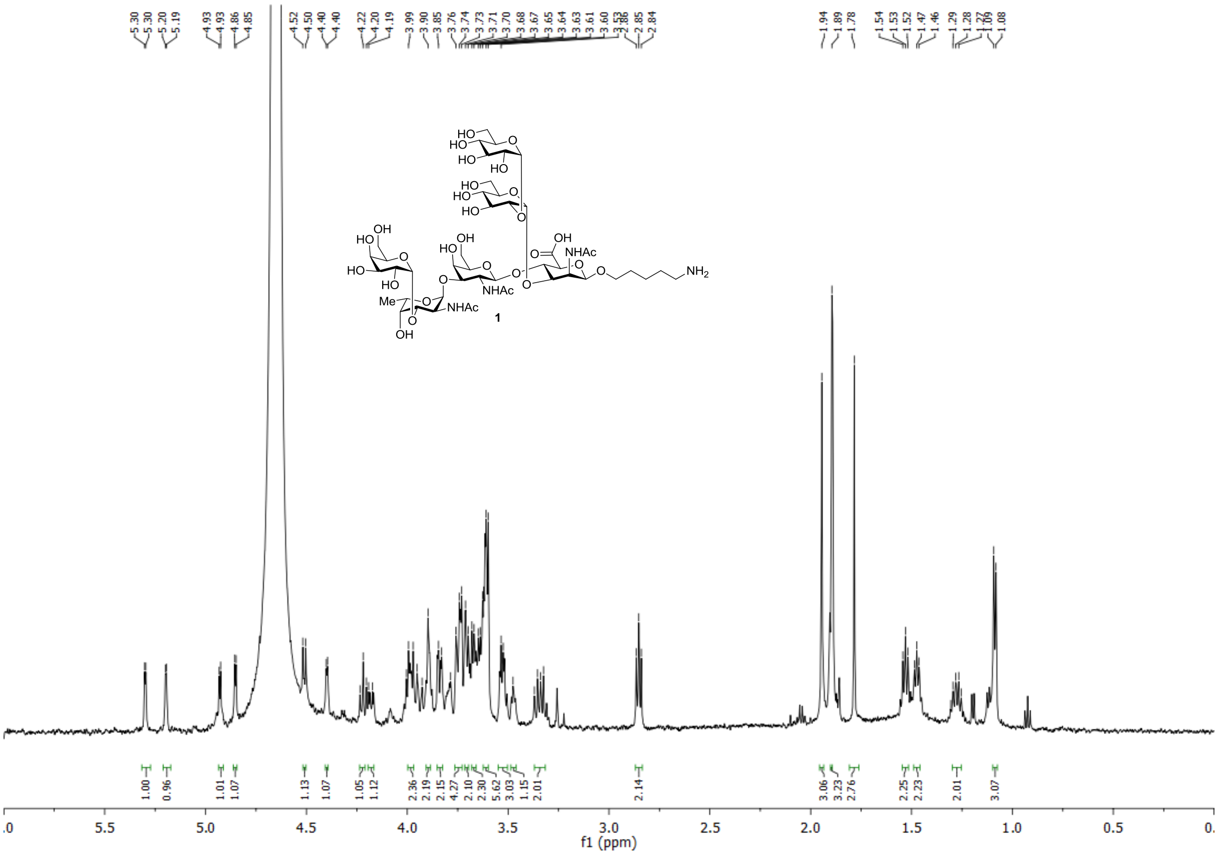

1
f1 (ppm)

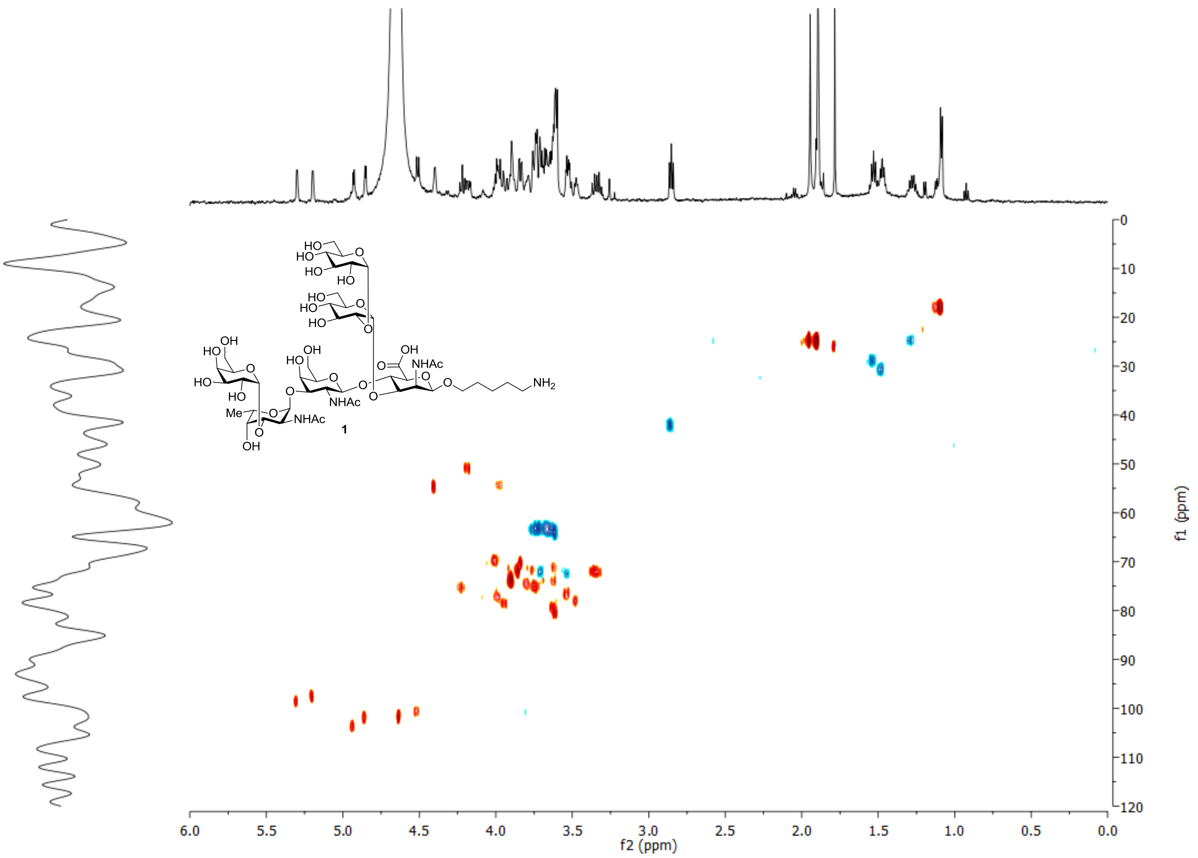

NHAc
NH2
1
f1 (ppm)
f2 (ppm)

References

[1] Mo, K. F.; Li, H.; Mague, J. T.; Ensley, H. E. *Carbohydr Res* **2009**, *344*, 439
[2] S. J. Danishefsky, S. Hu, P. F. Cirillo, M. Eckhardt and P. H. Seeberger, *Chem.–Eur. J.*, **1997**, *3*, 1617
[3] Parson, T. B.; Moir, J. W. B.; Fairbanks, A. J. *Org Biomol Chem* **2009**, *7*, 3128
[4] Litjens, R. E.; Leeuwenburgh, M. A.; van der Marel, G. A.; van Boom, J. H. *Tetrahedron Lett* **2001**, *42*, 8693.
[5] Tiehai L.; Lina G.; Yan Z.; Jiajia W.; Zhenxing Z.; Jing L.; Wenpeng Z.; Jianping L.; Wei Z.; Peng G. W.; *Bioorg. Med. Chem.*, **2011**, *19*, 2136
[6] Bedini, E.; Cirillo, L.; Parrilli, M. *Carbohydr Res* **2012**, *349*, 24
[7] Sarkar, K.; Mukherjee, I.; Roy, N. *J Carbohyd Chem* **2003**, *22*, 95
[8] Demchenko, A. V.; Rousson, E.; Boons, G. J. *Tetrahedron Lett* **1999**, *40*, 6523
